# *Theileria parva* antigens recognized by CD8+ T cells show varying degrees of diversity in buffalo-derived infected cell lines

**DOI:** 10.1017/S0031182018000264

**Published:** 2018-05-06

**Authors:** Tatjana Sitt, Roger Pelle, Maurine Chepkwony, W. Ivan Morrison, Philip Toye

**Affiliations:** 1 International Livestock Research Institute (ILRI), P.O. Box 30709, Nairobi, 00100, Kenya; 2 Biosciences Eastern and Central Africa (BecA) – International Livestock Research Institute, P.O. Box 30709, Nairobi, 00100, Kenya; 3 The Roslin Institute, The University of Edinburgh, Midlothian, EH25 9RG, Scotland

**Keywords:** African buffalo, CD8+ cytotoxic T cell antigens, epitope, genetic diversity, *Theileria parva*

## Abstract

The extent of sequence diversity among the genes encoding 10 antigens (Tp1–10) known to be recognized by CD8^+^ T lymphocytes from cattle immune to *Theileria parva* was analysed. The sequences were derived from parasites in 23 buffalo-derived cell lines, three cattle-derived isolates and one cloned cell line obtained from a buffalo-derived stabilate. The results revealed substantial variation among the antigens through sequence diversity. The greatest nucleotide and amino acid diversity were observed in Tp1, Tp2 and Tp9. Tp5 and Tp7 showed the least amount of allelic diversity, and Tp5, Tp6 and Tp7 had the lowest levels of protein diversity. Tp6 was the most conserved protein; only a single non-synonymous substitution was found in all obtained sequences. The ratio of non-synonymous: synonymous substitutions varied from 0.84 (Tp1) to 0.04 (Tp6). Apart from Tp2 and Tp9, we observed no variation in the other defined CD8+ T cell epitopes (Tp4, 5, 7 and 8), indicating that epitope variation is not a universal feature of *T. parva* antigens. In addition to providing markers that can be used to examine the diversity in *T. parva* populations, the results highlight the potential for using conserved antigens to develop vaccines that provide broad protection against *T. parva*.

## Introduction

Cattle constitute an important source of livelihood for African pastoralists, whether for societal status, income generation through meat or milk production and sale, or protection against additional economic hardships (Anderson, 2003; Mwacharo and Drucker, 2005; Ilatsia *et al.* 2011). The implementation of preventative measures against disease is imperative to maintain herd health and productivity, including those situations where livestock share grazing habitat with wild animal populations (Grootenhuis and Olubayo, 1993; Sitt *et al.* 2015). Vaccination is one of the most effective methods of disease prevention. The development of new or improved vaccines is aided by a clear understanding of the molecular interactions between the host and pathogen, including potential genetic diversity in the parasite antigens that induce protective immune responses. Antigenic diversity and antigen variation are exploited by pathogens for immune evasion in diseases such as malaria, trypanosomiasis and Lyme disease (Frank, 2002).

The protozoan parasite *Theileria parva* (*T. parva*) is of particular concern for pastoralists in sub-Saharan Africa. It is an apicomplexan parasite which induces lymphoproliferative disease in cattle and is transmitted by tick vectors, predominantly *Rhipicephalus appendiculatus*. Infections in cattle may be initiated by ticks that have acquired infection from other cattle or from African buffalo (*Syncerus caffer*) and these give rise to distinct clinical syndromes, referred to as East Coast fever (ECF) and Corridor disease (CD), respectively (Neitz *et al.* 1955; Barnett and Brocklesby, 1966). In both cases, the disease causes severe economic losses through morbidity and mortality throughout a large part of eastern, southern and central Africa (Conelly, 1998). A vaccination procedure used to protect cattle from ECF, called the Infection and Treatment Method (ITM), is successfully employed in parts of Zambia, Tanzania, Uganda, Malawi and Kenya, but generally only where pasture is grazed predominantly by cattle (Uilenberg *et al.* 1977; Morzaria *et al.* 2000). The most commonly used version of the ITM vaccine, which comprises a mixture of three *T. parva* isolates and is known as the Muguga cocktail, has been shown not to protect cattle introduced into an area previously grazed only by buffalo (Sitt *et al.* 2015).

Parasite-specific CD8+ T cells, which recognize epitopes presented on the surface of *T. parva*-infected lymphoblasts, have been shown to play a key role in mediating protection in animals immunized by ITM (McKeever *et al.* 1994). Immunity induced by ITM with one parasite isolate frequently does not provide complete protection against challenge with other parasite isolates and lack of protection has been shown to correlate with parasite strain specificity of the CD8+ T cell responses (Taracha *et al.* 1995). Ten antigens expressed by the intra-lymphocytic schizont stage of the parasite, designated Tp1–Tp10, have been described as targets of CD8+ T cells from immune cattle, and thus far, CD8+ T cell epitope regions have been defined for eight of these antigens (Gardner *et al.* 2005; Graham *et al.* 2006, 2007; Pelle *et al.* 2011; Hemmink *et al.* 2016). Sequence analyses of the genes encoding two of these antigens, Tp1 and Tp2, have revealed extensive sequence diversity, which was particularly pronounced in buffalo-derived parasites (Pelle *et al.* 2011). Specific CD8+ T cell responses to both antigens in ITM-immunized cattle have been shown to be parasite strain-specific, although the role of these antigens in immune protection has not been formally demonstrated (MacHugh *et al.* 2009; Connelley *et al.* 2011). A more limited analysis of the Tp9 gene has also revealed marked sequence diversity (Hemmink *et al.* 2016). Diversity in the genes encoding the remaining *T. parva* antigens (TpAg) has not been examined in detail.

In an effort to increase our understanding of the overall antigenic diversity in *T. parva*, we analysed the sequences of genes encoding 10 TpAg in infected cell lines derived predominantly from buffalo, which previous studies have indicated harbour greatest parasite genetic diversity. The results confirmed the high level of diversity among the Tp1, Tp2 and Tp9 antigens, suggesting that these would be most useful for parasite strain typing. On the other hand, the much more limited diversity displayed by the other antigens, especially in their known CTL epitopes, indicates that these would be the more preferable for inclusion in a subunit vaccine.

## Materials and methods

### Parasite isolates

The samples used in these studies are listed in Tables 1–3. Nineteen of the buffalo-derived stabilates collected from several regions in Kenya have been described previously (Conrad *et al.* 1987; Grootenhuis *et al.* 1987; Baldwin *et al.* 1988; Pelle *et al.* 2011) (Supplementary Table S2). Sample BD19 from buffalo 5012 was recovered from the ILRI biorepository, although the history of the buffalo was not found. Samples from three cattle-derived infected cell lines were also included in the analysis for comparison – samples CD16 and CD17 have been described previously (Pelle *et al.* 2011) and originate from Nyairo, Kenya, while the third, CD28, is from Ngong, Kenya. Tp9 analysis was conducted on DNA from an additional 12 cell lines from seven buffalo-derived field stabilates. Polymerase chain reaction (PCR) amplicons from these DNA samples and from fifteen of the stabilates described above (Table 3) were cloned and sequenced.

**Table 1.** Allele and protein variants of Tp1–4 observed in the buffalo- and cattle-derived *T. parva* stabilates

Sample ID	Stabilate name	Tp1^a^		Tp2^a^		Tp3		Tp4	
		Allele TP03_0849^b^	Protein XP_762973	Allele TP01_0056	Protein XP_765583	Allele TP01_0868	Protein XP_763892	Allele TP03_0210	Protein XP_763228
Buffalo-derived *T. parva*									
BD1	Mara 3	*16*	*15*	*8*	*8*	**6^c^**	**4**	**2**	1
BD2	Mara 4	*1*	*1*	*9*	*9*	**10**	**9**	–	–
BD3	Mara 18	*17*	*16*	*10*	*10*	**7**	**6**	**3**	**4**
BD4	Mara 30	*1*	*1*	*11*	*11*	**6**	**4**	**3**	**4**
BD5	Mara 32	*1*	*1*	*12*	*12*	**6**	**4**	**9**	**5**
BD6	Mara 42	*18*	*17*	*13*	*13*	**8**	**9**	**8**	**6**
BD7	Mara 6998	*1*	*1*	*14*	*14*	**3**	**8**	**12**	**3**
BD8	Mara 6999	*19*	*18*	*7*	*7*	**2**	**5**	**5**	1
BD9	Mara 7001	*20*	*14*	*15*	*15*	**4**	**2**	**3**	**4**
BD10	Mara 7546	*21*	*19*	*16*	*16*	**11**	**9**	**5**	1
BD14	Buffalo 7014	*1*	*1*	*20*	*1*	**5**	**3**	–	–
BD15	Buffalo 7698	*14*	*12*	*21*	*20*	**10**	**9**	**13**	**3**
BD16	Buffalo 7344	*24*	*21*	*7*	*7*	**5**	**3**	**3**	**4**
BD17	Mara 41	–	–	–	–	5*	3*	**11^d^**	1
BD18	Buffalo 4740	1	1	**44**	**43**	1*	1*	**13**	**3**
BD19	Buffalo 5012	1	1	1	1	13*^e^	9*	1	1
BD20	Buffalo 5489	**36**	13	**48**	**47**	10*	9*	**13**	**3**
BD21	Buffalo 6666	**40**	**36**	**48**	**47**	3*	8*	**2**	1
BD22	Buffalo 6807	13	11	–	–	–	–	**4.1^f^**	1.1
BD23	Buffalo 6818	13	11	–	–	10*	9*	**4**	1
BD24	Buffalo 6819	**37**	**35**	**45**	**42**	10*	9*	**10**	1
BD25	6998 cl. 11	**38**	**37**	**49**	**45**	**3**	**8**	**12**	**3**
BD26	Buffalo 7065	–	–	**–**	–	–	–	**9.1**	**5**
Cattle-derived *T. parva*									
CD16	Tc867 BT 106	1	1	1	1	1	1	1	1
CD17	Tc841 IL 57	2	2	**47**	**46**	**9**	**7**	**7**	**3**
CD28	Tc120F344	**39**	17	**46**	**44**	**12**	**9**	**6**	**2**

a We have previously published (Pelle *et al.* 2011) the Tp1 and Tp2 results for samples BD1-BD16. They are included here in italics for completeness.

b The letters-numbers in this row indicate the reference sequence in the NCBI Genbank.

c Newly identified alleles and protein variants are in boldface.

d Although we were unable to determine if the sequence is novel due to four unknown (mixed) nucleotides, the sequence is considered novel due to unique nucleotides in other sequence regions.

e Unique, validated alleles or protein variants obtained from partial sequences which are their own variant.

f A variant with 0.1 after the variant number indicates a sequence which is identical to the parent variant but has 1–4 unresolved nucleotides which prevents its being regarded as completely identical to the parent allele (e.g. 4 if labelled 4.1, as in BD22, Tp4).

* partial sequence as defined by only having either the forward or reverse sequence of the allele available for analysis (ie: no complete consensus sequence).

**Table 2.** Allele and protein variants of Tp5–8 and Tp10 observed in the buffalo- and cattle- derived *T. parva* stabilates

Sample ID	Stabilate name	Tp5		Tp6		Tp7		Tp8		Tp10	
		Allele TP02_0767^a^	Protein XP_765334	Allele TP01_0188	Protein XP_765715	Allele TP02_0244	Protein XP_764810	Allele TP02_0140	Protein XP_764709	Allele TP04_0772	Protein XP_764408
Buffalo-derived *T. parva*											
BD1	Mara 3	**3**^b^	1	**13**	1	**7**	**2**	**12**	**7**	**11**	1
BD2	Mara 4	1	1	**3**	**2**	**8.1, 2.1**^c^	**2**^d^	–	–	–	–
BD3	Mara 18	1	1	**6**	1	**3**	**2**	–	–	**10**	1
BD4	Mara 30	2*^e^	1*	**7**	1	**2**	**2**	**2**	**4**	**10.1**	1
BD5	Mara 32	1*	1*	**8**	1	**7**	**2**	**12**	**7**	**6**	**5**
BD6	Mara 42	1	1	**13.1**	1.1	**2**	**2**	**11**	**7**	1	1
BD7	Mara 6998	**4**	**2**	**1**	1	**8**	**2**	**6**	**6**	**12**	**5**
BD8	Mara 6999	1	1	**2**	1	**7**	**2**	**5**	**6**	**7**	**2**
BD9	Mara 7001	1	1	**4**	1	**7**	**2**	–	–	**10**	1
BD10	Mara 7546	1	1	**5**	1	**7**	**2**	–	–	**11**	1
BD14	Buffalo 7014	1	1	**9**	1	**8**	**2**	**11**	**7**	13*^e^	1.1, 4.1, 5.1, 6.1*^f^
BD15	Buffalo 7698	**3**	1	**12**	1	8*	2*	**4**	**5**	**3**	**4**
BD16	Buffalo 7344	–	–	**11**	1	**8**	**2**	**6**	**6**	–	–
BD17	Mara 41	–	–	–	–	**5.1**	**2**^d^	**2**	**4**	**10**	1
BD18	Buffalo 4740	–	–	**12.1^g^**	1	**2**	**2**	**3**	**3**	**10**	1
BD19	Buffalo 5012	1*	1*	**9.1**	1	1	1	1	1	1	1
BD20	Buffalo 5489	–	–	**13**	1	**7**	**2**	**7**	**2**	**6**	1.1, 4.1, 5.1, 6.1^f^
BD21	Buffalo 6666	–	–	**13**	1	**7**	**2**	**9**	**7**	5*^e^	1.1, 4.1, 5.1, 6.1*^f^
BD22	Buffalo 6807	1*	1*	–	–	**5**	**2**	**10.1, 11.1**	**6.1, 7.1**	9*^e^	1.1, 4.1, 5.1, 6.1*^f^
BD23	Buffalo 6818	6*^e^	1*	**13**	1	**8.1**	**2**	**10**	**7**	–	–
BD24	Buffalo 6819	5*^e^	3*^e^	**10**	1	**9.1**^g^	**3**^d^	**11**	**7**	**8**	**3**
BD25	6998 cl. 11	–	–	**11**	1	**8**	**2**	**6**	**6**	**11**	1
BD26	Buffalo 7065	–	–	13*	1*	**6**	**2**	**11**	**7**	–	–
Cattle-derived *T. parva*											
CD16	Tc867 BT 106	–	–	1	1	–	–	1	1	1	1
CD17	Tc841 IL 57	–	–	1	1	1*	1*	1	1	**2**	**6**
CD28	Tc120 F344	–	–	–	–	4*^e^	1*	**8**	**7**	**4**	1.1, 4.1, 5.1, 6.1^f^

a The letters-numbers in this row indicate the reference sequence in the NCBI Genbank.

b Newly identified alleles and protein variants are in boldface.

c A variant with 0.1 after the variant number indicates a sequence which is identical to the parent variant but has 1–4 unresolved nucleotides which prevents its being regarded as completely identical to the parent allele (e.g. 8 if labelled 8.1, as in BD2, Tp7).

d Although the nucleotide sequence had unknown (mixed) nucleotides (i.e. S = C or G, R = A or G, Y = C or T), upon translation, these nucleotides resulted in synonymous protein sequence.

e Unique, validated alleles or protein variants obtained from partial sequences which are their own variant.

f These sequences were shorter than the rest and could have been any of the variants noted in the table above as these sequence areas were an identical overlap with variants 1, 4, 5 and 6.

g Although we were unable to determine if the sequence is novel due to unknown (mixed) nucleotides, the sequence is considered novel due to unique nucleotides in other sequence regions.

* Partial sequence as defined by only having either the forward or reverse sequence of the allele available for analysis, if also labelled with *.

**Table 3.** Allele and protein variants of Tp9 observed in the buffalo- and cattle-derived theileria stabilates

Sample ID	Stabilate name	Genomic DNA^a^			Cloned DNA^a^		
		Allele TP02_0895^b^	Protein XP_765463^b^	Epitope	Allele	Protein	Epitope
Buffalo-derived *T. parva*							
BD1	Mara 3	4	22	AKFPGMKKG	**2**	4	**NKFKSMGIG**
BD2	Mara 4	–	–	**–**	**3**	**5**	AKFPGMKKG
BD3	Mara 18	**13^c^**	**15**	EKFQRMGIG	**7**	**19**	AKFPGMKKG
BD4	Mara 30	**15**	**24**	EKFQRMGIG	**13**	**15**	EKFQRMGIG
BD5	Mara 32	**–**	**–**	–	**2**	**4**	**NKFKSMGIG**
BD6	Mara 42	4	22	AKFPGMKKG	10	16	EKFQRMGIG
BD7	6998	18	**6**	EKFQRMGIG	**3**	**5**	AKFPGMKKG
BD8	6999	–	**–**	–	17	**18**	EKFQRMGIG
BD9	7001	**–**	**–**	–	**14**	**23**	EKFQRMGIG
BD10	7546	**–**	**–**	–	**16**	**27**	EKFKHMGIG
BD14	7014	**–**	**–**	–	**12**	**2**	**NKFKSMGIG**
BD15	7698	**–**	**–**	–	**2**	**4**	**NKFKSMGIG**
BD16	7344	**6**	**17**	**AKFPNLKAR**	**9**	**8**	**EKFKRMGMK**
BD17	Mara 41	–	–	–	–	–	–
BD18	4740	**11**	**3**	**NKFKSMGIG**	–	–	–
BD19	5012	1	1	AKFPGMKKS	–	–	–
BD20	5489	–	–	–	–	–	–
BD21	6666	–	–	–	–	–	–
BD22	6807	–	–	–	–	–	–
BD23	6818	–	–	–	–	–	–
BD24	6819	–	–	–	–	–	–
BD25	6998 cl. 11				**6**	**17**	**AKFPNLKAR**
BD26	7065	–	–	–	–	–	–
BD27	7014, st.3506^a^	–	–	–	4	22	AKFPGMKKG
BD28	7014, st.3570^a^	–	–	–	1	1	AKFPGMKKS
BD29	7014, st.3081^a^	–	–	–	26	7	EKFKHMRIG
BD30	7014, st.3081^a^	–	–	–	25	11	EKFKRMGMK
BD31	7014, st. 3081^a^	–	–	–	24	12	EKFKRMGMK
BD32	7014, st.3081^a^	–	–	–	21	26	NKFPGMKKG
BD33	7014, st.3081^a^	–	–	–	23	25	AKFPGMKKG
BD34	7014, st. 3081^a^	–	–	–	19	9	NKFKHMRIG
BD35	7752, st. 4110^a^	–	–	–	27	13	EKFKHMGIG
BD36	7344, st. 3591^a^	–	–	–	28	14	EKFKHMGIG
BD37	7065, st. 3120^a^	–	–	–	20	21	EKFQRMGIG
BD38	6999, st. 3123^a^	–	–	–	22	20	AKFPGMKKG
BD39	6998, cl.19^a^	–	–	–	18	6	EKFQRMGIG
Cattle-derived *T. parva*							
CD16	Tc 867 Bt106	1	1	AKFPGMKKS	–	–	–
CD17	Tc 841 IL57	**–**	–	–	**8**	–	EKFQRMGIG
CD28	Tc 120 F344	**–**	**–**	**–**	**5**	**10**	**RKFQRMGIG**

a Genomic DNA sequences were obtained directly from purified DNA; cloned DNA sequences were obtained from plasmids into which genomic DNA had been cloned.

b Reference sequence accession number.

c Newly identified alleles, protein variants and epitopes are in boldface.

### DNA extraction

Sixteen buffalo-derived DNA samples have previously been described (Pelle *et al.* 2011). DNA from the additional samples (see *Parasite isolates*) was extracted using DNeasy Blood and Tissue Kit (Qiagen, USA). An aliquot of DNA was diluted to 100 ng *µ*L^−1^ with either AE buffer or RNase/DNase free water and frozen. The stock DNA was stored at −20 °C.

### PCR and sequencing of stabilates

PCR amplicons were generated from genes encoding the 10 previously described *T. parva* antigens (TpAg). PCR master mixes comprised 10 *µ*L 10 ×  buffer (Applied Biosystems), 10 *µ*L 25 mm MgCl_2_, 1 *µ*L 25 mm dNTP, 1 *µ*L 100 pmole *µ*L^−1^ forward and reverse primers, 0.5 *µ*L Ampli-Taq Gold (Applied Biosystems) and H_2_O added to a final volume of 30ul, containing between 2.5 and 5 *µ*L of 100 ng *µ*L^−1^ gDNA. Cycling conditions can be found in Supplemental Table 1. Tp1 and Tp2 primers have previously been described (Pelle *et al.* 2011). TpAg information including Genebank accession numbers of references and epitope sequences can be found in Tables 1–4. Primers for Tp3, Tp4, Tp5, Tp6, Tp7, Tp8, Tp9 and Tp10 can be found in Table 5.

**Table 4.** Tp 2 epitopes observed in the buffalo- and cattle-derived theileria stabilates

Sample ID	Epitope 1	Epitope 2	Epitope 3	Epitope 4	Epitope 5	Epitope 6
BD18	TNEELKKLGMV (V16)	EGLDMEALF (V12)	KTSKGMTKVGR (V15)	FGQSIKCVA (V8)	QSIKCVAQH (V7)	KGDVPNPCDW (V10)
BD19	SHEELKKLGML (V1)	DGFDRDALF (V1)	KSSHGMGKVGK (V1)	FAQSLVCVL (V1)	QSLVCVLMK (V1)	KTSIPNPCKW (V1)
BD20, 21	SDNELDTLGLL (V4)	PDLDKNRLF (V2)	LTSHGMGKIGR (V3)	LAASIKCVS (V3)	**ASIKCVSQH (V20)**^a^	**KPSIPNPCDW (V19)**
BD24	**SDSELETLGML (V26)**^a^	PDPDKQRLF (V8)	LTSKAMSTVGK (V11)	**FAQSIYCVA (V21)**	QSIYCVANN (V10)	KGDVPNPCQW (V8)
BD25	SDDELRKLGML (V15)	DGFDRDALF (V1)	KSSHGMGKVGR (V14)	FVESIMCVI (V14)	ESIMCVIKK (V14)	KEDVPNPCDW (V3)
CD16	SHEELKKLGML (V1)	DGFDRDALF (V1)	KSSHGMGKVGK (V1)	FAQSLVCVL (V1)	QSLVCVLMK (V1)	KTSIPNPCKW (V1)
CD17	SDNELDTLGLL (V4)	PDLDKNRLF (V2)	**LTSHGMGKIAR (V25)**	LAASIKCVS (V3)	ASIKCVSHH (V3)	KPSVPNPCDW (V2)
CD28	**TEEELKKMGMV (V27)**	**EGFDKEKLF (V22)**	**KSSKSMGIVGR (V24)**	**FVQSIMCVI (V20)**	**QSIMCVINK (V21)**	**VNDIPNPCKW (20)**

a Newly identified epitope sequences are in boldface.

Epitope variant number is found in (). Original epitope variant numbers are defined in Pelle *et al.* 2011. Epitope variant numbers are separate from protein variant numbers.

**Table 5.** Comparative sequence information on TpAg1–10 observed in the buffalo- and cattle-derived theileria stabilates

Antigen	Primers 5’ – 3’ Forward Reverse	Predicted length of amplicon^a^ (%)	Lengths of readable sequence	Total no. sequences obtained	Total no. SNP locations^b^	Total no. alleles^c^^,^^d^	Total no. non-synonymous SNP locations	Total no. protein variants^c,d^	Total no. epitope variants
Tp1	ATGGCCACTTCAATTGCATTTGCC GAAGCTCATAAATATTTCATT	387 (22)	363–399	11	19	8	16	8	2
Tp2	ATGAAATTGGCCGCCAGATTA GAAGCCTCCGGCACTTCATAG	483 (47)	456–480	9	220	7	92	7	See Table 2
Tp3	ATGAAATTAAATACTATCGCAATAG ACCTTCACTAAGAGTCCAGACGATAAT	735 (89)	686–726	24	26	13	12	9	n/a
Tp4	CCAATTGGTGATTTAGCAACA CTCCGTAAATCATGTTAATGGAGT	692 (27)	386–422	24	36	13	5	6	1
Tp5	ATGAGGAAGCGAGTTTGGG CGATATCGATGATTTATAA	238 (31)	200–238	16	26	6	3	3	1
Tp6	ATGGCTCAGATTCCTGTTG CTCTTACTCTCAACTGATAAATAA	1005 (92)	569–833	23	23	13	1	2	n/a
Tp7	ATGGAGGCACTGCAAGCAGGC GTCACTGACGAGGAGGAGAAAAAG	417 (14)	299–332	25	33	9	4	3	1
Tp8	ATGCTTGGAAATCATGTCATGGGA CTCCTTCGCAGTGTACGCAGTTTAA	1275 (81)	931–1109	22	19	12	6	7	1
Tp9	ATGAATGTTCTAACTACTGG CGTAATAAACCATGGACAAAACAATAA	958 (95)	326–1057	37	>100	28	>100	27	12
Tp10	GACTACGCTCTTCTTACCAC CCATCCAGGAGTGTAATGCC	916 (89)	505–824	22	24	13	7	6	n/a

a The sequence length of the predicted amplicon from the reference sequence, excluding primers, is shown. The figures in brackets represent the length of the amplicon as a percentage of the length of the coding sequence from the reference gene, including introns.

b SNPs within introns are not included.

c Alleles or protein variants which have ambiguous residues (labelled X.1 in Table 1) are not included, unless they are unique, validated variants.

d Unique, validated alleles or protein variants obtained from partial sequences are included if the constitute a variant.

PCR amplicons were purified either by centrifugal precipitation in PEG8000/MgCl_2_ or Qiagen PCR purification kit (Qiagen, USA) and samples eluted in Elution Buffer (EB) or molecular grade water. Purification *via* Qiagen kit was conducted according to manufacturer's protocol. Purification *via* PEG8000/MgCl_2_ centrifugation was conducted as briefly outlined below: 175 *µ*L of EB was added to 25*μ*L of PCR product and mixed thoroughly with 100 *µ*L 30% PEG8000/30 mm MgCl_2_. Mixtures were immediately centrifuged at 10 000 ***g*** for 20 min at room temperature after which the supernatant was removed and the pellet was dissolved in 10 *µ*L water. 1 *µ*L was used to confirm recovery on a gel. Aliquots of PCR purified products were sequenced at the BecA-ILRI Hub, Nairobi, Kenya. Sanger sequencing was conducted on the purified PCR products using the GeneAmp^®^ 9700 system (Applied Biosystems). Additional samples (BD33-BD44,and BD26, Table 3) were processed and sequenced after cloning into pGEM-T Easy plasmid vector (Promega) and sequenced as previously described (Pelle *et al.* 2011). These additional Tp9 sequences have previously been submitted to Genbank with the accessions numbers JN828553, JN828535, JN828556, JN828557, JN828558, JN828559, JN828560, JN828561, JN828562, JN828563, JN828564 and JN828565, and JQ735950.

### Sequence analysis

Sequence analysis, alignments and prediction of encoded proteins were conducted using Geneious version 7.1.4 created by Biomatters (http://www.geneious.com) for all *T. parva* antigens, with variant alignments conducted using Clustal Omega (https://www.ebi.ac.uk/Tools/msa/clustalo/) or SCSC Biology WorkBench (http://seqtool.sdsc.edu/). Additional Tp9 sequences were translated into protein sequences using EMBOSS-Transeq software (Rice *et al.* 2000) and alignments performed using CLUSTALW version 2.0 (Larkin *et al.* 2007). TpAg epitope information has been described in detail elsewhere (Graham *et al.* 2006, 2008; Akoolo *et al.* 2008; Nene *et al.* 2012; Svitek *et al.* 2014). All sequence lengths described in this paper are post-editing for removal of both primer sequence and weak or mixed trace peaks at the sequence ends-this is expected with Sanger sequencing.

Sanger sequencing results were obtained in both the forward (sense) and reverse (anti-sense) directions for each sample. Consensus sequences for each sample were identified through detailed analysis of both forward and reverse sequence chromatograms and individual nucleotide trace signals. Occasionally sequence in both directions resulted in multiple traces (double peaks) at a specific nucleotide location. In these cases, the ‘mixed’ nucleotide received the corresponding IUPAC ambiguity code. These sequences are identified in Tables 1 and 2. No sequence had more than four ambiguous residues with the exception of Buffalo 5012, Tp6. Some ambiguous nucleotides were observed to be non-synonymous.

Where only one sequence from one direction was obtained, all trace peaks were required to have strong signal intensity before including the sequence in data analysis. We define these sequences as partial sequences in the paper and these partial sequences are noted with an *. Occasionally these partial sequences yielded novel single nucleotide polymorphism (SNP) variants due to SNPs. These sequences are indicated in the Tables.

To confirm sequence identity, a GenBank database search (nucleotide and protein BLAST) of all the consensus nucleotide and amino acid sequences was conducted (http://blast.ncbi.nlm.nih.gov/Blast.cgi). Additionally, the sequences were compared with available GenBank sequences *via* BLAST to determine if the sequences were novel or were a 100% match to previously published data. Sequences that did not have 100% identity with published sequences were considered novel. 100% identity with a sequence from BLAST was only considered if the query coverage was over 70%. A new gene allele, protein variant or epitope variant was also confirmed by alignments with previously published sequences, as described further below. Alignments also allowed for detection of sequence introns or insertions and deletions (indels) in the obtained sequences. Where applicable, introns were removed from the DNA sequences before translation into protein.

All amplicons were categorized into allele and protein variants based on the following criteria; (1) the presence of at least one SNP difference from any published sequence and (2) the presence of at least one single amino acid difference from any published sequence. It is recognized that variant categorization could change if longer sequences are obtained in the future.

Stabilate gene and protein sequence GenBank accession numbers can be found in Supplemental Table S2. Some Tp9 sequences had previously been deposited in GenBank, but had not undergone formal analysis in a publication and were included in this paper.

## Results

A total of 23 DNA samples from infected cell lines derived from buffalo, three samples from cattle-derived (non-buffalo-associated) cultured infected cell lines and one cloned cell line obtained from a buffalo-derived stabilate were analysed. An additional 12 Tp9 sequences (from cell lines derived from buffalo) were analysed. Detailed information including primer sequence, length of PCR amplicons, edited sequence length, number of SNPs, nucleotide and protein variants have been summarized for all Tp genes in Table 5; limited information of these specific parameters is given in the text. Amplicons were not generated for each gene from every sample and not all amplicons generated readable sequences (Tables 1–3). The Tp1 and Tp2 sequences from 16 of the cell lines mentioned in this study have been published previously (Pelle *et al.* 2011) and are included for completeness.

### Sequence analysis

The nucleotide and amino acid sequences are available as sequence alignments in Supplementary Tables S3–S12. GenBank accession numbers for these sequences are given in Supplementary Table 2. A summary of the findings for the data below can be found in Tables 1–5, and 6. Table 6 also shows the percentage of the full-length gene represented by the amplicon for each gene.

**Table 6.** Diversity within the individual antigens

TpAg	No. nucleotide variants/No. sequences obtained	No. protein variants/No. of sequences obtained.	No. protein variants/No. nucleotide variants	No. non-synonymous SNP locations/Total No. SNP locations
Tp1	**8/11 (0.73)**	**8/11 (0.73)**	**8/8 (1)**	**16/19 (0.84)**
Tp2	**7/9 (0.78)**	**7/9 (0.78)**	**7/7 (1)**	92/220 (0.42)
Tp3	13/24 (0.54)	9/24 (0.38)	9/13 (0.69)	12/26 (0.46)
Tp4	13/24 (0.54)	6/24 (0.25)	6/13 (0.46)	5/36 (0.14)
Tp5	6/16 (0.38)	3/16 (0.19)	3/6 (0.5)	3/26 (0.12)
Tp6	13/23 (0.57)	2/23 (0.09)	2/13 (0.15)	1/23 (0.04)
Tp7	9/25 (0.36)	5/25 (0.20)	3/9 (0.33)	4/33 (0.12)
Tp8	12/22 (0.55)	7/22 (0.32)	7/12 (0.58)	6/19 (0.32)
Tp9	**28/37 (0.76)**	**27/37 (0.73)**	**27/28 (0.96)**	NA^a^
Tp10	13/22 (0.59)	6/22 (0.27)	6/13 (0.46)	7/24 (0.29)

a Due to extensive gene diversity, it was not feasible to conduct this analysis.

Numbers in bold indicate the top three most diverse TpAg per group.

### Nucleotide variant diversity

As shown in Tables 1–3, between 5 and 12 novel variants were identified in all the antigens analysed. To compare the nucleotide diversity among the antigen genes, a nucleotide diversity ratio was determined for each gene by calculating the ratio of the number of nucleotide variants to the total number of sequences obtained for that gene (Table 6). Tp7 and Tp5 had the least allele diversity, with diversity ratios of 0.36 and 0.38, respectively, whereas Tp1, Tp2 and Tp9 had the highest allele diversity ratios, ranging from 0.73 to 0.78. Tp3, Tp4, Tp6, Tp8 and Tp10 were moderately diverse with ratios between 0.54 and 0.57.

We also examined the allele sequences for the presence of novel indels or introns. Only one novel Tp3 allele, v9, had the in-frame trinucleotide insertion AAA (amino acid K) found in the reference sequence and there are numerous indels throughout the Tp9 gene.

Interestingly, sequencing of the cloned Tp9 PCR products revealed six different sequences from one buffalo (BD27-BD34), suggesting that the buffalo was either infected concurrently or in succession with several strains of *T. parva.*

### Protein variant diversity

Between one and eight novel protein variants were identified in antigens analysed. As for the nucleotide diversity, we assessed the protein variant diversity by calculating the ratio of the number of protein variants to the number of sequences obtained for each antigen (Table 6). Tp1, Tp2 and Tp9 were again the most diverse with ratios between 0.73 and 0.78. On the other hand, Tp6 was by far the most conserved protein (ratio of 0.09) reflecting the fact that only one sample (BD2) expressed a Tp6 antigen which varied from the reference sequence. The remaining antigens showed moderate diversity ratios, which were all lower than the corresponding nucleotide diversity ratios. A more direct measure of this is the ratio of the number of protein variants to the number of nucleotide variants per antigen. For Tp1, Tp2 and Tp9, the ratios were 1, 1 and 0.96, as, with one exception for Tp9, every allelic variant encodes a unique protein. On the other hand, Tp6 showed a low ratio of protein to nucleotide variants of 0.15, with the remaining antigens revealing moderate ratios of 0.46–0.69. In addition to these observations, it was noted that the most common protein variant of Tp3 (v9) was found in nine of the 24 samples sequenced, while each of the five new Tp4 protein variants differed by only a single amino acid from the reference sequence.

### Synonymous vs non-synonymous substitutions

As another direct comparison of diversity among the genes, we determined the total number of SNP locations and the ratio of the number of non-synonymous to synonymous SNP locations for all antigens except Tp9. Tp9 had extensive diversity for which there is a large number of SNP locations and indels distributed throughout the gene. The Tp2 gene had by far the greatest number of SNP locations (220 or 46% of the predicted amplicon length) compared with the other genes which ranged from 19 to 36 SNP locations. Tp1 showed the lowest conservation of protein sequences with a ratio of 0.84, compared with Tp4, Tp5, Tp7 and especially Tp6, all of which showed ratios of less than 0.20. Remarkably, Tp6 differed at only one amino acid residue in the single protein sequence which differed from the reference sequence. We also noted that, among the Tp5 sequences, those from BD7 and BD24 expressed the majority of SNPs and shared a high degree of similarity, while the other novel Tp5 alleles differed from the reference sequence at 1, 2 or 3 residues only.

### Diversity in epitope sequences

CTL epitopes have not yet been defined for two (Tp3 and Tp6) of the 10 antigens included in this study while the recently reported epitope for Tp10 (Hemmink *et al.* 2016) lies outside the region sequenced in this paper. Remarkably, no epitope diversity was observed in Tp4, Tp5, Tp7, or Tp8. On the other hand, new epitope sequences were found at all six Tp2 epitope locations, with one sample (CD28) expressing new variants at all six locations (Table 4). Between 5 and 7, epitope variants were observed at each of the six epitope regions for the nine sequenced samples. For Tp9, of the 37 samples which were successfully sequenced, there were 11 variants of the single defined epitope and these were represented between 1 and 10 times within the sequences.

In all, the results indicate that the substantial epitope variation previously observed in Tp2 and Tp9 is not a universal feature of *T. parva* CTL antigens.

## Discussion

The studies reported in this paper were undertaken to evaluate the extent of diversity among genes encoding *T. parva* CD8^+^ T cell antigens from buffalo-derived parasites. Sequence diversity is a prominent feature of the two previously studied *T. parva* antigens, Tp1 and Tp2, particularly in buffalo-derived *T. parva* (Pelle *et al.* 2011). Antigenic diversity, or lack thereof, can be important in parasite immune evasion, and, for *T. parva*, previous studies have implicated epitope sequence variation in evasion from the CTL response (MacHugh *et al.* 2009; Connelley *et al.* 2011). Of practical significance, widespread sequence diversity among functional CD8^+^ T cell antigens would greatly increase the challenge of designing a broadly protective subunit vaccine against the parasite. The overall conclusion derived from the data reported here is that, although there was substantial diversity at the nucleotide level, extensive diversity in the amino acid sequences is not a feature of all antigens. In particular, no polymorphism was observed in the CD8+ T cell epitope sequences in four antigens (Tp4, 5, 7 and 8) for which the epitopes have been defined, compared with the marked variation in epitope sequences observed in Tp2 and Tp9 and to a lesser extent in Tp1.

There was a considerable difference in the degree of diversity observed in the antigen sequences, as summarized in Tables 5 and 6. The findings on diversity were relatively consistent based on ratios obtained for the following parameters: (1) the number of nucleotide variants/number of sequences obtained, (2) the number of protein variants/number of sequences obtained, (3) the number of protein variants/number of nucleotide variants, and (4) the number of non-synonymous SNP locations/total number of SNP locations. Tp1, Tp2 and Tp9 were consistently the most diverse (the highest ratios) whereas Tp4, Tp5, Tp6 and Tp7 were consistently the least diverse (the lowest ratios). No diversity was observed in the epitope sequences of the Tp4, Tp5, Tp7 and Tp8 antigens, suggesting that extensive diversity is not a universal feature of *T. parva* CTL antigens. However, the data need to be interpreted with some caution, given that the sequences which were analysed represent different proportions of the coding region of each gene. We also detected very few novel indels and introns, suggesting that the major diversity among the antigen genes is due to SNPs and not variations in the indel or intron composition.

In comparing the different antigen genes, Tp1, Tp2 and Tp9 were the most challenging to sequence, sometimes resulting in failure to obtain a PCR product or in sequences that were obviously mixed. In contrast, sequences obtained from Tp6, Tp7 and Tp8 usually resulted in good quality sequences. A likely explanation for the former inconsistency is that these genes possess sequence diversity in the region corresponding to one or both primers. Although the primers used were initially shown to generate products from a panel of laboratory isolates of *T. parva*, it is possible that some allelic variants are not amplified. As further genome sequence data becomes available for more *T. parva* isolates, it may be possible to develop improved PCR primers. However, at least for Tp2 and Tp9, this may still be challenging in view of the extensive nucleotide differences throughout these genes. The ambiguous mixed sequences are almost certainly due to infection of the cell lines with two or more strains of the parasite, resulting in several sequences in the PCR amplicon.

It has been known for some time that cells infected with the closely related theilerial parasite, *T.* sp. *buffalo*, can be established *in vitro* from infected buffalo (Zweygarth *et al.* 2009). Recent research published after the current study has also shown that this parasite can infect cattle and that cell lines established from buffalo or cattle co-grazed with buffalo may be co-infected with *T. parva* and *T.* sp. *buffalo* (Bishop *et al.* 2015). To avoid the possibility of obtaining sequences from both *Theileria* species, we used species-specific primers to screen for the presence or absence of *T. parva* and *T.* sp. *buffalo* (Bishop *et al.* 2015) and only used cell lines that did not have a detectable infection with *T.*sp. *buffalo*

It should be noted that the sequences obtained may not be strictly representative of the parasite populations *in vivo*, as the approach of direct sequencing of PCR amplicons may generate a bias, in the case of infections with mixed genotypes, to strains that are more abundant or whose sequences are more efficiently amplified by the PCR primers employed. Additionally, maintenance of *Theileria-*infected cells in culture may select for parasite-infected cells which are more suited to *in vitro* cell propagation, thus altering the composition of the parasites originally present in the host. Hemmink *et al.* (2016) have previously shown that 454 deep sequencing (Roche) can be used to reveal alleles present at a low frequency in the parasites in the Muguga cocktail ITM vaccine. In a parallel study, we have applied the same high-throughput sequencing approach to compare the sequences of six of TpAg genes (Tp1, Tp2, Tp4, Tp5, Tp6 and Tp10) examined in the current study, in samples obtained from naturally infected buffalo in two geographically distant locations; PCR amplicons of segments of these genes generated from DNA extracted directly from buffalo blood were used for sequencing. These studies generated larger sets of sequences and consequently revealed greater diversity but overall the observed patterns of diversity were very similar to those observed here, ranging from some highly polymorphic genes showing high ratios of non-synonymous:synonymous nucleotide substitution to others showing lower diversity and low ratios of non-synonymous:synonymous nucleotide substitution, with relatively conserved amino acid sequences (Hemmink *et al.* 2017).

Our studies have identified major variation in the level of sequence diversity exhibited by the genes encoding different *T. parva* antigens recognized by parasite-specific CD8+ T cells. The extensive polymorphism seen in some antigens could be of great value for typing and differentiating populations of *T. parva*. Conversely, limited diversity is a most desirable feature of antigens that could potentially be used in a subunit vaccine against disease caused by *T. parva*. The epitopes recognized by CD8+ T cells from *T. parva*-immune cattle depend heavily on the class I MHC background of the individual animal. This adds to the complexity of the development of subunit vaccines and may well require the use of multiple antigens to consistently generate the desired immune responses.

We and others have observed that the currently used Muguga cocktail live vaccine offers limited protection to cattle introduced into pasture co-grazed predominantly by buffalo (Bishop *et al.* 2015; Sitt *et al.* 2015). Early studies (Radley *et al.* 1979; Young, 1981) providing an indication that this is associated with greater antigenic diversity among buffalo-derived parasites have been supported by a subsequent study that showed much more extensive diversity in the amino acid sequences of the Tp1 and Tp2 CD8+ T cell antigens in buffalo isolates compared from those derived from cattle (Pelle *et al.* 2011). However, the studies reported here suggest that antigenic diversity is not a universal feature of the *T. parva* antigens identified to date, but rather that immune cattle can also respond to antigens that appear to be highly conserved among *T. parva* isolates, including those from cattle.

## Supplementary material

For supplementary material accompanying this paper visit http://dx.doi.org/10.1017/S0031182018000264. click here to view supplementary material
