# Supplementary material for: Theileria parva antigens recognized by CD8+ T cells show varying degrees of diversity in buffalo-derived infected cell lines
Source: Parasitology. 2018 May 6;145(11):1430–9. doi: 10.1017/S0031182018000264 (PMC6126638; doi:10.1017/S0031182018000264)
Supplement: Supplementary file 1 [file S0031182018000264sup001.docx]

**Table S1: PCR cycling conditions**

|  | **Initiation** | **Denaturation** | **Annealing** | **Extension** | **Final Extension** | **Hold** |
| --- | --- | --- | --- | --- | --- | --- |
| **Tp1, Tp2** | 95^o^C, 11 min | 41 cycles:  95^o^C, 1 min | 50^o^C, 1 min | 72^o^C, 1 min | 72^o^C, 10 min | 10^o^C |
| **Tp3, Tp4, Tp7** | 95^o^C, 11 min | 43 cycles:  95^o^C, 30 s | 50^o^C, 1 min | 72^o^C, 1.5 min | 72^o^C, 10 min | 10^o^C |
| **Tp5** | 95^o^C, 11 min | 40 cycles:  95^o^C, 30 s | 50^o^C for 45s | 72^o^C, 30 s | 72^o^C, 10 min | 10^o^C |
| **Tp6** | 95^o^C, 11 min | 42 cycles:  95^o^C, 30 s | 55^o^C, 1 min | 72^o^C, 1.5 min | 72^o^C, 10 min | 10^o^C |
| **Tp8, Tp10** | 95^o^C, 11 min | 40 cycles:  95^o^C, 30 s | 55^o^C, 1 min | 72^o^C, 2 min | 72^o^C, 10 min | 10^o^C |
| **Tp9** | 95^o^C, 11 min | 30 cycles:  95^o^C, 30 s | 55^o^C, 1 min | 72^o^C, 1.5 min | 72^o^C, 10 min | 10^o^C |

**Table S2a: Stabilate information and accession numbers**

| Sample  ID | Stabilate  Name | Tp1 | Tp2^2^ | Tp3 | Tp4 | Tp5 | Tp6 | Tp7 | Tp8 | Tp10 |
| --- | --- | --- | --- | --- | --- | --- | --- | --- | --- | --- |
| Buffalo-derived *T. parva* | | | | | | | | | | |
| BD1 | Mara 3^1^ | *JF451995* | *JF451900* | KY556754 | KY556860 | KY556797 | KY581701 | KY556818 | KY556832 | KY556721 |
| BD2 | Mara 4 ^1^ | *JF451954* | *JF451901* | KY556755 | - | KY556798 | KY581702 | KY556821 | - | - |
| BD3 | Mara 18^1^ | *JF451996* | *JF451902* | KY556756 | KY556877 | KY556799 | KY581703 | KY556819 | - | KY556722 |
| BD4 | Mara 30 ^1^ | *JF451955* | *JF451903* | KY556758 | KY556878 | KY556781 | KY581704 | KY556803 | KY556834 | KY556723 |
| BD5 | Mara 32 ^1^ | *JF451956* | *JF451904* | KY556761 | KY556879 | KY556782 | KY581705 | KY556817 | KY556835 | KY556724 |
| BD6 | Mara 42 ^1^ | *JF451997* | *JF451905* | KY556760 | KY556882 | KY556800 | KY581706 | KY556802 | KY556838 | KY556726 |
| BD7 | 6998 ^1,2^ | *JF451957* | *JF451906* | KY556769 | KY556869 | KY556791 | KY581716 | KY556809 | KY556849 | KY556734 |
| BD8 | 6999^2^ | *JF451998* | *JF451898* | KY556771 | KY556870 | KY556792 | KY581718 | KY556811 | KY556851 | KY556736 |
| BD9 | 7001^2^ | *JF451999* | *JF451907* | KY556772 | KY556872 | KY556793 | KY581720 | KY556812 | - | KY556737 |
| BD10 | 7546^3^ | *JF452000* | *JF451908* | KY556775 | KY556875 | KY556795 | KY581723 | KY556816 | - | KY556739 |
| BD14 | 7014^2^ | *JF451958* | *JF451912* | KY556773 | - | KY556794 | KY581721 | KY556813 | KY556854 | KY556738 |
| BD15 | 7698^3^ | *JF451991* | *JF451913* | KY556776 | KY556876 | KY556796 | KY581724 | KY556829 | KY556856 | KY556740 |
| BD16 | 7344^3^ | *JF452003* | *JF451899* | KY556774 | KY556874 | - | KY581722 | KY556815 | KY556853 | - |
| BD17 | Mara 41^1^ | - | - | KY556759 | KY556881 | - | - | KY556820 | KY556837 | KY556725 |
| BD18 | 4740^4^ | KY556886 | KY556753 | KY556762 | KY556861 | - | KY581707 | KY556804 | KY556839 | KY556727 |
| BD19 | 5012^6^ | KY556887 | KY556744 | KY556763 | KY556862 | KY556784 | KY581708 | KY556801 | KY556840 | KY556728 |
| BD20 | 5489^4^ | KY556888 | KY556745 | KY556764 | KY556863 | - | KY581709 | KY556805 | KY556841 | KY556729 |
| BD21 | 6666^2^ | KY556889 | KY556746 | KY556765 | KY556864 | - | KY581711 | KY556806 | KY556843 | KY556730 |
| BD22 | 6807^2^ | KY556890 | - | - | KY556865 | KY556785 | - | KY556807 | KY556844 | KY556731 |
| BD23 | 6818^2^ | KY556891 | - | KY556766 | KY556866 | KY556786 | KY581712 | KY556808 | KY556845 | - |
| BD24 | 6819^1^ | KY556892 | KY556747 | KY556767 | KY556867 | KY556787 | KY581713 | KY556828 | KY556846 | KY556732 |
| BD25 | 6998 cl. 11^3^ | KY556893 | KY556748 | KY556770 | KY556868 | - | KY581717 | KY556810 | KY556850 | KY556735 |
| BD26 | 7065^2^ | - | - | - | KY556873 | - | KY581727 | KY556814 | KY556855 | - |
| Cattle-derived *T. parva* | | | | | | | | | | |
| CD16 | Tc867 Bt106^3^ | KY556896 | KY556752 | KY556779 | KY556885 | - | KY581726 | - | KY556859 | KY556743 |
| CD17 | Tc841 IL57^3^ | KY556897 | KY556751 | KY556778 | KY556884 | - | KY581725 | KY556831 | KY556858 | KY556742 |
| CD28 | Tc120F344^5^ | KY556895 | KY556750 | KY556777 | KY556883 | - | - | KY556830 | KY556857 | KY556741 |

Italicized accession numbers indicate previously published sequences. – indicates samples were not run or no sequence data was available. ^1^ Conrad et al., 1987, ^2^ Baldwin et al. 1988, ^3^ Pelle et al., 2011, ^4^ ILRI biorepository, ^5^ Pelle, personal communication, ^4^ Grootenhuis et al., 1987, ^6^ unknown origin.

**Table S2b: Tp9 Accession numbers**

| Sample ID | Stabilate name | Genomic DNA^1^ | Cloned DNA^1^ |
| --- | --- | --- | --- |
| Buffalo-derived *T. parva* |  |  |  |
| BD1 | Mara 3 | KY563669 | KY563670 |
| BD2 | Mara 4 | **-** | KY563671 |
| BD3 | Mara 18 | KY563673 | KY563672 |
| BD4 | Mara 30 | KY563675 | KY563674 |
| BD5 | Mara 32 | **-** | KY563676 |
| BD6 | Mara 42 | KY563678 | KY563677 |
| BD7 | 6998 | KY563682 | KY563683 |
| BD8 | 6999 | - | KY563685 |
| BD9 | 7001 | **-** | KY563686 |
| BD10 | 7546 | **-** | KY563690 |
| BD14 | 7014 | **-** | KY563687 |
| BD15 | 7698 | **-** | KY563691 |
| BD16 | 7344 | KY563689 | KY563688 |
| BD18 | 4740 | KY563679 | **-** |
| BD19 | 5012 | KY563680 | - |
| BD25 | 6998 cl. 11 | **-** | KY563684 |
| Cattle-derived *T. parva* |  |  |  |
| CD16 | Tc 867 Bt106 | - | KY563694 |
| CD17 | Tc 841 IL57 | - | KY563693 |
| CD28 | Tc 120 F344 | **-** | KY563692 |

**Table S3a: Tp1 nucleotide variant Clustal alignment**

TP03_0849(genomic) T***ATGGCCACTTCAATTGCATTTGCC***GCTGATCCTGGATTCTGTTATTTTCTATTAATACC 720

XM_757880(mRNA) TATGGCCACTTCAATTGCATTTGCCGCTGATCCTGGATTCTGTTATTTTCTATTAATACC 720

V1(3) -------------------------GCTGATCCTGGATTCTGTTATTTTCTATTAATACC 35

V2(1) -------------------------GCTGATCCTGGATTCTGTTATTTTCTATTAATACC 35

V13(2) -------------------------GCTGATCCTGGATTCTGTTATTTTCTATTAATACC 35

V36(1) -------------------------GCTGATCCTGGATTCTGTTATTTTCTATTAATACC 35

V37(1) -------------------------GCTGATCCTGGATTCTGTTATTTTCTATTAATACC 35

V38(1) -------------------------TCTGATCCTGGATTCTGTTATTTTCTATTAATACC 35

V39(1) -------------------------GCTGATCCTGGATTCTGTTATTTTCTATTAATACC 35

V40(1) -------------------------GCTGATCCTGGATTCTGTTATTTTCTATTAATACC 35

**********************************

TP03_0849(genomic) AGGCCCTGACTCGAAACCAATATTCTTCAAAAACGACGGAGATAAATTTTTACGTTGCGT 780

XM_757880(mRNA) AGGCCCTGACTCGAAACCAATATTCTTCAAAAACGACGGAGATAAATTTTTACGTTGCGT 780

V1(3) AGGCCCTGACTCGAAACCAATATTCTTCAAAAACGACGGAGATAAATTTTTACGTTGCGT 95

V2(1) AGGCCCTGACTCGAAACCCATATTCTTCAAAAACGACGGAGATAAATTTTTACGTTGCGT 95

V13(2) AGGCCCTGACTCGAAACCTATATTCTTCAAAAACGACGGAGATAAATTTTTACGTTGCGT 95

V36(1) AGGCCCTGACTCGAAACCAATATTCTTCAAAAACGACGGAGATAAATTTTTACGTTGCGT 95

V37(1) AGGCCCTGACTCGAAACCAATATTCTTCAAAAACGACGGAGATAAATTTTTACGTTGCGT 95

V38(1) AGGCCCTGACTCGAAACCTATATTCTTCAAAAACGACGGAGATAAATTTTTACGTTGCGT 95

V39(1) AGGCCCTGACTCGAAACCAATATTCCTCAAAAACGACGGAGATAAATTTTTACGTTGCGT 95

V40(1) AGGCCCTGACTCGAAACCTATATTCTTCAAAAACGACGGAGATAAATTTTTACGTTGCGT 95

****************** ****** **********************************

TP03_0849(genomic) AGGGTATCCAAAGGTTAAAGAAGAAATGCTAGAAATGGCTACAAAATTCAATAGACTACC 840

XM_757880(mRNA) AGGGTATCCAAAGGTTAAAGAAGAAATGCTAGAAATGGCTACAAAATTCAATAGACTACC 840

V1(3) AGGGTATCCAAAGGTTAAAGAAGAAATGCTAGAAATGGCTACAAAATTCAATAGACTACC 155

V2(1) AGGGTATCCAAAGGTTAAAGAAGAAATTATAGAAATGGCTACAAAATTCAATAGACTACC 155

V13(2) AGGGTATCCAAAGGTTAAAGAAGAAATGCTAGAAATGGCTACAAAATTCAATAGACTACC 155

V36(1) AGGGTATCCAAAGGTTAAAGAAGAAATGCTAGAAATGGCTACAAAATTCAATAGACTACC 155

V37(1) AGGGTATCCAAAGGTTAAAGAAGAAATGCTAGAAATGGCTACAAAATTCAATAGACTACC 155

V38(1) AGGGTATCCAAAGGTTAAAGAAGAAATGCTAGAAATGGCTACAAAATTCAATAGATTACC 155

V39(1) AGGGTATCCAAAGGTTAAAGAAGAAATGCTAGAAATGGCTACAAAATTCAATAGATTACC 155

V40(1) AGGGTATCCAAAGGTTAAAGAAGAAATGCTAGAAATGGCTACAAAATTCAATAGACTACC 155

*************************** ************************** ****

TP03_0849(genomic) AAAGGGCGTGGAAATACCTGCACCTCCAGGAGTAAAACCAGAGGCTCCCACACCTACACC 900

XM_757880(mRNA) AAAGGGCGTGGAAATACCTGCACCTCCAGGAGTAAAACCAGAGGCTCCCACACCTACACC 900

V1(3) AAAGGGCGTGGAAATACCTGCACCTCCAGGAGTAAAACCAGAGGCTCCCACACCTACACC 215

V2(1) AAAGGGCGTGGAAATACCTGCACCTCCAGGAGTAAAACCAGAGGCTCCCACACCTACACC 215

V13(2) AAAGGGCATGGAAATACCTGCACCTCCAGGAGTAAAACCAGAGGCTCCCACACCTACACC 215

V36(1) AAAGGGCATGGAAATACCTTCACCTCCAGGAGTAAAACCAGAGGCTCCCACACCTACACC 215

V37(1) AAAGGGCATGGAAATACCTTCACCTCCAGGAGTAAAACCAGAGGCTCCCACACCTACACC 215

V38(1) AAAGGGCGTGGAAATACCTGCACCTCCAGGAGTAAAACCAGAGGCTCCCACACCTACACC 215

V39(1) AAAGGGCGTGGAAATACCTGCACCTCCAGGAGTAAAACCAGAGGCTCCCACACCTACACC 215

V40(1) AAAGGGCATGGAAATACCTGCACCTCCAGGAGTAAAACCAGAGGCTCCCACACCTACACC 215

******* *********** ****************************************

TP03_0849(genomic) AACGACAATAACTCCTTCTGTACCTCCTACTATACCAACGCCAATAACTCCTTCGGCACC 960

XM_757880(mRNA) AACGACAATAACTCCTTCTGTACCTCCTACTATACCAACGCCAATAACTCCTTCGGCACC 960

V1(3) AACGACAATAACTCCTTCTGTACCTCCTACTATACCAACGCCAATAACTCCTTCGGCACC 275

V2(1) AACGACAATAACTCCTTCTGTACCTCCTACTATACCAACGCCAATAACTCCTTCGGCACC 275

V13(2) AACGACAATAACTCCTTCTGTACCTCCTACTATACCAACTCCAATAACTCCTTCTGCACC 275

V36(1) AACGACAATAACTCCCTCTGTACCTCCTACTATACCAACTCCAATAACTCCTTCGGCACC 275

V37(1) AACGACAATAACTCCCTCTGTACCTCCTACTATACCAACTCCAATAACTCCTTCGGCACC 275

V38(1) AACGACAATAACTCCTTCTGTACCTCCTACTATACCAACGCCAATAACTCCTTCGGCACC 275

V39(1) AACTCCAATA------------------------------------ACTCCTTCTGCACC 239

V40(1) AACGACAATAACTCCCTCTGTACCTCCTACTATACCAACTCCAATAACTCCTTCTGCACC 273

*** ***** ******** ***

TP03_0849(genomic) TCCTA------------CTACACCACCTACGGGACTAAATTTTAACTTGACAGTTCAGAA 1008

XM_757880(mRNA) TCCTA------------CTACACCACCTACGGGACTAAATTTTAACTTGACAGTTCAGAA 1008

V1(3) TCCTA------------CTACACCACCTACGGGACTAAATTTTAACTTGACAGTTCAGAA 323

V2(1) TCCTA------------CTACACCACCTACGGGACTAAATTTTAACTTGACAGTTCAGAA 323

V13(2) TCCTTCTGCACCTCCTACTACACCACCTAAGGGACTAAATTTTAACTTGACACTTCAGAA 335

V36(1) TCCTACTACACCACCTACTACACCACCTACGGGACTAAATTTTAACTTGACAGTTCAGAA 335

V37(1) TCCTACTACACCTCCTACTACACCACCTAAGGGACTAAATTTTAACTTGACAGTTCAGAA 335

V38(1) TCCTA------------CTACACCAACTACGGGACTAAATTTTAACTTGACACGTCAGAA 323

V39(1) TCCTACTACACCTCCTACTACACCACCTAAGGGACTAAATTTTAACTTGACAGTTCAGAA 299

V40(1) TCCTA------------CTACACCACCTACGGGACTAAATTTTAACTTGACACTTCAGAA 323

******** *** ********************** ******

TP03_0849(genomic) CAAATTCATGATAGGTTCGCAAGAAGTTAAGTTAAATATAACTCACGAATACGAGGGTGT 1068

XM_757880(mRNA) CAAATTCATGATAGGTTCGCAAGAAGTTAAGTTAAATATAACTCACGAATACGAGGGTGT 1068

V1(3) CAAATTCATGATAGGTTCGCAAGAAGTTAAGTTAAATATAACTCACGAATACGAGGGTGT 383

V2(1) CAAATTCATGGTAGGTTCGCAAGAAGTTAAGTTAAATATAACTCACGAATACGATGGTGT 383

V13(2) CAAATTCATGATAGGTTCGCAAGAAGTTAAGTTAAGTATAACTCACGAATACGATGGTGT 395

V36(1) CAAATTCATGGTAGGTTCGCAAGAAGTTAAGTTAAGTATAACTTACGAATACGATGGTGT 395

V37(1) CAAATTCATGGTAGGCTCGCAAGAAGTTAAGTTAAGTATAACTTACGAATACGATGGTGT 395

V38(1) CAAATTCATGATAGGTTCGCAAGAAGTTAAGTTAAATATAACTTACGAATACGATGGTGT 383

V39(1) CAAATTCATGGTAGGTTCGCAAGAAGTTAAGTTAAATATAACTCACGAATACGAGGGTGT 359

V40(1) CAAATTCATGATAGGTTCGCAAGAAGTTAAGTTAAGTATAACTCACGAATACGATGGTGT 383

********** **** ******************* ******* ********** *****

TP03_0849(genomic) ATAC***GAAGCTCATAAATATTTCATT***GAAAGGGGCAGCTTTACCCCTACCTCATTCTCAAT 1128

XM_757880(mRNA) ATACGAAGCTCATAAATATTTCATTGAAAGGGGCAGCTTTACCCCTACCTCATTCTCAAT 1128

V1(3) ATAC-------------------------------------------------------- 387

V2(1) ATAC-------------------------------------------------------- 387

V13(2) ATAC-------------------------------------------------------- 399

V36(1) ATAC-------------------------------------------------------- 399

V37(1) ATAC-------------------------------------------------------- 399

V38(1) ATAC-------------------------------------------------------- 387

V39(1) ATAC-------------------------------------------------------- 363

V40(1) ATAC-------------------------------------------------------- 387

****

Primer regions are in bold, underlined and italicized text.

**Table S3b: Tp1 protein variant Clustal alignment**

XP_762973 FAADPGFCYFLLIPGPDSKPIFFKNDGDKFLRCVGYPKVKEEMLEMATKFNRLPKGVEIP 240

V1(3) --ADPGFCYFLLIPGPDSKPIFFKNDGDKFLRCVGYPKVKEEMLEMATKFNRLPKGVEIP 58

V2(1) --ADPGFCYFLLIPGPDSKPIFFKNDGDKFLRCVGYPKVKEEIIEMATKFNRLPKGVEIP 58

V11(2) --ADPGFCYFLLIPGPDSKPIFFKNDGDKFLRCVGYPKVKEEMLEMATKFNRLPKGMEIP 58

V13(1) --ADPGFCYFLLIPGPDSKPIFFKNDGDKFLRCVGYPKVKEEMLEMATKFNRLPKGMEIP 58

V17(1) --ADPGFCYFLLIPGPDSKPIFLKNDGDKFLRCVGYPKVKEEMLEMATKFNRLPKGVEIP 58

V35(1) --ADPGFCYFLLIPGPDSKPIFFKNDGDKFLRCVGYPKVKEEMLEMATKFNRLPKGMEIP 58

V36(1) --ADPGFCYFLLIPGPDSKPIFFKNDGDKFLRCVGYPKVKEEMLEMATKFNRLPKGMEIP 58

V37(1) --SDPGFCYFLLIPGPDSKPIFFKNDGDKFLRCVGYPKVKEEMLEMATKFNRLPKGVEIP 58

:*******************:*******************::************:***

XP_762973 APPGVKPEAPTPTPTTITPSVPPTIPTPITPSAPPT----TPPTGLNFNLTVQNKFMIGS 296

V1(3) APPGVKPEAPTPTPTTITPSVPPTIPTPITPSAPPT----TPPTGLNFNLTVQNKFMIGS 114

V2(1) APPGVKPEAPTPTPTTITPSVPPTIPTPITPSAPPT----TPPTGLNFNLTVQNKFMVGS 114

V11(2) APPGVKPEAPTPTPTTITPSVPPTIPTPITPSAPPSAPPTTPPKGLNFNLTLQNKFMIGS 118

V13(1) SPPGVKPEAPTPTPTTITPSVPPTIPTPITPSAPPTTPPTTPPTGLNFNLTVQNKFMVGS 118

V17(1) APPGVKPEAPTPTPT------------PITPSAPPTTPPTTPPKGLNFNLTVQNKFMVGS 106

V35(1) SPPGVKPEAPTPTPTTITPSVPPTIPTPITPSAPPTTPPTTPPKGLNFNLTVQNKFMVGS 118

V36(1) APPGVKPEAPTPTPTTITPSVPPTIPTPITPSAPPT----TPPTGLNFNLTLQNKFMIGS 114

V37(1) APPGVKPEAPTPTPTTITPSVPPTIPTPITPSAPPT----TPTTGLNFNLTRQNKFMIGS 114

:************** ********: * .******* *****:**

XP_762973 QEVKLNITHEYEGVYEAHKYFIERGSFTPTSFSIGDLPQTGLPVNQTVDTIVVYFHRVTM 356

V1(3) QEVKLNITHEYEGVY--------------------------------------------- 129

V2(1) QEVKLNITHEYDGVY--------------------------------------------- 129

V11(2) QEVKLSITHEYDGVY--------------------------------------------- 133

V13(1) QEVKLSITYEYDGVY--------------------------------------------- 133

V17(1) QEVKLNITHEYEGVY--------------------------------------------- 121

V35(1) QEVKLSITYEYDGVY--------------------------------------------- 133

V36(1) QEVKLSITHEYDGVY--------------------------------------------- 129

V37(1) QEVKLNITYEYDGVY--------------------------------------------- 129

*****.**:**:***

Eptiope region highlighted in grey.

**Table S4a: Tp2 nucleotide variant Clustal alignment**

TP01_0056(genomic) ATAAAAAG***ATGAAATTGGCCGCCAGATTA***ATTAGCCTTTACTTTATTATTTACATTTTAC 180

XM_765583(mRNA) ATAAAAAGATGAAATTGGCCGCCAGATTAATTAGCCTTTACTTTATTATTTACATTTTAC 180

V1(2) -----------------------------ATTAGCCTTTACTTTATTATTTACATTTTAC 31

V44(1) -----------------------------ATTAGTCTTTACTTTGTTATTTTCATTCTAC 31

V45(1) -----------------------------ATTAGCCTTTACTTTATTATTTTCATTCTAC 31

V46(1) -----------------------------ATTAGCCTTTACTTTATTATTTTCATTCTAC 31

V47(1) -----------------------------ATTAGCCTTTACTTTATTATTTTCATTCTAC 31

V48(2) -----------------------------ATTAGCCTTTACTTTATTATTTTCATTCTAC 31

V49(1) -----------------------------ATTAGCCTTTACTTTATTATTTTCATTCTAC 31

***** *********.******:**** ***

TP01_0056(genomic) ATTCCCCAGTGCTGGGAGGTAATTGT---AGTCATGAAGAACTAAAAAAATTGGGAATGC 237

XM_765583(mRNA) ATTCCCCAGTGCTGGGAGGTAATTGT---AGTCATGAAGAACTAAAAAAATTGGGAATGC 237

V1(2) ATTCCCCAGTGCTGGGAGGTAATTGT---AGTCATGAAGAACTAAAAAAATTGGGAATGC 88

V44(1) CTTCCTCAGTCTTGGGAGGTAATTGT---ACTAATGAAGAACTAAAAAAATTGGGAATGG 88

V45(1) CTTCCTCAGTCTTGGGAAATAATAATTGTAGTGATAGTGAGCTAGAAACTTTGGGTATGT 91

V46(1) CTTCCTCAGTCTTGGGAGGTAATTGT---ACTGAGGAAGAACTAAAAAAAATGGGAATGG 88

V47(1) CTTCCTCAGTCTTGGGAGGTAATTGT---AGTGACAATGAGCTGGATACTTTGGGTTTGT 88

V48(2) CTTCCTCAGTCTTGGGAGGTAATTGT---AGTGACAATGAGCTGGATACTTTGGGTTTGT 88

V49(1) CTTCCTCAGTCTTGGGAGGTAATTGT---AGTGATGATGAACTAAGAAAATTGGGAATGT 88

.**** **** *****. **** .* * * * .. **.**. .:*.::****::**

TP01_0056(genomic) TAGAGGGCGATGGTTTCGACAGGGATGCATTGTTCAAATCATCACATGGTATGGGAAAGG 297

XM_765583(mRNA) TAGAGGGCGATGGTTTCGACAGGGATGCATTGTTCAAATCATCACATGGTATGGGAAAGG 297

V1(2) TAGAGGGCGATGGTTTCGACAGGGATGCATTGTTCAAATCATCACATGGTATGGGAAAGG 148

V44(1) TAGTGGGTGAAGGTTTAGATATGGAGGCATTGTTCAAAACATCTAAGGGTATGACGAAAG 148

V45(1) TGGACAAACCGGATCCTGACAAGCAAAGATTGTTCTTAACATCTAAGGCTATGTCGACAG 151

V46(1) TAGAGGGTGAAGGTTTCGATAAGGAAAAATTGTTCAAATCATCAAAGAGTATGGGAATAG 148

V47(1) TGGACAAACCAGATCTTGACAAGAATAGATTGTTTTTAACATCACATGGAATGGGAAAGA 148

V48(2) TGGACAAACCAGATCTTGACAAGAATAGATTGTTCTTAACATCACATGGAATGGGAAAGA 148

V49(1) TAAAGGGCGATGGTTTCGACAGGGATGCATTGTTCAAATCATCACATGGAATGGGAAAGG 148

*..: .. . *.* ** * * * . ****** : . ****:.* . :*** .* ..

TP01_0056(genomic) TAGGAAAAAGGTATGGTCTTAAAACTACTCCAAAAGTAGATAAAGTCTTAGCAGATCTTG 357

XM_765583(mRNA) TAGGAAAAAGGTATGGTCTTAAAACTACTCCAAAAGTAGATAAAGTCTTAGCAGATCTTG 357

V1(2) TAGGAAAAAGGTATGGTCTTAAAACTACTCCAAAAGTAGATAAAGTCTTAGCAGATCTTG 208

V44(1) TAGGGAGAAAGTATGGTATTAGACCTGGAACTACAAAAGATAAACTTACTAAAGAACTTA 208

V45(1) TAGGGAAAAAATATGGTATTAGACCTGGAACAAAAACAGAAAAATTTCTAAAAGAACTTA 211

V46(1) TAGGGAGAAATCATGGTCTTAAACCAAAACCAAGATTAGAGAGTGTATTTGAAGATCTTG 208

V47(1) TAGCTAGAAGGTTTGGTATTAGACCTGGAACAAAAACAGAAAAATTCTTAAAAGAACTTA 208

V48(2) TAGGTAGAAGGTTTGGTATTAGACCTGGAACAAAAACAGAAAAATTCTTAAAAGAACTTA 208

V49(1) TAGGTAGAATGCATGGTCTTAAAACGACTCCAAAAGTAGAGAGAGTCTTAGAAGATCTTG 208

*** *.** :**** *:*.*.* . :.*:* * *** ..: * :..:**:.**

TP01_0056(genomic) AAACACTGTTTGGAAAACACGGTCTTGGTGGTATTAGTAAAGATTGTCTTAAATGTTTTG 417

XM_765583(mRNA) AAACACTGTTTGGAAAACACGGTCTTGGTGGTATTAGTAAAGATTGTCTTAAATGTTTTG 417

V1(2) AAACACTGTTTGGAAAACACGGTCTTGGTGGTATTAGTAAAGATTGTCTTAAATGTTTTG 268

V44(1) CTAGACTTCTTGAAAAAATCGGTATTATAGGAGTTAGTGAGTCTTGTCTTAGTTGTTTTG 268

V45(1) CTACTCTTCTTACACAACTAGGTATTACAGGAATTAGAGAACAATGTCTTGCCTGTTTTG 271

V46(1) AAAAACTGTTTGGAAAACACGGTCTTGGTGGTATTAGTAAAAATTGTCTTACTTGTTTTG 268

V47(1) CTAAATTATTTACAGAAATTGGTATTACAGGTGTTGGTGAGAAGTGCCTCGAATGTTTAG 268

V48(2) CTAAATTATTTACAGAAATTGGTATTACAGGTGTTGGTGAGAAGTGCCTCGAATGTTTAG 268

V49(1) AAACACTGTTTGGAAAAATCGGTCTTGGTGGTATTAGTAAAGATTGTCTTAAATGTTTTG 268

* : * **. * ** : ***.**. :** .* .*:... . ** ** *****:*

TP01_0056(genomic) CACAAAGCCTAGTGTGCGTATTAATGAAATGTAGAGGAGCATGTCTCAAAGGACCATGTA 477

XM_765583(mRNA) CACAAAGCCTAGTGTGCGTATTAATGAAATGTAGAGGAGCATGTCTCAAAGGACCATGTA 477

V1(2) CACAAAGCCTAGTGTGCGTATTAATGAAATGTAGAGGAGCATGTCTCAAAGGACCATGTA 328

V44(1) GACAAAGCATTAAGTGTGTAGCACAACATTGCAAGGGAGCGTGTCTTAGGGGACCATGTA 328

V45(1) CACAAAGCATATATTGCGTTGCGAATAATTGCAGGGGAGCATGTCTTAGGGGTCCATGTA 331

V46(1) TGCAAAGCATTATGTGTGTTATAAATAAATGTAGAGGAGCATGTCTCAAAGGCCCATGTA 328

V47(1) CAGCAAGCATTAAGTGTGTATCACACCATTGCAAGGGAGCGTGTCTAAAGGGACCATGTA 328

V48(2) CAGCAAGCATTAAGTGTGTATCACAACATTGCAAGGGAGCGTGTCTAAAGGGACCATGTA 328

V49(1) TGGAAAGCATTATGTGTGTTATAAAAAAATGTAGAACAGCATGTCTCAGAGGACCATGTA 328

. .****.* : ** .*: ..: .* ** *... ***.***** ...*. *******

TP01_0056(genomic) CTGACGACTGCCAAAATTGCTTTGATAGAAACTGTAAATCTGCATTGCTGGAATGCATTG 537

XM_765583(mRNA) CTGACGACTGCCAAAATTGCTTTGATAGAAACTGTAAATCTGCATTGCTGGAATGCATTG 537

V1(2) CTGACGACTGCCAAAATTGCTTTGATAGAAACTGTAAATCTGCATTGCTGGAATGCATTG 388

V44(1) CAGAAGACTGCCAAAAATGCATTGAAAAAAATTGTAAACAGGCATTGCTGGAATGTATTG 388

V45(1) CTAAAGAGTGTCAAGAATGCATTAAAAAAAATTGTAAACAGGCATTGCTGGAATGTATTG 391

V46(1) CCGACGGATGTCAAAAGTGTATTAATACTAATTGTAAACCGGCACTGCTGGAATGCATTG 388

V47(1) CAGAAGGCTGTCAAGAATGCATTAAAAGAAATTGTATGGAAGCATTGCTGCAATGCATTG 388

V48(2) CAGATGGCTGTCAAGAATGCATTAAAAGAAATTGTATGGAAGCATTGCTGGAATGCATTG 388

V49(1) CTGATGACTGCCAAAATTGCATTAAAAGAAATTGTAAGCAAGGATTGCTGGAATGCATTG 388

* .* *. ** ***.* ** :**.*:* :** ***.:. . * * ***** *.** ****

TP01_0056(genomic) GGAAAACAAGTATTCCAAATCCATGTAAATGGAAAGAAGATTATCTAAAATACAAATTTC 597

XM_765583(mRNA) GGAAAACAAGTATTCCAAATCCATGTAAATGGAAAGAAGATTATCTAAAATACAAATTTC 597

V1(2) GGAAAACAAGTATTCCAAATCCATGTAAATGGAAAGAAGATTATCTAAAATACAAATTTC 448

V44(1) GAAAAGGTGATGTTCCGAATCCTTGCGATTGGGAGAAAGAATATCTAAGTTACAAACTTC 448

V45(1) GAAAAGGTGATGTTCCGAATCCTTGTCAATGGAAAGATGATTATCTAAAATTCAAACTTC 451

V46(1) GAGTAAATGATATTCCAAATCCATGTAAATGGAAAGAAGATTATCTAAAATACAAACTTC 448

V47(1) GGAAACCAAGTGTTCCAAATCCTTGTGATTGGAAAGATGATTATCTAAAATTCAAATTTC 448

V48(2) GGAAACCAAGTATTCCAAATCCTTGTGATTGGAAAGATGATTATCTAAAATTCAAATTTC 448

V49(1) GAAAAGAGGATGTTCCAAATCCTTGCGATTGGGACAAAGAATATCTAAAATACAAACTTC 448

*..:* ..:.**...*****:** *:***.* .*:** *******.:*:**** ***

TP01_0056(genomic) CTGAAACAGATGAGGACGAATCTACGAAAAAAGGA***GAAGCCTCCGGCACTTCATAG***GGAT 657

XM_765583(mRNA) CTGAAACAGATGAGGACGAATCTACGAAAAAAGGAGAAGCCTCCGGCACTTCATAGGGAT 657

V1(2) CTGAAACAGATGAGGACGAATCTACGAAAAAA---------------------------- 480

V44(1) CTGAAACA---------------------------------------------------- 456

V45(1) CTGAAACA---------------------------------------------------- 459

V46(1) CTGAAACAGATGAGGAC------------------------------------------- 465

V47(1) CTGAAACAGGTGAGGATGAGGCTCA----------------------------------- 473

V48(2) CTGAAACAGATAGTGACGAATCTGAGAAAAA----------------------------- 479

V49(1) CTGATACAGATGGGGATGAGTCTCA----------------------------------- 473

**** ***

Primer regions are in bold, underlined and italicized text.

**Table S4b: Tp2 protein variant Clustal alignment**

XP_765583 MKLAARLISLYFIIYILHSPVLGG-NCSHEELKKLGMLEGDGFDRDALFKSSHGMGKVGK 59

V1(2) -------ISLYFIIYILHSPVLGG-NCSHEELKKLGMLEGDGFDRDALFKSSHGMGKVGK 52

V42(1) -------ISLYFIIFILPSSVLGNNNCSDSELETLGMLDKPDPDKQRLFLTSKAMSTVGK 53

V43(1) -------ISLYFVIFILPSSVLGG-NCTNEELKKLGMVVGEGLDMEALFKTSKGMTKVGR 52

V44(1) -------ISLYFIIFILPSSVLGG-NCTEEELKKMGMVEGEGFDKEKLFKSSKSMGIVGR 52

V45(1) -------ISLYFIIFILPSSVLGG-NCSDDELRKLGMLKGDGFDRDALFKSSHGMGKVGR 52

V46(1) -------ISLYFIIFILPSSVLGG-NCSDNELDTLGLLDKPDLDKNRLFLTSHGMGKIAR 52

V47(2) -------ISLYFIIFILPSSVLGG-NCSDNELDTLGLLDKPDLDKNRLFLTSHGMGKIGR 52

***** *:** * *** **:..** .:*:: . * : ** :*:.* :.:

XP_765583 RYGLKTTPKVDKVLADLETLFGKHGLGGISKDCLKCFAQSLVCVLMKCRGACLKGPCTDD 119

V1(2) RYGLKTTPKVDKVLADLETLFGKHGLGGISKDCLKCFAQSLVCVLMKCRGACLKGPCTDD 112

V42(1) KYGIRPGTKTEKFLKELTTLLTQLGITGIREQCLACFAQSIYCVANNCRGACLRGPCTKE 113

V43(1) KYGIRPGTTKDKLTKELTRLLEKIGIIGVSESCLSCFGQSIKCVAQHCKGACLRGPCTED 112

V44(1) NHGLKPKPRLESVFEDLEKLFGKHGLGGISKNCLTCFVQSIMCVINKCRGACLKGPCTDG 112

V45(1) MHGLKTTPKVERVLEDLETLFGKIGLGGISKDCLKCFVESIMCVIKKCRTACLRGPCTDD 112

V46(1) RFGIRPGTKTEKFLKELTKLFTEIGITGVGEKCLECLAASIKCVSHHCKGACLKGPCTEG 112

V47(2) RFGIRPGTKTEKFLKELTKLFTEIGITGVGEKCLECLAASIKCVSQHCKGACLKGPCTDG 112

.*:: : . :* *: : *: *: .** *: *: ** :*: *** ***:.

XP_765583 CQNCFDRNCKSALLECIGKTSIPNPCKWKEDYLKYKFPETDEDESTKKGEASGTS 174

V1(2) CQNCFDRNCKSALLECIGKTSIPNPCKWKEDYLKYKFPETDEDESTKK------- 160

V42(1) CQECIKKNCKQALLECIGKGDVPNPCQWKDDYLKFKLPET--------------- 153

V43(1) CQKCIEKNCKQALLECIGKGDVPNPCDWEKEYLSYKLPET--------------- 152

V44(1) CQKCINTNCKPALLECIGVNDIPNPCKWKEDYLKYKLPETDED------------ 155

V45(1) CQNCIKRNCKQGLLECIGKEDVPNPCDWDKEYLKYKLPDTDGDES---------- 157

V46(1) CQECIKRNCMEALLQCIGKPSVPNPCDWKDDYLKFKFPETGEDEA---------- 157

V47(2) CQECIKRNCMEALLECIGKPSIPNPCDWKDDYLKFKFPETDSDESEK-------- 159

**:*:. ** .**:*** .:****.* :**.:* * *

Eptiope region highlighted in grey. Eptiopes in close proximity or overlapping are denoted by underlined regions or highlighted in a different shade of grey.

**Table S5a: Tp3 nucleotide variant Clustal alignment**

TP01_0868(genomic) ***ATGAAATTAAATACTATCGCAATAG***CCTTTTTGTATTCCTGTTTCTCACAGTTTTTAAAA 60

XM_7663892(mRNA) ATGAAATTAAATACTATCGCAATAGCCTTTTTGTATTCCTGTTTCTCACAGTTTTTAAAA 60

V1(2) ------------------------------------------------------------ 60

V2(1) ------------------------------------------------------------ 0

V3(3) ------------------------------------------------------------ 0

V4(1) ------------------------------------------------------------ 0

V5(3) ------------------------------------------------------------ 0

V6(3) ------------------------------------------------------------ 0

V7(1) ------------------------------------------------------------ 0

V8(1) ------------------------------------------------------------ 0

V9(1) ------------------------------------------------------------ 0

V10(5) ------------------------------------------------------------ 0

V11(1) ------------------------------------------------------------ 0

V12(1) ------------------------------------------------------------ 0

V13(1)* ------------------------------------------------------------ 0

TP01_0868(genomic) AATGTGTCTGCTCTGAGGCGTAGTTCTCCAGATTTGTCACCAGATGGTTCTTTTCTTCAA 120

XM_7663892(mRNA) AATGTGTCTGCTCTGAGGCGTAGTTCTCCAGATTTGTCACCAGATGGTTCTTTTCTTCAA 120

V1(2) --TGTGTCTGCTCTGAGGCGTAGTTCTCCAGATTTGTCACCAGATGGTTCTTTTCTTCAA 120

V2(1) --TGTGTCTGCTCTGAGGCGTAGTTCTCCAGATTTATCACCAGATGGTTCTTTTCTTCAA 58

V3(3) --TGTGTCTGCTCTGAGGCGTAGTTCTCCAGATTTATCACCAGATGGTTCTTTTCTTCAA 58

V4(1) --TGTGTCTGCTCTGAGGCGTAGTTCTCCAGATTTATCACCAGATGGTTCTTTTCTTCAA 58

V5(4) --TGTGTCTGCTCTGAGGCGTAGTTCTCCAGATTTATCACCAGATGGTTCTTTTCTTCAA 58

V6(3) --TGTGTCTGCTCTGAGGCGTAGTTCTCCAGATTTATCACCAGATGGTTCTTTTCTTCAA 58

V7(1) --TGTGTCTGCTCTGAGGCGTAGTTCTCCAGATTTGTCACCAGATGGTTCTTTTCTTCAA 58

V8(1) --TGTGTCTGCTCTGAGGCGTAGTTCTCCAGATTTGTCACCAGATGGTTCTTTTCTTCAA 58

V9(1) --TGTGTCTGCTCTGAGGCGTAGTTCTCCAGATTTGTCACCAGATGGTTCTTTTCTTCAA 58

V10(5) --TGTGTCTGCTCTGAGGCGTAGTTCTCCAGATTTGTCACCAGATGGTTCTTTTCTTCAA 58

V11(1) --TGTGTCTGCTCTGAGGCGTAGTTCTCCAGATTTGTCACCAGATGGTTCTTTTCTTCAA 58

V12(1) --TGTGTCTGCTCTGAGGCGTAGTTCTCCAGATTTGTCACCAGATGGTTCTTTTCTTCAA 58

V13(1)* --TGTGTCTGCTCTGAGGCGTAGTTCTCCAGATTTGTCACCAGATGGTTCTTTTCTTCAA 58

********************************* ************************

TP01_0868(genomic) GTAAAATCAGCTTCTCCTCAAGATAAACAAGATGTAATCCAAAGTTCCTCTCCTAAGGTA 180

XM_7663892(mRNA) GTAAAATCAGCTTCTCCTCAAGATAAACAAGATGTAATCCAAAGTTCCTCTCCTAAGGTA 180

V1(2) GTAAAATCAGCTTCTCCTCAAGATAAACAAGATGTAATCCAAAGTTCCTCTCCTAAGGTA 180

V2(1) GTAAAATCAGCTTCTCCTCAAGATAAACAAGATGTAATCCAAAGTTCCTCTCCTAAGGTA 118

V3(3) GTAAAATCAGCTTCTCCTCAAGATAAACAAGATGTAATCCAAAGTTCCTCTCCTAAGGTA 118

V4(1) GTAAAATCAGCTTCTCCTCAAGATAAACAAGATGTAATCCAAAGTTCCTCTGCTAAGGTA 118

V5(4) GTAAAATCAGCTTCTCCTCAAGATAAACAAGATGTAATCCAAAGTTCCTCTGCTAAGGTA 118

V6(3) GTAAAATCAGCTTCTCCTCAAGATAAACAAGATGTAATCCAAAGTTCCTCTGCTAAGGTA 118

V7(1) GTAAAATCAGCTTCTCCTCAAGATAAACAAGATGTAATCCAAAGTTCCTCTCCTAAGGTA 118

V8(1) GTAAAATCAGCTTCTCCTCAAGATAAACAAGATGTAATCCAAAGTTCCTCTCCTAAGGTA 118

V9(1) GTAAAATCAGCTTCTCCTCAAGATAAACAAGATGTAATCCAAAGTTCCTCTCCTAAGGTA 118

V10(5) GTAAAATCAGCTTCTCCTCAAGATAAACAAGATGTAATCCAAAGTTCCTCTCCTAAGGTA 118

V11(1) GTAAAATCAGCTTCTCCTCAAGATAAACAAGATGTAATCCAAAGTTCCTCTCCTAAGGTA 118

V12(1) GTAAAATCAGCTTCTCCTCAAGATAAACAAGATGTAATCCAAAGTTCCTCTCCTAAGGTA 118

V13(1)* GTAAAATCAGCTTCTCCTCAAGATAAACAAGATGTAATCCAAAGTTCCTCTCCTAAGGTA 118

*************************************************** ********

TP01_0868(genomic) ACAGTGCCTACGGTTGACCCTGAAGGCCTCAAGAAGGCGGTTACTGCAGCAGTTCTATCA 240

XM_7663892(mRNA) ACAGTGCCTACGGTTGACCCTGAAGGCCTCAAGAAGGCGGTTACTGCAGCAGTTCTATCA 240

V1(2) ACAGTGCCTACGGTTGACCCTGAAGGCCTCAAGAAGGCGGTTACTGCAGCAGTTCTATCA 240

V2(1) ACAGTGCCTGCGGTGGACCCTGAAGGCCTCAAGAAGGCGGTTACTGCAGCAGTTCTATCA 178

V3(3) ACAGTGCCTACGGTTGACCCTGAAGGCCTCAAGAAGGCGGTTACTGCAGCAGTTCTATCA 178

V4(1) ACAGTGCCTGCGGTGGACCCTGAAGGCCTCAAGAAGGCGGTTACTGCACCAGTTCTATCA 178

V5(4) ACAGTGCCTGCGGTGGACCCTGAAGGCCTCAAGAAGGCGGTTACTGCACCAGTTCTATCA 178

V6(3) ACAGTGCCTGCGGTGGACCCTGAAGGCCTCAAGAAGGCGGTTACTGCAGCAGTTCTATCA 178

V7(1) ACAGTGCCTGCGGTGGACCCTGAAGGCCTCAAGAAGGCGGTTACTGCAGCAGTTCTATCA 178

V8(1) ACAGTGCCTACGGTTGACCCTGAAGGCCTCAAGAAGGCGGTTACTGCAGCAGTTCTTTCA 178

V9(1) ACAGTGCCTACGGTTGACCCTGAAGGCCTCAAGAAGGCGGTTACTGCAGCAGTTCTATCA 178

V10(5) ACAGTGCCTACGGTTGACCCTGAAGGCCTCAAGAAGGCGGTTACTGCAGCAGTTCTATCA 178

V11(1) ACAGTGCCTACGGTTGACCCTGAAGGCCTCAAGAAGGCGGTTACTGCAGCAGTTCTATCA 178

V12(1) ACAGTGCCTACGGTTGACCCTGAAGGCCTCAAGAAGGCGGTTACTGCAGCAGTTCTATCA 178

V13(1)* ACAGTGCCTACGGTTGACCCTGAAGGCCTCAAGAAGGCGGTTACTGCAGCAGTTCTATCA 178

********* **** ********************************* ***********

TP01_0868(genomic) AACCAAAATCAAGCTCTACAAAACGGTGCTCTTAATCCAGCAGATTTCACTCAAGCTGCC 300

XM_7663892(mRNA) AACCAAAATCAAGCTCTACAAAACGGTGCTCTTAATCCAGCAGATTTCACTCAAGCTGCC 300

V1(2) AACCAAAATCAAGCTCTACAAAACGGTGCTCTTAATCCAGCAGATTTCACTCAAGCTGCC 300

V2(1) AACCAAAATCAAGCTCTACAAAACGGTGCTCTTAATCCAGCAGATTTCACTCAAGCTGCG 238

V3(3) AACCAAAATCAAGCTCTACAAAACGGTGCTCTTAATCCAGCAGATTTCACTCAAGCTGCG 238

V4(1) AACCAAAATCAAGCTCTACAAAACGGTGCTCTTAATCCAGCAGATTTCACTCAAGCCGCC 238

V5(4) AACCAAAATCAAGCTCTACAAAACGGTGCTCTTAATCCAGCAGATTTCACTCAAGCCGCC 238

V6(3) AACCAAAATCAAGCTCTACAAAACGGTGCTCTTAATCCAGCAGATTTCACTCAAGCCGCC 238

V7(1) AACCAAAATCAAGCTCTACAAAACGGTGCTCTTAATCCAGCAGATTTCACTCAAGCTGCC 238

V8(1) AACCAAAATCAAGCTCTACAAAACGGTGCTCTTAATCCAGCAGATTTCACTCAAGCTGCC 238

V9(1) AACCAAAATCAAGCTCTACAAAACGGTGCTCTTAATCCAGCAGATTTCACTCAAGCTACC 238

V10(5) AACCAAAATCAAGCTCTACAAAACGGTGCTCTTAATCCAGCAGATTTCACTCAAGCTGCC 238

V11(1) AACCAAAATCAAGCTCTACAAAACGGTGCTCTTAATCCAGCAGATTTCACTCAAGCTGCC 238

V12(1) AACCAAAATCAAGCTCTACAAAACGGTGCTCTTAATCCAGCAGATTTCACTCAAGCTGCC 238

V13(1)* AACCAAAATCAAGCTCTACAAAACGGTGCTCTTAATCCAGCAGATTTCACTCAAGCTGCC 238

******************************************************* * *

TP01_0868(genomic) TCTGTTAATTCCAT---GAGTAATGCTGTTAGTGCCATGAACAATACTGTTGGTCCAGTA 357

XM_7663892(mRNA) TCTGTTAATTCCAT---GAGTAATGCTGTTAGTGCCATGAACAATACTGTTGGTCCAGTA 357

V1(2) TCTGTTAATTCCAT---GAGTAATGCTGTTAGTGCCATGAACAATACTGTTGGTCCAGTA 357

V2(1) GCTGTTAATTCCAT---GAGTAATGCTGTTAGTGCCATGAACAATACTGTTGGTCCAGC- 294

V3(3) GCTGTTAATTCCAT---GAGTAATGCTGTTGGTGCCATGAACAACACTGTTGGTCCAGT- 294

V4(1) GCTGTTAATTCCAT---GAGTAATGCTGTTAGTGCCATGAACAATACTGTTGGTCCAGC- 294

V5(4) GCTGTTAATTCCAT---GAGTAATGCTGTTAGTGCCATGAACAATACTGTTGGTCCAGC- 294

V6(3) GCTGTTAATTCCAT---GAGTAATGCTGTTAGTGCCATGAACAATACTGTTGGTCCAGC- 294

V7(1) GCTGTTAATTCCAT---GAGTAATGCTGTTAGTGCCATGAACAATACTGTTGGTCCAGT- 294

V8(1) TCTGTTAATTCCAT---GAGTAATGCTGTTAGTGCCATGAACAATACTGTTGGTCCAGT- 294

V9(1) TCTGTTAATTCCAT---GAGTAATGCTGTTAGTGCCATGAACAATACTGTTGGTCCAGTA 295

V10(5) TCTGTTAATTCCAT---GAGTAATGCTGTTAGTGCCATGAACAATACTGTTGGTCCAGT- 294

V11(1) TCTGTTAATTCCAT---GAGTAATGCTGTTAGTGCCATGAACAATACTGTTGGTCCAGT- 294

V12(1) TCTGTTAATTCCAT---GAGTAATGCTGTTAGTGCCATGAACAATACTGTTGGTCCAGT- 294

V13(1)* TCTGTTAATTCCAT---GAGTAATGCTGTTAGTGCCATGAACAATACTGTTGGTCCAGT- 294

************* ************* ************* *************

TP01_0868(genomic) AAAAATCCCATGGCTACTGTTGGTACTATGAACTCCTTTACTGGAATGCCTGGTGTACAG 417

XM_7663892(mRNA) AAAAATCCCATGGCTACTGTTGGTACTATGAACTCCTTTACTGGAATGCCTGGTGTACAG 417

V1(2) AAAAATCCCATGGCTACTGTTGGTACTATGAACTCCTTTACTGGAATGCCTGGTGTACAG 417

V2(1) --AAATCCCATGGCTACTGTTGGTACTATGAACTCCTTTACTGGAACGCCTGGTGTACAG 352

V3(3) --AAATCCCATGACTACTGTTGGTACTATGAACTCCTTTACTGGAACGCCTGGTGTACAG 352

V4(1) --AAATCCCATGGCTACTGTTGGTACTATGAACTCCTTTACTGGAACGCCTGGTGTACAG 352

V5(4) --AAATCCCATGGCTACTGTTGGTACTATGAACTCCTTTACTGGAACGCCTGGTGTACAG 352

V6(3) --AAATCCCATGGCTACTGTTGGTACTATGAACTCCTTTACTGGAACGCCTGGTGTACAG 352

V7(1) --AAATCCCATGGCTACTGTTGGTACTATGAACTCCTTTACTGGAACGCCTGGTGTACAG 352

V8(1) --AAATCCCATGGCTACTGTTGGTACTATGAACTCCTTTACTGGAATGCCTGGTGTACAG 352

V9(1) AAAAATCCCATGGCTACTGTTGGTACTATGAACTCCTTTACTGGAATGCCTGGTGTACAG 355

V10(5) --AAATCCCATGGCTACTGTTGGTACTATGAACTCCTTTACTGGAATGCCTGGTGTACAG 352

V11(1) --AAATCCCATGGCTACTGTTGGTACTATGAACTCCTTTACTGGAATGCCTGGTGTACAG 352

V12(1) --AAATCCCATGGCTACTGTTGGTACTATGAACTCCTTTACTGGAATGCCTGGTGTACAG 352

V13(1)* --AAATCCCATGGCTACTGTTGGTACTATGAACTCCTTTACTGGAATGCCTGGTGTACAG 352

********** ********************************* *************

TP01_0868(genomic) GATAATTTTCCTCAGACACCGCCTGTTAATGTTCAAGACACCTCTACCCAAGAGAACAGT 477

XM_7663892(mRNA) GATAATTTTCCTCAGACACCGCCTGTTAATGTTCAAGACACCTCTACCCAAGAGAACAGT 477

V1(2) GATAATTTTCCTCAGACACCGCCTGTTAATGTTCAAGACACCTCTACCCAAGAGAACAGT 477

V2(1) GATACTTTTTCTCAGACACCGCCTGTTAATGTTCAAGACACCTCTACCCAAGAGAACAGT 412

V3(3) GATAATTTTCCTCAGACACCGCCTGTTAATGTTCAAGACACCTCTACCCAAGAGAACAGT 412

V4(1) GATAATTTTCCTCAGACACCGCCTGTTAATGTTCAAGACACCTCTACTCAAGAGAACAGT 412

V5(4) GATACTTTTTCTCAGACACCGCCTGTTAATGTTCAAGACACCTCTACCCAAGAGAACAGT 412

V6(3) GATACTTTTTCTCAGACACCGCCTGTTAATGTTCAAGACACCTCTACCCAAGAGAACAGT 412

V7(1) GATACTTTTCCTCAGACACCGCCTGTTAATGTTCAAGACACCTCTACTCAAGAGAACAGT 412

V8(1) GATAATTTTCCTCAGACACCGCCTGTTAATGTTCAAGACACCTCTACCCAAGAGAACAGT 412

V9(1) GATAATTTTCCTCAGACACCGCCTGTTAATGTTCAAGACACCTCTACCCAAGAGAACAGT 415

V10(5) GATAATTTTCCTCAGACACCGCCTGTTAATGTTCAAGACACCTCTACTCAAGAGAACAGT 412

V11(1) GATAATTTTCCTCAGACACCGCCTGTTAATGTTCAAGACACCTCTACCCAAGAGAACAGT 412

V12(1) GATAATTTTCCTCAGACACCGCCTGTTAATGTTCAAGACACCTCTACCCAAGAGAACAGT 412

V13(1)* GATAATTTTCCTCAGACACCGCCTGTTAATGTTCAAGACACCTCTACTCAAGAGAACAGT 412

**** **** **************************************************

TP01_0868(genomic) CTTGACAACCTAAATCTCCTCTTAGATCCCTCGTTAGTAAAGATATCTCAAGCTGATAGT 537

XM_7663892(mRNA) CTTGACAACCTAAATCTCCTCTTAGATCCCTCGTTAGTAAAGATATCTCAAGCTGATAGT 537

V1(2) CTTGACAACCTAAATCTCCTCTTAGATCCCTCGTTAGTAAAGATATCTCAAGCTGATAGT 537

V2(1) CTTGACAACCTAAATCTCCTCTTAGATCCTTCGTTAGCAAAGATATCTCAAGCTGATAGT 472

V3(3) CTTGACAACCTAAATCTCCTCTTAGATCCTTCGTTACTAAAGATATCTCAAGCTGATAGT 472

V4(1) CTTGACAACCTAAATCTCCTCTTAGATCCCTCGTTAGTAAAGATATCTCAAGCTGATAGT 472

V5(4) CTTGACAACCTAAATCTCCTCTTAGATCCTTCGTTACTAAAGATATCTCAAGCTGATAGT 472

V6(3) CTTGACAACCTAAATCTCCTCTTAGATCCTTCGTTACTAAAGATATCTCAAGCTGATAGT 472

V7(1) CTTGACAACCTAAATCTCCTCTTAGATCCCTCGTTAGTAAAGATATCTCAAGCTGATAGT 472

V8(1) CTTGACAACCTAAATCTCCTCTTAGATCCCTCGTTAGTAAAGATATCTCAAGCTGATAGT 472

V9(1) CTTGACAACCTAAATCTCCTCTTAGATCCCTCGTTAGTAAAGATATCTCAAGCTGATAGT 475

V10(5) CTTGACAACCTAAATCTCCTCTTAGATCCCTCGTTAGTAAAGATATCTCAAGCTGATAGT 472

V11(1) CTTGACAACCTAAATCTCCTCTTAGATCCTTCGTTAGTAAAGATATCTCAAGCTGATAGT 472

V12(1) CTTGACAACCTAAATCTCCTCTTAGATCCCTCGTTAGTAAAGATATCTCAAGCTGATAGT 472

V13(1)* CTTGACAACCTAAATCTCCTCTTAGATCCCTCGTTAGTAAAGATATCTCAAGCTGATAGT 472

***************************** ****** **********************

TP01_0868(genomic) CACATAAAAGAAAGCATGGTTAGTTTCATTTTTATTAATTTGTTCAGGAAAAAGCTGTAC 597

XM_7663892(mRNA) CACATAAAAGAAAGCATG-----------------------------GAAAAAGCTGTAC 568

V1(2) CACATAAAAGAAAGCATGGTTAGTTTCATTTTTATTAATTTGTTCAGGAAAAAGCTGTAC 597

V2(1) CACATAAAAGAAAGCATGGTTAGTTTTATTTTTATTAATTTGTTCAGGAAAAAGCTGTAC 532

V3(3) CACATAAAAGAAAGCATGGTTAGTTTTATTTTTATTAGTTTGTTCAGGAAAAAGCTGTAC 532

V4(1) CACATAAAAGAAAGCATGGTTAGTTTTATTTTTACTAATTTTTTCAGGAAAAAGCTGTAC 532

V5(4) CACATAAAAGAAAGCATGGTTAGTTTTATTCTTATTAATTTGCTCAGGAAAAAGCTGTAC 532

V6(3) CACATAAAAGAAAGCATGGTTAGTTTTATTTTTATTAATTTGTTCAGGAAAAAGCTGTAC 532

V7(1) CACATAAAAGAAAGCATGGTTAGTTTTATTTTTACTAATTTTTTCAGGAAAAAGCTGTAC 532

V8(1) CACATAAAAGAAAGCATGGTTAGTTTCATTTTTATTAATTTGTTCAGGAAAAAGCTGTAC 532

V9(1) CACATAAAAGAAAGCATGGTTAGTTTCATTTTTATTAATTTGTTCAGGAAAAAGCTGTAC 535

V10(5) CACATAAAAGAAAGCATGGTTAGTTTTATTTTTACTAATTTTTTCAGGAAAAAGCTGTAC 532

V11(1) CACATAAAAGAAAGCATGGTTAGTTTTATTTTTATTAGTTTGTTCAGGAAAAAGCTGTAC 532

V12(1) CACATAAAAGAAAGCATGGTTAGTTTTATTTTTATTAGTTTGTTCAGGAAAAAGCTGTAC 532

V13(1)* CACATAAAAGAAAGCATGGTTAGTTTTATTTTTACTAATTTTTTCAGGAAAAAGCTGTAC 532

****************** *************

TP01_0868(genomic) ACAGCCTTAAAAAGGTCTTGGAGGGGCTAACCAACCTTGCGACTCTGTCTAAAAGTAGGG 657

XM_7663892(mRNA) ACAGCCTTAAAAAGGTCTTGGAGGGGCTAACCAACCTTGCGACTCTGTCTAAAAGTAGGG 628

V1(2) ACAGCCTTAAAAAGGTCTTGGAGGGGCTAACCAACCTTGCGACTCTGTCTAAAAGTAGGG 657

V2(1) ACAGCCTTAAAAAGGTCTTGGAGGGGCTAACCAACCTTGCGACTCTGTCTAAAAGTAGGG 592

V3(3) ACAGCCTTAAAAAGGTCTTGGAGGGGCTAACCAACCTTGCGACTCTGTCTAAAAGTAGGG 592

V4(1) ACAGCCTTAAAAAGGTCTTGGAGGGGCTAACCAACCTTGCGACTCTGTCTAAAAGTAGGG 592

V5(4) ACAGCCTTAAAAAGGTCTTGGAGGGGCTAACCAACCTTGCGACTCTGTCTAAAAGTAGGG 592

V6(3) ACAGCCTTAAAAAGGTCTTGGAGGGGCTAACCAACCTTGCGACTCTGTCTAAAAGTAGGG 592

V7(1) ACAGCCTTAAAAAGGTCTTGGAGGGGCTAACCAACCTTGCGACTCTGTCTAAAAGTAGGG 592

V8(1) ACAGCCTTAAAAAGGTCTTGGAGGGGCTAACCAACCTTGCGACTCTGTCTAAAAGTAGGG 592

V9(1) ACAGCCTTAAAAAGGTCTTGGAGGGGCTAACCAACCTTGCGACTCTGTCTAAAAGTAGGG 595

V10(5) ACAGCCTTAAAAAGGTCTTGGAGGGGCTAACCAACCTTGCGACTCTGTCTAAAAGTAGGG 592

V11(1) ACAGCCTTAAAAAGGTCTTGGAGGGGCTAACTAACCTTGCGACTCTGTCTAAAAGTAGGG 592

V12(1) ACAGCCTTAAAAAGGTCTTGGAGGGGCTAACCAACCTTGCGACTCTGTCTAAAAGTAGGG 592

V13(1)* ACAGCCTTAAAAAGGTCTTGGAGGGGCTAACCAACCTTGCGACTCTGTCTAAAAGTAGGG 592

******************************* ****************************

TP01_0868(genomic) ATACTGAACCGTTTAATGTTCTGGGGGATGACTATACGATGCGTAACGTTTTGGACCTCA 717

XM_7663892(mRNA) ATACTGAACCGTTTAATGTTCTGGGGGATGACTATACGATGCGTAACGTTTTGGACCTCA 688

V1(2) ATACTGAACCGTTTAATGTTCTGGGGGATGACTATACGATGCGTAACGTTTTGGACCTCA 717

V2(1) ATACAGAACCGTTTAATGTTCTGGGGGATGACTATACGATGCGTAACGTTTTGGACCTCA 652

V3(3) ATACTGAACCGTTTAATGTTCTGGGGGATGACTATACGATGCGTAACGTTTTGGACCTCA 652

V4(1) ATACAGAACCGTTTAATGTTCTGGGGGATGACTATACGATGCGTAACGTTTTGGACCTCA 652

V5(4) ATACTGAACCGTTTAATGTTCTGGGGGATGACTATACGATGCGTAACGTTTTGGACCTCA 652

V6(3) ATACTGAACCGTTTAATGTTCTGGGGGATGACTATACGATGCGTAACGTTTTGGACCTCA 652

V7(1) ATACTGAACCGTTTAATGTTCTGGGGGATGACTATACGATGCGTAACGTTTTGGACCTCA 652

V8(1) ATACAGAACCGTTTAATGTTCTGGGGGATGACTATACGATGCGTAACGTTTTGGACCTCA 652

V9(1) ATACTGAACCGTTTAATGTTCTGGGGGATGACTATACGATGCGTAACGTTTTGGACCTCA 655

V10(5) ATACAGAACCGTTTAATGTTCTGGGGGATGACTATACGATGCGTAACGTTTTGGACCTCA 652

V11(1) ATACTGAACCGTTTAATGTTCTGGGGGATGACTATACGATGCGTAACGTTTTGGACCTCA 652

V12(1) ATACAGAACCGTTTAATGTTCTGGGGGATGACTATACGATGCGTAACGTTTTGGACCTCA 652

V13(1)* ATACTGAACCGTTTAATGTTCTGGGGGATGACTATACGATGCGTAACGTTTTGGACCTCA 652

**** *******************************************************

TP01_0868(genomic) TGAATAAGGAACTCAGGCAGGTTGAATCTCTTCAGAAAGTTGTGTTCCAATTCAACGCCT 777

XM_7663892(mRNA) TGAATAAGGAACTCAGGCAGGTTGAATCTCTTCAGAAAGTTGTGTTCCAATTCAACGCCT 748

V1(2) TGAATAAGGAACTCAGGCAGGTTGAATCTCTTCAGAAAGTTGTGTTCCAATTCAACGCCT 777

V2(1) TGAATAAGGAACTCAGGCAGGTTGAATCTCTTCAGAAAGTTGTGTTCCAATTCAACGCCT 712

V3(3) TGAATAAGGAACTCAGGCAGGTTGAATCTCTTCAGAAAGTTGTGTTCCAATTCAACGCCT 712

V4(1) TGAATAAGGAACTCAGGCAAGTTGAATCTCTTCAGAAAGTTGTGTTCCAATTCAACGCCT 712

V5(4) TGAATAAGGAACTCAGGCAGGTTGAATCTCTTCAGAAAGTCGTGTTCCAATTCAACGCCT 712

V6(3) TGAATAAGGAACTCAGGCAGGTTGAATCTCTTCAGAAAGTCGTGTTCCAATTCAACGCCT 712

V7(1) TGAATAAGGAACTCAGGCAGGTTGAATCTCTTCA-------------------------- 686

V8(1) TGAATAAGGAACTCAGGCAGGTTGAATCTCTTCAGAAAGTTGTGTTCCAATTCAACGCCT 712

V9(1) TGAATAAGGAACTCAGGCAGGTTGAATCTCTTCAGAAAGTTGTGTTCCAATTCAACGCCT 715

V10(5) TGAATAAGGAACTCAGGCAAGTTGAATCTCTTCAGAAAGTTGTGTTCCAATTCAACGCCT 712

V11(1) TGAATAAGGAACTCAGGCAGGTTGAATCTCTTCAGAAAGTTGTGTTCCAATTCAACGCCT 712

V12(1) TGAATAAGGAACTCAGGCAGGTTGAATCTCTTCAGAAAGTTGTGTTCCAATTCAACGCCT 712

V13(1)* TGAATAAGGAACTCAGGCAGGTTGAATCTCTTCAGAAAGTTGTGTTCCAATTCAACGCCT 712

******************* **************

TP01_0868(genomic) TTGCACTTTCC***ACCTTCACTAAGAGTCCAGACGATAAT***AAAAAATCCTAA 827

XM_7663892(mRNA) TTGCACTTTCCACCTTCACTAAGAGTCCAGACGATAATAAAAAATCCTAA 798

V1(2) TTGCACTTTCC--------------------------------------- 827

V2(1) TTGCACTTTCC--------------------------------------- 723

V3(3) TTGCACTTTCC--------------------------------------- 723

V4(1) TTGCACTTTCC--------------------------------------- 723

V5(4) TTGCACTTTCC--------------------------------------- 723

V6(3) TTGCACTTTCC--------------------------------------- 723

V7(1) -------------------------------------------------- 686

V8(1) TTGCACTTTCC--------------------------------------- 723

V9(1) TTGCACTTTCC--------------------------------------- 726

V10(5) TTGCACTTTCC--------------------------------------- 723

V11(1) TTG----------------------------------------------- 715

V12(1) TTGCCCTTTCC--------------------------------------- 723

V13(1)* TTGC---------------------------------------------- 716

Primer regions are in bold, underlined and italicized text.

* Partial sequence as defined by only having either the forward or reverse sequence of the allele available for analysis. This sequence is its own variant due to unique nucleotides.

**Table S5b: Tp3 protein variant Clustal alignment**

XP_7663892(2)MKLNTIAIAFLYSCFSQFLKNVSALRRSSPDLSPDGSFLQVKSASPQDKQDVIQSSSPKV 60

V2(1) ---------------------VSALRRSSPDLSPDGSFLQVKSASPQDKQDVIQSSSAKV 39

V3(3) ---------------------VSALRRSSPDLSPDGSFLQVKSASPQDKQDVIQSSSAKV 39

V4(3) ---------------------VSALRRSSPDLSPDGSFLQVKSASPQDKQDVIQSSSAKV 39

V5(1) ---------------------VSALRRSSPDLSPDGSFLQVKSASPQDKQDVIQSSSPKV 39

V6(1) ---------------------VSALRRSSPDLSPDGSFLQVKSASPQDKQDVIQSSSPKV 39

V7(1) ---------------------VSALRRSSPDLSPDGSFLQVKSASPQDKQDVIQSSSPKV 39

V8(3) ---------------------VSALRRSSPDLSPDGSFLQVKSASPQDKQDVIQSSSPKV 39

V9(9) ---------------------VSALRRSSPDLSPDGSFLQVKSASPQDKQDVIQSSSPKV 39

************************************ **

XP_7663892(2)TVPTVDPEGLKKAVTAAVLSNQNQALQNGALNPADFTQAASV-NSMSNAVSAMNNTVGPV 119

V2(1) TVPAVDPEGLKKAVTAPVLSNQNQALQNGALNPADFTQAAAV-NSMSNAVSAMNNTVGPA 98

V3(3) TVPAVDPEGLKKAVTAPVLSNQNQALQNGALNPADFTQAAAV-NSMSNAVSAMNNTVGPA 98

V4(3) TVPAVDPEGLKKAVTAAVLSNQNQALQNGALNPADFTQAAAV-NSMSNAVSAMNNTVGPA 98

V5(1) TVPAVDPEGLKKAVTAAVLSNQNQALQNGALNPADFTQAAAV-NSMSNAVSAMNNTVGPA 98

V6(1) TVPAVDPEGLKKAVTAAVLSNQNQALQNGALNPADFTQAAAV-NSMSNAVSAMNNTVGPV 98

V7(1) TVPTVDPEGLKKAVTAAVLSNQNQALQNGALNPADFTQATSV-NSMSNAVSAMNNTVGPV 98

V8(3) TVPTVDPEGLKKAVTAAVLSNQNQALQNGALNPADFTQAAAV-NSMSNAVGAMNNTVGPV 98

V9(9) TVPTVDPEGLKKAVTAAVLSNQNQALQNGALNPADFTQAASV-NSMSNAVSAMNNTVGPV 98

***:************ **********************::* *******.********.

XP_7663892(2)KNPMATVGTMNSFTGMPGVQDNFPQTPPVNVQDTSTQENSLDNLNLLLDPSLVKISQADS 179

V2(1) -NPMATVGTMNSFTGTPGVQDNFPQTPPVNVQDTSTQENSLDNLNLLLDPSLVKISQADS 157

V3(3) -NPMATVGTMNSFTGTPGVQDTFSQTPPVNVQDTSTQENSLDNLNLLLDPSLLKISQADS 157

V4(3) -NPMATVGTMNSFTGTPGVQDTFSQTPPVNVQDTSTQENSLDNLNLLLDPSLLKISQADS 157

V5(1) -NPMATVGTMNSFTGTPGVQDTFSQTPPVNVQDTSTQENSLDNLNLLLDPSLAKISQADS 157

V6(1) -NPMATVGTMNSFTGTPGVQDTFPQTPPVNVQDTSTQENSLDNLNLLLDPSLVKISQADS 157

V7(1) KNPMATVGTMNSFTGMPGVQDNFPQTPPVNVQDTSTQENSLDNLNLLLDPSLVKISQADS 158

V8(3) -NPMTTVGTMNSFTGTPGVQDNFPQTPPVNVQDTSTQENSLDNLNLLLDPSLLKISQADS 157

V9(9) -NPMATVGTMNSFTGMPGVQDNFPQTPPVNVQDTSTQENSLDNLNLLLDPSLVKISQADS 157

***:********** ******* **************************** *******

XP_7663892(2)HIKESMEKAVHSLKKVLEGLTNLATLSKSRDTEPFNVLGDDYTMRNVLDLMNKELRQVES 239

V2(1) HIKESMEKAVHSLKKVLEGLTNLATLSKSRDTEPFNVLGDDYTMRNVLDLMNKELRQVES 217

V3(3) HIKESMEKAVHSLKKVLEGLTNLATLSKSRDTEPFNVLGDDYTMRNVLDLMNKELRQVES 217

V4(3) HIKESMEKAVHSLKKVLEGLTNLATLSKSRDTEPFNVLGDDYTMRNVLDLMNKELRQVES 217

V5(1) HIKESMEKAVHSLKKVLEGLTNLATLSKSRDTEPFNVLGDDYTMRNVLDLMNKELRQVES 217

V6(1) HIKESMEKAVHSLKKVLEGLTNLATLSKSRDTEPFNVLGDDYTMRNVLDLMNKELRQVES 217

V7(1) HIKESMEKAVHSLKKVLEGLTNLATLSKSRDTEPFNVLGDDYTMRNVLDLMNKELRQVES 218

V8(3) HIKESMEKAVHSLKKVLEGLTNLATLSKSRDTEPFNVLGDDYTMRNVLDLMNKELRQVES 217

V9(9) HIKESMEKAVHSLKKVLEGLTNLATLSKSRDTEPFNVLGDDYTMRNVLDLMNKELRQVES 217

************************************************************

XP_7663892(2)LQKVVFQFNAFALSTFTKSPDDNKKS 265

V2(1) LQKVVFQFNAFALS------------ 231

V3(3) LQKVVFQFNAFALS------------ 231

V4(3) LQKVVFQFNAFALS------------ 231

V5(1) LQKVVFQFNAFALS------------ 231

V6(1) L------------------------- 218

V7(1) LQKVVFQFNAFALS------------ 232

V8(3) LQKVVFQFNAFALS------------ 231

V9(9) LQKVVFQFNAFALS------------ 231

Eptiope sequence unknown.

**Table S6a: Tp4 nucleotide variant Clustal alignment**

TP03_0210 GTAGTTTTATCTAAATTG***CCAATTGGTGATTTAGCAACA***CAATACTTTGCAGGTATTTAC 1377

XM_758135 GTAGTTTTATCTAAATTGCCAATTGGTGATTTAGCAACACAATACTTTGCAGATA----- 922

V1(2) ---------------------------------------CAATACTTTGCAGGTATTTAC 21

V2(2) ---------------------------------------CAATACTTTGCAGGTATTTAC 21

V3(4) ---------------------------------------CAATACTTTGCAGGTATTTAC 21

V4(2) ---------------------------------------CAATACTTTGCAGGTATTTAC 21

V5(2) ------------------------------------------TACTTTGCAGGTATTTAC 18

V6(1) ---------------------------------------CAATACTTTGCAGGTATTTAC 21

V7(1) ---------------------------------------CAATACTTTGCCGGTATTTAC 21

V8(1) ---------------------------------------CAATACTTTGCAGGTATTTAC 21

V9(2) ---------------------------------------CAATACTTTGCAGGTATTTAC 21

V10(1) ---------------------------------------CAATACTTTGCAGGTATTTAC 21

V11(1) ---------------------------------------CAATACTTTGCAGGTATTTAC 21

V12(2) ---------------------------------------------TTTGCAGGTATTTAC 15

V13(3) ---------------------------------------CAATACTTTGCAGGTATTTAC 21

***** ****

TP03_0210 GCAGCTTGTACTCCCCCACGGGAACTTAGTTATACAAATT-TATTCTAGATTACTTATAT 1436

XM_758135 ------------------------------------------------------------ 922

V1(2) GCAGCTTGTACTCCCCCACGGGAACTTAGTTATACAAATT-TATTCTAGATTACTTATAT 80

V2(2) GCAGCTTGTACATCCCCACGGGAACTTAGTTATACAAATT-TATTCTAGATTACTTATAT 80

V3(4) GGAGCTTGTACATCCCCACGGGAACTTAGTTACACAAATTTAACACTAGATTAGTTATAT 81

V4(2) GGAGCTTGTACATCCCCACGGGAACTTAGTTACACACTTTTAACACCAGATTAGTTATAT 81

V5(2) GCAGCTTGTACTCCCCCACGGGAACTTAGTTACACAAATTTAACACTATATTAGTTATAT 78

V6(1) GGAGCTTGTACATCCCCACGGGAACTTAGTTATACACTTTTAACACCAGATTAGTTATAT 81

V7(1) GCAGCTTGTACTCCCCCACGGGAACTTAGTTATACACTTTTAACACCAGATTAGTTATAT 81

V8(1) GGAGCTTGTACATCCCCACGGGAACTTAGTTACACAAATTTAACACTAGATTAGTTATAT 81

V9(2) GGAGCTTGTACATCCCCACGGGAACTTAGTTATACACTTTTAACACCAGATTAGTTATAT 81

V10(1) GGAGCTTGTACATCCCCACGGGAACTTAGTTATACACTTTTAACACCAGATTAGTTATAT 81

V11(1) GCAGCTTGTACTCCCCCACGGGAACTTAGTTATATACTTTTAACACTATATTAGTTATAT 81

V12(2) GGAGCTTGTACTCCCCCACGGGAACTTAGTTATACAAATT-TATTCTAGATTACTTATAT 74

V13(3) GGAGCTTGTACATCCCCACGGGAACTTAGTTACACAAATTTAACACTAGATTAGTTATAT 81

TP03_0210 CCCGTACAATATTAATAATTTTCCTTAGATAAAAATGTATTTTGTGCCGGCCGGGTTGAT 1496

XM_758135 -------------------------------AAAATGTATTTTGTGCCGGCCGGGTTGAT 951

V1(2) CCCGTACAATATTAATAATTTTCCTTAGATAAAAATGTATTTTGTGCCGGCCGGGTTGAT 140

V2(2) CCCGTACAATATTAATAATTTTCCTTAGATAAAAATGTATTTTGTGCCGGCCGGGTTGAT 140

V3(4) ACTATACAATATTAATAATTTTCCTTAGATAAGAATGTATTTTGTGCCGGTCGCGTTGAT 141

V4(2) TGCATACAATATTAACAATTTTCCTTAGATAAGAATGTATTTTGTGCCGGTCGCGTTGAT 141

V5(2) TGCATACAATATTAACAATTTTCCTTAGATAAGAATGTATTTTGTGCCGGTCGTGTTGAT 138

V6(1) ACTATACAATATTAACAATTTTTTATAGATAAGAATGTATTTTGTGCCGGTCGTGTTGAT 141

V7(1) TGCATACAATATTAACAATTTTTTATAGATAAGAATGTATTTTGTGCCGGTCGTGTTGAT 141

V8(1) ACTATACAATATTAATAATTTTCCTTAGATAAGAATGTATTTTGTGCCGGCCGGGTTGAT 141

V9(2) ACTATACAATATTAACAATTTTTTATAGATAAGAATGTATTTTGTGCCGGTCGTGTTGAT 141

V10(1) ACTATACAATATTAACAATTTTTTATAGATAAGAATGTATTTTGTGCCGGTCGTGTTGAT 141

V11(1) TGCATACAATATTAACAATTTTCCTTAGATAAGAATGTATTTTGTGCCGGTCGCGTTGAT 141

V12(2) GCCATACAATATTAATAATTTTCCTTAGATAAGAATGTATTTTGTGCCGGTCGTGTTGAT 134

V13(3) ACTATACAATATTAATAATTTTCCTTAGATAAGAATGTATTTTGTGCCGGCCGGGTTGAT 141

* ***************** ** ******

TP03_0210 GAAAATGATCTTATAAGAACGAGTAAAGCTACTGGTGCTTCTATTCAAACCACTCTCAAT 1556

XM_758135 GAAAATGATCTTATAAGAACGAGTAAAGCTACTGGTGCTTCTATTCAAACCACTCTCAAT 1011

V1(2) GAAAATGATCTTATAAGAACGAGTAAAGCTACTGGTGCTTCTATTCAAACCACTCTCAAT 200

V2(2) GAAAATGATCTTATAAGAACGAGTAAAGCTACTGGTGCTTCTATTCAAACCACTCTCAAT 200

V3(4) GAAAATGATCTTGTAAGAACGAGTAAAGCTACTGGTGCTTCTATTCAAACCACTCTCAAT 201

V4(2) GAAAATGATCTTATAAGAACGAGTAAAGCCACTGGTGCTTCTATTCAAACCACTCTCAAC 201

V5(2) GAAAATGATCTTATAAGAACGAGTAAAGCTACTGGTGCTTCCATTCAAACCACTCTCAAT 198

V6(1) GAAAATGATCTTATAAGAACGAGTAAAGCTACTGGTGCTTCCATTCAAACCACTCTCAAT 201

V7(1) GAAAATGATCTTATAAGAACGAGTAAAGCTACTGGTGCTTCTATTCAAACCACTCTCAAT 201

V8(1) GAAAATGATCTTATAAGAACGAGTAAAGCTACTGGTGCTTCTATTCAAACCACTCTCAAT 201

V9(2) GAAAATGATCTTATAAGAACGAGTAAAGCTACTGGTGCTTCCATTCAAACCACTCTCAAT 201

V10(1) GAAAATGATCTTATAAGAACGAGTAAAGCTACTGGTGCTTCCATTCAAACCACTCTCAAT 201

V11(1) GAAAATGATCTTATAAGAACGAGTAAAGCTACTGGTGCTTCYATTCAAACCACTCTCAAT 201

V12(2) GAAAATGATCTTATAAGAACGAGTAAAGCTACTGGTGCTTCTATTCAAACCACTCTCAAT 194

V13(3) GAAAATGATCTTATAAGAACGAGTAAAGCTACTGGTGCTTCTATTCAAACCACCCTCAAT 201

************ **************** *********** *********** *****

TP03_0210 AACCTTTCAGTTGACGTCTTAGGTACTGCGTTGCTTGCAGTCAATTCCTGCTACAATCTA 1616

XM_758135 AACCTTTCAGTTGACGT------------------------------------------- 1028

V1(2) AACCTTTCAGTTGACGTCTTAGGTACTGCGTTGCTTGCAGTCAATTCCTGCTACAATCTA 260

V2(2) AACCTTTCAGTTGACGTCTTAGGTACTGCGTTGCTTGCAGGCAATTCCTGCTACAATCTA 260

V3(4) AACCTTTCAGTTGACGTCTTAGGTACTGCGTTGCTTGCAGTCAATTCCTGCTACAATCTA 261

V4(2) AACCTTTCAGTTGACGTCTTAGGTACTGCGTTGCGTGCAGGCAATTCCTGCTACAATCTA 261

V5(2) AATCTTTCAGTTGACGTCTTAGGTACTGCGTTTCTTGCAGGCAATTCCTGCTACAATCTA 258

V6(1) AACCTTTCAGTTGACGTCTTAGGTACTGCGTTGCTTGCAGGCAATTCCTGCTACAATCTA 261

V7(1) AACCTTTCAGTTGACGTCTTAGGTACTGCGTTGCTTGCAGGCAATTCCTGCTACAATCTA 261

V8(1) AACCTTTCAGTTGACGTCTTAGGTACTGCGTTGCTTGCAGTCAATTCCTGCTACAATCTA 261

V9(2) AACCTTTCAGTTGACGTCTTAGGTACTGCGTTGCTTGCAGGCAATTCCTGCTACAATCTA 261

V10(1) AACCTTTCAGTTGACGTCTTAGGTACTGCGTTGCTTGCAGGCAATTCCTGCTACAATCTA 261

V11(1) AACCTTTCAGTTGACGTCTTAGGTACTGCGTTGCTTGCAGGCAATTCCTGCTACAATATA 261

V12(2) AACCTTTCAGTTGACGTCTTAGGTACTGCGTTGCTTGCAGGCAATTCCTGCTACAATCTA 254

V13(3) AACCTTTCAGTTGACGTCTTAGGTACTGCGTTGCGTGCAGGCAATTCCTGCTACAATCTA 261

** **************

TP03_0210 CTCAGCCTACTAGTTATTTATACTTTAGTGTTCACTTATTTATCCCATCCTGGA-TTCTA 1675

XM_758135 ------------------------------------------------------------ 1028

V1(2) CTCAGCCTACTAGTTATTTATACTTTAGTGTTCACTTATTTATCCCATCCTGGA-TTCTA 319

V2(2) CTCAGCCTACTAGTTATTTATACTTTAGTGTTCACTTACTTATCCCATCCTGGA-TTCTA 319

V3(4) CTCAGTTTACTAGTTATTCATCACCTACTTTGCACTTATTTATCCCATCCTGGA-TTCTA 320

V4(2) CTCAGTTTACTAGTTATTCATCACCTACTTTGCACTTATTTATCCCATCCTGGGTTTGTA 321

V5(2) CTCAGCCTACTAGTTATTTATACTTTAGTGTTCACTTATTTATCCCATCCTGGA-TTCTA 317

V6(1) CTCAGCCTACTAGTTATTTATACTTTAGTGTTCACTTATTTATCCCATCCTGGA-TTCTA 320

V7(1) CTCAGTTTACTAGTTATTTATACCCTACTTTGCAGTTATTTATCCCATCCTGGA-TTCTA 320

V8(1) CTCAGCCTACTAGTTATTTATACTTTAGTGTTCACTTATTTATCCCATCCTGGA-TTCTA 320

V9(2) CTCAGCCTACTAGTTATTTATACTTTAGTGTTCACTTATTTATCCCATCCTGGA-TTCTA 320

V10(1) CTCAGCCTACTAGTTATTTATACTTTAGTGTTCACTTATTTATCCCATCCTGGA-TTCTA 320

V11(1) CTCAGTTTAATAGTTATTTATCACCTACTKTGCAGTTATTTATCCCATCCTGGA-TTCTA 320

V12(2) CTCAGTTTAATAGTTATTTATCACCTACTGTGCACTTATTTATCCCATCCTGGGTTTGTA 314

V13(3) CTCAGTTTAATAGTTATTTATCACCTACTGTGCACTTATTTATCCCATCCTGGA-TTCTA 320

TP03_0210 TTCATCATTTACACTAGATTTACATGTTTTATTTATTGTATTCCACTTTTTAAATATAAT 1735

XM_758135 ------------------------------------------------------------ 1028

V1(2) TTCATCATTTACACTAGATTTACATGTTTTATTTATTGTATTCCACTTTTTAAATATAAT 379

V2(2) TTCATCATTTACACTAGATTTACATGTTTTATTTATTGTATTCCACTTTTTAAATATAAT 379

V3(4) TTCATCATTTACACCAGATTTACATGCTTTATTTATTGTATTCCACTTTTTAAATATAAT 380

V4(2) TTCATCATTTACACTAGATTTACATGCTACTTATGAGTTATTCCACTTTTTAAATATAAT 381

V5(2) TTCATCATTTAAACTAGATTTACATGGTTTCTTTATTGTATTCCACTTT-TAAATACAAT 376

V6(1) TTCATCATTTACACTAGATTTACATGTTTTATTTATTGTATTCCACCTTTTAAATATAAT 380

V7(1) TTCATCATTTACACCAGATTTACATTCTTTATTTATTGTATTTCACCTTTTAAATATAAT 380

V8(1) TTCATCATTTACACTAGATTTACATGTTTTATTTATTGTATTCCACTTTTTAAATATAAT 380

V9(2) TTCATCATTTACACTAGATTTACATGTTTTATTTATTGTATTCCACTTTTTAAATATAAT 380

V10(1) TTCATCATTTACACTAGATTTACATGTTTTATTTATTGTATTCCACCTTTTAAATATAAT 380

V11(1) TTCATCATTTACACTAGATTTACATGGTTTCTTTGTTGTATTTCACCTTTTAAATATAAT 380

V12(2) TTCATCATTTAAACTAGATTTACATTCTTTATTTATTGTATTCCACTTTTTAAATATAAT 374

V13(3) TTCATCATTTACACCAGATTTACATGGTTTCTTTATTGTATTCCACTTTTTAAATATAAT 380

TP03_0210 TTTATAATGGTTTATTAGGAACTTGTGGTGTGTTTGAGGAAGTGCAAATTGGGTCTGAAC 1795

XM_758135 -------------CTTAGGAACTTGTGGTGTGTTTGAGGAAGTGCAAATTGGGTCTGAAC 1075

V1(2) TTTATAATGGTTTATTAGGAACTTGTGGTGTGTTTGAGGAAGTGCAAATTGGGTCTGAAC 439

V2(2) TTTATAATGGTTTATTAGGAACTTGTGGTGTGTTTGAGGAAGTGCAAATTGGGTCTGAAC 439

V3(4) TTTATAATGGTTTATTAGGAACTTGTGGTGTGTTTGAGGAAGTGCAAATTGGGTCTGAAC 440

V4(2) TTTATAATGGTTTATTAGGAACTTGTGGAGTGTTTGAGGAGGTCCAAATTGGCTCTGAAC 441

V5(2) TTTATAATGGTTTATTAGGAACTTGTGGAGTGTTTGAGGAGGTCCAAATTGGCTCTGAAC 436

V6(1) TTTATAATGGTTTATTAGGAACTTGTGGAGTGTTTGAGGAAGTGCAAATTGGGTCTGAAC 440

V7(1) TTTATAATGGTTTATTAGGAACTTGTGGAGTGTTTGAGGAAGTCCAAATTGGGTCTGAAC 440

V8(1) TTTATAATGGTTTATTAGGAACTTGTGGTGTGTTTGAGGAAGTGCAAATTGGGTCTGAAC 440

V9(2) TTTATAATGGTTTATTAGGAACTTGTGGTGTGTTTGAGGAAGTGCAAATTGGGTCTGAAC 440

V10(1) TTTATAATGGTTTATTAGGAACTTGTGGAGTGTTTGAGGAAGTGCAAATTGGGTCTGAAC 440

V11(1) TTTATAATGGTTTATTAGGAACTTGTGGAGTGTTTGAGGAAGTCCAAATTGGMTCTGAAC 440

V12(2) TTTATAATGGTTTATTAGGAACTTGTGGAGTGTTTGAGGAGGTCCAAATTGGGTCTGAAC 434

V13(3) TTTATACTGGTTTATTAGGAACTTGTGGAGTGTTTGAGGAGGTCCAAATTGGCTCTGAAC 440

************** *********** ** ******** *******

TP03_0210 GTTACAATATGTTCACAGATTGCAAGAGTGCAAAAACCTGTACAATTGTGTTGAGAGGTG 1855

XM_758135 GTTACAATATGTTCACAGATTGCAAGAGTGCAAAAACCTGTACAATTGTGTTGAGAGGTG 1135

V1(2) GTTACAATATGTTCACAGATTGCAAGAGTGCAAAAACCTGTACAATTGTGTTGAGAGGTG 499

V2(2) GTTACAATATGTTCACAGATTGCAAGAGTGCAAAAACCTGTACAATTGTGTTGAGAGGTG 499

V3(4) GTTACAATATGTTCACAGATTGCAAGAGTGCAAAAACCTGTACAATTGTGTTGAGAGGTG 500

V4(2) GTTACAATATGTTCACAGATTGCAAGAGTGCAAAAACCTGTACAATTGTGTTGAGAGGTG 501

V5(2) GTTACAATATGTTCACAGATTGCAAGAGTGCAAAAACCTGTACAATTGTGTTGAGAGGTG 496

V6(1) GTTACAATATGTTCACAGATTGCAAGAGTTCAAAAACCTGTACAATTGTGTTGAGAGGTG 500

V7(1) GTTACAATTTGTTCACCGATTGCAAGAGTGCAAAAACCTGTACAATTGTGCTGAGAGGTG 500

V8(1) GTTACAATATGTTCACAGATTGCAAGAGTGCAAAAACCTGTACAATTGTGTTGAGAGGTG 500

V9(2) GTTACAATATGTTCACAGATTGCAAGAGTGCAAAAACCTGTACAATTGTGTTGAGAGGTG 500

V10(1) GTTACAATATGTTCACAGATTGCAAGAGTGCAAAAACCTGTACAATTGTGTTGAGAGGTG 500

V11(1) GTTACAATATGTTCACAGATTGCAAGAGTGCAAAAACCTGTACAATTGTGTTGAGAGGTG 500

V12(2) GTTACAATTTGTTCACAGATTGCAAGAGTGCAAAAACCTGTACAATTGTGTTGAGAGGTG 494

V13(3) GTTACAATTTGTTCACAGATTGCAAGAGTGCAAAAACCTGTACAATTGTGTTGAGAGGTG 500

******** ******* ************ ******************** *********

TP03_0210 GAGGTCAGCAGTTCATTGATGAATCTGAACGTTCACTCCATGACGCGATTATGATTGTCA 1915

XM_758135 GAGGTCAGCAGTTCATTGATGAATCTGAACGTTCACTCCATGACGCGATTATGATTGTCA 1195

V1(2) GAGGTCAGCAGTTCATTGATGAATCTGAACGTTCACTCCATGACGCGATTATGATTGTCA 559

V2(2) GAGGTCAGCAGTTCATTGATGAATCTGAACGTTCACTCCATGACGCGATTATGATTGTCA 559

V3(4) GAGGTCAGCAGTTCATTGATGAATCTGAACGTTCACTCCATGACGCGATTATGATTGTCA 560

V4(2) GAGGTCAGCAGTTCATTGATGAATCTGAACGTTCACTCCATGACGCGATTATGATTGTCA 561

V5(2) GAGGTCAGCAGTTCATTGATGAATCTGAACGTTCACTCCATGACGCAATTATGATTGTCA 556

V6(1) GGGGTCAGCAGTTCATTGATGAATCTGAACGTTCACTCCATGACGCAATTATGATTGTCA 560

V7(1) GAGGTCAGCAGTTCATTGATGAATCTGAACGTTCACTCCATGACGCAATTATGATTGTCA 560

V8(1) GAGGTCAGCAGTTCATTGATGAATCTGAACGTTCACTCCATGACGCGATTATGATTGTCA 560

V9(2) GGGGTCAGCAGTTCATTGATGAATCTGAACGTTCACTCCATGACGCGATTATGATTGTCA 560

V10(1) GGGGTCAGCAGTTCATTGATGAATCTGAACGTTCACTCCATGACGCAATTATGATTGTCA 560

V11(1) GGGGTCAGCAGTTCATTGATGAATCTGAACGTTCACTCCATGACGCGATTATGATTGTCA 560

V12(2) GGGGTCAGCAGTTCATTGATGAATCTGAACGTTCACTCCATGACGCAATTATGATTGTCA 554

V13(3) GAGGTCAGCAGTTCATTGATGAATCTGAACGTTCACTCCATGACGCGATTATGATTGTCA 560

* ******************************************** *************

TP03_0210 GAAGAGCAACTAAATGTAATACTATCCTTCCCGGAGCTGGTGCCATTGAGATGTTGCTCT 1975

XM_758135 GAAGAGCAACTAAATGTAATACTATCCTTCCCGGAGCTGGTGCCATTGAGATGTTGCTCT 1255

V1(2) GAAGAGCAACTAAATGTAATACTATCCTTCCCGGAGCTGGTGCCATTGAGATGTTGCTCT 619

V2(2) GAAGAGCAACTAAATGTAATACTATCCTTCCCGGAGCTGGCGCTATTGAGATGTTGCTCT 619

V3(4) GAAGAGCAACTAAATGTAATACTATCCTTCCCGGCGCCGGCGCCATTGAAATGTTACTCT 620

V4(2) GAAGAGCAACTAAATGTAATACTATCCTTCCCGGAGCTGGTGCCATTGAGATGTTACTCT 621

V5(2) GAAGAGCAACTAAATGTAATACTATCCTTCCCGGAGCTGGCGCCATTGAGATGTTGCTCT 616

V6(1) GAAGAGCAACTAAGTGTAATACTATTCTTCCCGGCGCCGGCGCCATTGAGATGTTACTCT 620

V7(1) GAAGAGCAACTAAATGTAATACTATCCTTCCCGGAGCTGGCGCCATTGAGATGTTACTCT 620

V8(1) GAAGAGCAACTAAGTGTAATACTATTCTTCCCGGCGCCGGCGCCATTGAGATGTTGCTCT 620

V9(2) GAAGAGCAACTAAATGTAATACTATCCTTCCCGGAGCTGGCGCCATTGAGATGTTGCTCT 620

V10(1) GAAGAGCAACTAAGTGTAATACTATTCTTCCCGGCGCCGGCGCCATTGAGATGTTACTCT 620

V11(1) GAAGAGCAACTAAATGTAATACTATCCTTCCCGGMGCCGGCGCCATTGAGATGTTACTCT 620

V12(2) GAAGAGCAACTAAATGTAATACTATCCTTCCCGGAGCTGGTGCCATTGAGATGTTGCTCT 614

V13(3) GAAGAGCAACTAAATGTAATACTATTCTTCCCGGCGCCGGCGCCATTGAGATGTTGCTCT 620

************* *********** ******** ** ** ** ***** ***** ****

TP03_0210 CAACTTATCTCCTCCACTATTCTCTCAACACTATTAATCCCACAGACTCTGTCAACCATG 2035

XM_758135 CAACTTATCTCCTCCACTATTCTCTCAACACTATTAATCCCACAGACTCTGTCAACCATG 1315

V1(2) CAACTTATCTCCTCCACTATTCTCTCAACACTATTAATCCCACAGACTCTGTCAACCATG 679

V2(2) CAACTTATCTCCTCCACTATTCTCTCAACACTATTAATCCCACAGACTCAGTTAACCATG 679

V3(4) CAACTTATCTCCTACACTACTCTCTCAACACTATTAATCCCACAGACTCAGTTAACCATG 680

V4(2) CAACTTATCTCCTCCACTATTCTCTCAACACTATTAATCCCACAGACTCAGTTAACCATG 681

V5(2) CAACTTATCTCCTCCACTATTCTCTCAACACTATCAATCCCACAGACTCAGTTAACCATG 676

V6(1) CAACTTATCTCCTACACTATTCTCTCAACACTATTAATCCCACAGACTCAGTTAACCATG 680

V7(1) CAACTTATCTCCTACACTATTCTCTCAACACTATTAATCCCACAGACTCAGTTAACCATG 680

V8(1) CAACTTATCTCCTACACTATTCTCTCAACACTATTAATCCCACAGACTCAGTTAACCATG 680

V9(2) CAACTTATCTCCTCCACTATTCTCTCAACACTATTAATCCCACAGACTCAGTTAACCATG 680

V10(1) CAACTTATCTCCTACACTATTCTCTCAACACTATTAATCCCACAGACTCAGTTAACCATG 680

V11(1) CAACTTATCTCCTCCACTATTCTCTCAACACTATTAATCCCACAGACTCAGTTAACCATG 680

V12(2) CAACTTATCTCCTACACTACTCTCTCAACACTATTAATCCCACAGACTCAGTTAACCATG 674

V13(3) CAACTTATCTCCTCCACTATTCTCTCAACACTATTAATCCCACAGACTCAGTTAACCATG 680

************* ***** ************** ************** ** *******

TP03_0210 TTAACTGCGTTAA***CTCCGTAAATCATGTTAATGGAGT***TACTGGGGTGAATAAGAGTCTGG 2095

XM_758135 TTAACTGCGTTAACTCCGTAAATCATGTTAATGGAGTTACTGGGGTGAATAAGAGTCTGG 1375

V1(2) TTAACTGCGTTAA----------------------------------------------- 692

V2(2) TTAACTGCGTTAA----------------------------------------------- 692

V3(4) TTAACTGCGTTAA----------------------------------------------- 693

V4(2) TTAACTGCGTTAA----------------------------------------------- 694

V5(2) TTAACTGCGTTAA----------------------------------------------- 689

V6(1) TTAACTGCGTTAA----------------------------------------------- 693

V7(1) TTAACTGCGTTAA----------------------------------------------- 693

V8(1) TTAACTGCCTTAA----------------------------------------------- 693

V9(2) TTAACTTCGTTAA----------------------------------------------- 693

V10(1) TTAACTGCGTTAA----------------------------------------------- 693

V11(1) TTAACTGCGTTAA----------------------------------------------- 693

V12(2) TTAACTGCGTTAA----------------------------------------------- 687

V13(3) TTAACTGCGTTAA----------------------------------------------- 693

****** * ****

Primer regions are in bold, underlined and italicized text.

**Table S6b: Tp4 protein variant Clustal alignment**

XP_763228 ATQYFADKNVFCAGRVDENDLIRTSKATGASIQTTLNNLSVDVLGTCGVFEEVQIGSERY 360

V1(10) --QYFADKNVFCAGRVDENDLIRTSKATGASIQTTLNNLSVDVLGTCGVFEEVQIGSERY 58

V2(1) --QYFADKNVFCAGRVDENDLIRTSKATGASIQTTLNNLSVDVLGTCGVFEEVQIGSERY 58

V3(6) --QYFADKNVFCAGRVDENDLIRTSKATGASIQTTLNNLSVDVLGTCGVFEEVQIGSERY 58

V4(4) --QYFADKNVFCAGRVDENDLVRTSKATGASIQTTLNNLSVDVLGTCGVFEEVQIGSERY 58

V5(2) --QYFADKNVFCAGRVDENDLIRTSKATGASIQTTLNNLSVDVLGTCGVFEEVQIGSERY 58

V6(1) --QYFADKNVFCAGRVDENDLIRTSKATGASIQTTLNNLSVDVLGTCGVFEEVQIGSERY 58

******************* **************************************

XP_763228 NMFTDCKSAKTCTIVLRGGGQQFIDESERSLHDAIMIVRRATKCNTILPGAGAIEMLLST 420

V1(10) NMFTDCKSAKTCTIVLRGGGQQFIDESERSLHDAIMIVRRATKCNTILPGAGAIEMLLST 118

V2(1) NMFTDCKSSKTCTIVLRGGGQQFIDESERSLHDAIMIVRRATKCNTILPGAGAIEMLLST 118

V3(6) NLFTDCKSAKTCTIVLRGGGQQFIDESERSLHDAIMIVRRATKCNTILPGAGAIEMLLST 118

V4(4) NMFTDCKSAKTCTIVLRGGGQQFIDESERSLHDAIMIVRRATKCNTILPGAGAIEMLLST 118

V5(2) NMFTDCKSAKTCTIVLRGGGQQFIDESERSLHDAIMIVRRATKCNTILPGAGAIEMLLST 118

V6(1) NMFTDCKSAKTCTIVLRGGGQQFIDESERSLHDAIMIVRRATKCNTILPGAGAIEMLLST 118

* ****** ***************************************************

XP_763228 YLLHYSLNTINPTDSVNHVNCVNSVNHVNGVTGVNKSLVGKRHIIMNGFAKALECIPRNL 480

V1(10) YLLHYSLNTINPTDSVNHVNCV-------------------------------------- 140

V2(1) YLLHYSLNTINPTDSVNHVNCV-------------------------------------- 140

V3(6) YLLHYSLNTINPTDSVNHVNCV-------------------------------------- 140

V4(4) YLLHYSLNTINPTDSVNHVNCV-------------------------------------- 140

V5(2) YLLHYSLNTINPTDSVNHVNFV-------------------------------------- 140

V6(1) YLLHYSLNTINPTDSVNHVNCL-------------------------------------- 140

********************

Eptiope region highlighted in grey.

**Table S7a: Tp5 nucleotide variant Clustal alignment**

TP02_0767(genomic) TAGAGGAAAG***ATGAGGAAGCGAGTTTGGG***TAAATGCCGGCGATATTATTTTGGTATCGCT 539

XM_765334(mRNA) TAGAGGAAAGATGAGGAAGCGAGTTTGGGTAAATGCCGGCGATATTATTTTGGTATCGCT 301

V1(10) ------------------------------------------------------------ 0

V2(1)* ------------------------------------------------------------ 0

V3(2) -----------------------------TAAATGCCGGCGATATTATTTTGGTATCGCT 31

V4(1) -----------------------------TAAATGCCGGCGATATTATTTTGGTATCGCT 31

V5(1)* -----------------------------TAAATGCCGGCGATATTATTTTGGTATCGCT 31

V6(1)* ------------------------------------------------------------ 0

TP02_0767(genomic) TAGAGATTTCCAGGACAGCAAGGCTGACGTGATCGCAAAGTACACTGCTGAGGAGGCTCG 599

XM_765334(mRNA) TAGAGATTTCCAGGACAGCAAGGCTGACGTGATCGCAAAGTACACTGCTGAGGAGGCTCG 361

V1(10) -------TTCCAGGACAGCAAGGCTGACGTGATCGCAAAGTACACTGCTGAGGAGGCTCG 53

V2(1)* -------TTCCAGGACAGCAAGGCTGACGTGATCGCAAAGTACACTGCTGAGGAGGCTCG 53

V3(2) TAGAGATTTCCAGGACAGCAAGGCTGACGTGATCGCAAAGTACACTGCTGAGGAGGCTCG 91

V4(1) TAGAGATTTCCAGGACAGCAAGGCTGACGTGATCGCAAAGTACACCGCTGAGGAGGCGCG 91

V5(1)* -------TTCCAGGACAGCAAGGCTGACGTGATCGCAAAGTACACCGCTGAGGAGGCGCG 53

V6(1)* -------TTCCAGGACAGCAAGGCTGACGTGATCGCAAAGTACACTGCTGAGGAGGCTCG 53

************************************** *********** **

TP02_0767(genomic) TACTCTGAAGGCTTACGGCGAGTTGCCTGAAGCGACCAAAATCAACGAAACTGACGTGTA 659

XM_765334(mRNA) TACTCTGAAGGCTTACGGCGAGTTGCCTGAAGCGACCAAAATCAACGAAACTGACGTGTA 421

V1(10) TACTCTGAAGGCTTACGGCGAGTTGCCTGAAGCGACCAAAATCAACGAAACTGACGTGTA 113

V2(1)* TACTCTGAAGGCTTACGGGGAGTTGCCTGAAGCGACCAAAATCAACGAAACTGACGTGTA 113

V3(2) TACTCTGAAGGCTTACGGGGAGTTGCCTGAAGCGACCAAAATCAACGAAACTGACGTGTA 151

V4(1) TACTCTCAAGGCTTACGGAGAATTACCTGAAGCAACTAAAATCAACGAAACAGATGTGTA 151

V5(1)* TACTCTCAAGGCTTACGGAGAATTACCTGAAGCAACTAAAATCAACGAAACAGATGTGTA 113

V6(1)* TACTCTGAAGGCTTACGGTGAGTTGCCTGAAGCGACCAAAATCAACGAAACTGACGTGTA 113

****** *********** ** ** ******** ** ************** ** *****

TP02_0767(genomic) CGACGACGAGGCCGACAACTGCATTGACTTCCAGGACGTATCGTCTGAATCAGAACCTGA 719

XM_765334(mRNA) CGACGACGAGGCCGACAACTGCATTGACTTCCAGGACGTATCGTCTGAATCAGAACCTGA 481

V1(10) CGACGACGAGGCCGACAACTGCATTGACTTCCAGGACGTATCGTCTGAATCAGAACCTGA 173

V2(1)* CGACGACGAGGCCGACAACTGCATTGACTTCCAGGACGTATCCTCTGAATCAGAACCTGA 173

V3(2) CGACGACGAGGCCGACAACTGCATTGACTTCCAGGACGTATCGTCTGAATCAGAACCTGA 211

V4(1) CGACGACCAGGCTGACAACTGCATTGACTTCCCGGACGTTTCCTCCGAGTCCGAACCCGA 211

V5(1)* CGACGACGAGGCTGACAACTGCATTGACTTCCAGGACGTTTCATCCGAGTCAGAACCCGA 173

V6(1)* CGACGACGAGGCCGACAATTGCATTGACTTCCAGGACGTATCCTCTGAATCAGAACCTGA 173

******* **** ***** ************* ****** ** ** ** ** ***** **

TP02_0767(genomic) GGATGAGTCACAAGAGGAGTCGGATTT------------------- 746

XM_765334(mRNA) GGATGAGTCACAAGAGGAGTCGGATTT***CGATATCGATGATTTATAA*** 527

V1(10) GGATGAGTCACAAGAGGAGTCGGATTT------------------- 200

V2(1)* GGATGAGTCACAAGAGGAGTCGGATTT------------------- 200

V3(2) GGATGAGTCACAAGAGGAGTCGGATTT------------------- 238

V4(1) AGATGAATCTCAAGAGGAGTCGGATTT------------------- 238

V5(1)* AGATGAATCTCAAGAGGAATCAGAGTT------------------- 200

V6(1)* GGATGAGTCACAAGAGGAGTCGGATTT------------------- 200

***** ** ******** ** ** **

Primer regions are in bold, underlined and italicized text.

* Partial sequence as defined by only having either the forward or reverse sequence of the allele available for analysis. This sequence is its own variant due to unique nucleotides.

**Table S7b: Tp5 protein variant Clustal alignment**

XP_765334 IRGKMRKRVWVNAGDIILVSLRDFQDSKADVIAKYTAEEARTLKAYGELPEATKINETDV 120

V1(14) -----------NAGDIILVSLRDFQDSKADVIAKYTAEEARTLKAYGELPEATKINETDV 49

V2(1) -----------NAGDIILVSLRDFQDSKADVIAKYTAEEARTLKAYGELPEATKINETDV 49

V3(1* -----------------------FQDSKADVIAKYTAEEARTLKAYGELPEATKINETDV 37

*************************************

XP_765334 YDDEADNCIDFQDVSSESEPEDESQEESDFDIDDL 155

V1(14) YDDEADNCIDFQDVSSESEPEDESQEESD------ 78

V2(1) YDDQADNCIDFPDVSSESEPEDESQEESD------ 78

V3(1)* YDDEADNCIDFQDVSSESEPEDESQEESE------ 66

*** ******* ****************

* Partial sequence as defined by only having either the forward or reverse sequence of the allele available for analysis. This sequence is its own variant due to unique nucleotides.

Eptiope region highlighted in grey.

**Table S8a: Tp6 nucleotide variant Clustal alignment**

TP01_0188(genomic) ATTATTTGTAATGGCTCAGATTCCTGTTGATAAATTCGCTAAATTAGTTACTGGAGCCGG 60

XM_765715(mRNA) ATTATTTGTAATGGCTCAGATTCCTGTTGATAAATTCGCTAAATTAGTTACTGGAGCCGG 0

V1(4) ------------------------------------------------------------ 0

V2(1) ------------------------------------------------------------ 0

V3(1) ------------------------------------------------------------ 0

V4(1) ------------------------------------------------------------ 0

V5(1) ------------------------------------------------------------ 0

V6(1) ------------------------------------------------------------ 0

V7(1) ------------------------------------------------------------ 0

V8(1) ------------------------------------------------------------ 0

V9(2) ------------------------------------------------------------ 0

V10(1) ------------------------------------------------------------ 0

V11(1) ------------------------------------------------------------ 0

V12(2) ------------------------------------------------------------ 0

V13(6) ------------------------------------------------------------ 0

TP01_0188(genomic) CTCTGCTCTCTTATTATTCGGTTCAGGTGCCTGGCTTGTCAATTCCAGTTTATACGATGG 120

XM_765715(mRNA) CTCTGCTCTCTTATTATTCGGTTCAGGTGCCTGGCTTGTCAATTCCAGTTTATACGATG- 120

V1(4) -TCTGCTCTCTTATTATTCGGTTCAGGTGCCTGGCTTGTCAATTCCAGTTTATACGATGG 59

V2(1) -TCCGCTCTCTTATTATTCGGTTCAGGTGCCTGGCTTGTTAATTCCAGTTTATACGATGG 59

V3(1) -TCCGCTCTCTTATTATTCGGTTCAGGTGCCTGGCTTGTTAATTCCAGTTTATACGATGG 59

V4(1) -TCCGCTCTCTTATTATTCGGTTCAGGTGCCTGGCTTGTTAATTCCAGTTTATACGACGG 59

V5(1) ------------------------------------------------------------ 0

V6(1) --CCGCTCTCTTATTATTCGGTTCAGGTGCCTGGCTTGTCAATTCCAGTTTATACGATGG 58

V7(1) ----GCTCTCTTATTATTCGGTTCAGGTGCCTGGCTTGTCAATTCCAGTTTATACGATGG 56

V8(1) -TCCGCTCTCTTATTATTCGGTTCAGGTGCCTGGCTTGTCAATTCCAGTTTATACGATGG 59

V9(2) ----GCTCTCTTATTATTCGGTTCAGGTGCCTGGCTTGTTAATTCCAGTTTATACGATGG 56

V10(1) -TCCGCTCTCTTATTATTCGGTTCAGGTGCCTGGCTTGTTAATTCCAGTTTATACGATGG 59

V11(1) -TCTGCTCTCTTATTATTCGGTTCAGGTGCCTGGCTTGTCAATTCCAGTTTATACGATGG 59

V12(2) -TCCGCTCTCTTATTATTCGGTTCAGGTGCCTGGCTTGTTAATTCCAGTTTATACGATGG 59

V13(6) -TCTGCTCTCTTATTATTCGGTTCAGGTGCCTGGCTTGTCAATTCCAGTTTATACGATGG 59

TP01_0188(genomic) TAAGATTCTACTCTACTTCCACTTTATATTAGCTTTATTTTATTATATAATATATATAGA 180

XM_765715(mRNA) ------------------------------------------------------------ 120

V1(4) TAAGATTCTACTCTACTTCCACTTTATATTAGCTTTATTTTATTATATAATATATATAGA 119

V2(1) TAAGATTCTACTCGACTTCCACTTTATATTAGCTTTATTTTATTATATAATATATATAAA 119

V3(1) TAAGATTCTACTCGACTTCCACTTTATATTAGCTTTATTTTATTATTTTATATATATAAA 119

V4(1) TAAGATTCTACTCGACTTCCACTTTATATTAGCTTTATTTTATTATATAATATATATATA 119

V5(1) -----------------------------------TATTTTATTATATAATATATATAAA 25

V6(1) TAAGATTCTACTCGACTTCCACTTTATATTAGCTTTATTTTATTATATAATATATATAAA 118

V7(1) TAAGATTCTATTCGACTTCCACTTTATATTAGCTTTATTTTATTATATAATATATATAAA 116

V8(1) TAAGATTCTATTCGACTTCCACTTTATATTAGCTTTATTTTATTATTTTATATATATAAA 119

V9(2) TAAGATTCTACTCGACTTCCACTTTATATTAGCTTTATTTTATTATTTTATATATATAAA 116

V10(1) TAAGATTCTACTCGACTTCCACTTTATATTAGCTTTATTTTATTATTTTATATATATAAA 119

V11(1) TAAGATTCTACTCGACTTCCACTTTATATTAGCTTTATTTTATTATATAATATATATAAA 119

V12(2) TAAGATTCTACTCGACTTCCACTTTATATTAGCTTTATTTTATTATATAATATATATAAA 119

V13(6) TAAGATTCTACTCGACTTCCACTTTATATTAGCTTTATTTTATTATATAATATATATAAA 119

TP01_0188(genomic) ATTGTAAAAACAATCTTTAAAAACCTTTATCATTAATTTTTATTGATTTTTTTATTGT 238

XM_765715(mRNA) ---------------------------------------------------------- 120

V1(4) ATTGTAAAAACAATCTTTAAAAACCTTTATCATTAATTTTTATTGATTTTTTTATTGT 177

V2(1) ATTGTAAAAATACCCTTTTAAATCCTTTATCATTGATTTTTATTGATTTTTTTATTGT 177

V3(1) ATTGTAAAAATAATCTTTAAAAACCTTTATCATTAATTTTTATTGATTTTTTTATTGT 177

V4(1) ATTGTAAAAATACCCTTTTAAATCCTTTATCATTAATTTTTATTGATTTTTTTATTGT 177

V5(1) ATTGTAAAAATACCCTTTTAAATCCTTTATCATTGATTTTTATTGATTTTTTTATTGT 83

V6(1) ATTGTAAAAATACCCTTTTAAATCCTTTATCATTGATTTTTATTGATTTTTTTATTGT 176

V7(1) ATTGTAAAAATACCCTTTTAAATCCTTTATCATTAATTTTTATTGATTTTTTTATTGT 174

V8(1) ATTGTAAAAATAATCTTTAAAAACCTTTATCATTAATTTTTATTGATTTTTTTATTGT 177

V9(2) ATTGTAAAAATAATCTTTAAAAACCTTTATCATTAATTTTTATTGATTTTTTTATTGT 174

V10(1) ATTGTAAAAATAATCTTTAAAAACCTTTATCATTAATTTTTATTGATTTTTTTATTGT 177

V11(1) ATTGTAAAAATACCCTTTTAAATCCTTTATCATTGATTTTTATTGATTTTTTTATTGT 177

V12(2) ATTGTAAAAATACCCTTTTAAATCCTTTATCATTGATTTTTATTGATTTTTTTATTGT 177

V13(6) ATTGTAAAAATACCCTTTTAAATCCTTTATCATTGATTTTTATTGATTTTTTTATTGT 177

TP01_0188(genomic) AGTTATATTGAACAGTAATCATTTTTAGAATGATTTTTAATTTGTTAAATATTTTATAAA 298

XM_765715(mRNA) ------------------------------------------------------------ 120

V1(4) AGTTATATTGAACAGTAATCATTTTTAGAATGATTTTTAATTTGTTAAATATTTTATAAA 237

V2(1) AGTTATATTGAACAGTAATCATTTTTAGAATGATTTTTAATTTGTTAAATATTTTATAAA 237

V3(1) AGTTATATTGAACAGTAATCATTTTTAGAATGATTTTTAATTTGTTAAATATTTTATAAA 237

V4(1) AAATATATTGAACAGTAATGATTTTTAGAATGATTTTTAATTTGTTAAATATTTTATAAA 237

V5(1) AGTTATATTGAACAGTAATCATTTTTAGAATGATTTTTAATTTGTTAAATATTTTATAAA 143

V6(1) AGTTATATTGAACAGTAATCATTTTTAGAATGATTTTTAATTTGTTAAATATTTTATAAA 236

V7(1) AGTTATATTGAACAGTAATCATTTTTAGAATGATTTTTAATTTGTTAAATATTTTATAAA 234

V8(1) AGTTATATTGAACAGTAATCATTTTTAGAATGACTTTTAATTTGTTAAATATTTTATAAA 237

V9(2) AGTTATATTGAACAGTAATCATTTTTAGAATGATTTTTAATTTGTTAAATATTTTATAAA 234

V10(1) AGTTATATTGAACAGTAATCATTTTTAGAATGATTTTTAATTTGTTAAATATTTTATAAA 237

V11(1) AGTTATATTGAACAGTAATCATTTTTAGAATGATTTTTAATTTGTTAAATATTTTATAAA 237

V12(2) AGTTATATTGAACAGTAATCATTTTTAGAATGATTTTTAATTTGTTAAATATTTTATAAA 237

V13(6) AGTTATATTGAACAGTAATCATTTTTAGAATGATTTTTAATTTGTTAAATATTTTATAAA 237

TP01_0188(genomic) TTTTACTATTTAATGTCTCTTTAATGTTGAATTAGTTGGAGCTGGGCATAGAGCTGTTGT 358

XM_765715(mRNA) -----------------------------------TTGGAGCTGGGCATAGAGCTGTTGT 144

V1(4) TTTTACTATTTAATGTCTCTTTAATGTTGAATTAGTTGGAGCTGGGCATAGAGCTGTTGT 297

V2(1) TTTTACGATTTAATATCTCTTTAATGTTGAATTAGTTGGAGCTGGGCATAGAGCTGTTGT 297

V3(1) TTTTACGATTTAATATCTCTTTAATGTTGAATTAGTTGGAGCTGGGCATAGAGCTGTCGT 297

V4(1) TTTTACTATTTAATGTCTCTTTAATGTTGAATTAGTTGGAGCTGGGCATAGAGCTGTCGT 297

V5(1) TTTTACGATTTAATATCTCTTTAATGTTGAATTAGTTGGAGCTGGGCATAGAGCTGTTGT 203

V6(1) TTTTACGATTTAATATCTCTTTAATGTTGAATTAGTTGGAGCTGGGCATAGAGCTGTCGT 296

V7(1) TTTTACTATTTAATATCTCTTTAATGTTGAATTAGTTGGAGCTGGGCATAGAGCTGTTGT 294

V8(1) TTTTACTATTTAATGTCTCTTTAATGTTGAATTAGTTGGAGCTGGGCATAGAGCTGTCGT 297

V9(2) TTTTACGATTTAATATCTCTTTAATGTTGAATTAGTTGGAGCTGGGCATAGAGCTGTCGT 294

V10(1) TTTTACGATTTAATATCTCTTTAATGTTGAATTAGTTGGAGCTGGGCATAGAGCTGTCGT 297

V11(1) TTTTACGATTTAATATCTCTTTAATGTTGAATTAGTTGGAGCTGGGCATAGAGCTGTCGT 297

V12(2) TTTTACGATTTAATATCTCTTTAATGTTGAATTAGTTGGAGCTGGGCATAGAGCTGTTGT 297

V13(6) TTTTACGATTTAATATCTCTTTAATGTTGAATTAGTTGGAGCTGGGCATAGAGCTGTCGT 297

*************************

TP01_0188(genomic) ATATAACCGTATCACTGGAATAAGTGAGACTACACATGGAGAAGGAACGCACTTCATAAT 418

XM_765715(mRNA) ATATAACCGTATCACTGGAATAAGTGAGACTACACATGGAGAAGGAACGCACTTCATAAT 204

V1(4) ATATAACCGTATCACTGGAATAAGTGAGACTACACATGGAGAAGGAACGCACTTCATAAT 357

V2(1) ATATAACCGTATCACTGGAATAAGTGAGACTACACATGGAGAAGGAACCCACTTCATAAT 357

V3(1) ATATAACCGTATCACTGGAATAAGTGAGACTACACATGGAGAAGGAACGCACTTCATAAT 357

V4(1) ATATAACCGTATCACTGGAATAAGTGAGACTACACATGGAGAAGGAACCCACTTCATAAT 357

V5(1) ATATAACCGTATCACTGGAATAAGTGAGACTACACATGGAGAAGGAACCCACTTCATAAT 263

V6(1) ATATAACCGTATCACTGGAATAAGTGAGACTACACATGGAGAAGGAACCCACTTCATAAT 356

V7(1) ATATAACCGTATCACTGGAATAAGTGAGACTACACATGGAGAAGGAACGCACTTCATAAT 354

V8(1) ATATAACCGTATCACTGGAATAAGTGAGACTACACATGGAGAAGGAACGCACTTCATAAT 357

V9(2) ATATAACCGTATCACTGGAATAAGTGAGACTACACATGGAGAAGGAACGCACTTCATAAT 354

V10(1) ATATAACCGTATCACTGGAATAAGTGAGACTACACATGGAGAAGGAACGCACTTCATAAT 357

V11(1) ATATAACCGTATCACTGGAATAAGTGAGACTACACATGGAGAAGGAACCCACTTCATAAT 357

V12(2) ATATAACCGTATCACTGGAATAAGTGAGACTACACATGGAGAAGGAACCCACTTCATAAT 357

V13(6) ATATAACCGTATCACTGGAATAAGTGAGACTACACATGGAGAAGGAACCCACTTCATAAT 357

************************************************ ***********

TP01_0188(genomic) TCCCTGGCTAGAACGTCCAATAATTTACGATGTGAGGACTCGTCCTAGGACTCTGATGTC 478

XM_765715(mRNA) TCCCTGGCTAGAACGTCCAATAATTTACGATGTGAGGACTCGTCCTAGGACTCTGATGTC 264

V1(4) TCCCTGGCTAGAACGTCCAATAATTTACGATGTGAGGACTCGTCCTAGGACTCTGATGTC 417

V2(1) TCCCTGGCTAGAACGTCCAATAATTTACGATGTGAGGACTCGTCCTAGGACTCTGATGTC 417

V3(1) TCCCTGGTTAGAGCGTCCAATAATTTACGATGTGAGGACTCGTCCTAGGACTCTGATGTC 417

V4(1) TCCCTGGTTAGAGCGTCCAATAATTTACGATGTGAGGACTCGTCCTAGGACTCTGATGTC 417

V5(1) TCCCTGGTTAGAGCGTCCAATAATTTACGATGTGAGGACTCGTCCTAGGACTCTGATGTC 323

V6(1) TCCATGGTTAGAGCGTCCAATAATTTACGATGTGAGGACTCGTCCTAGGACTCTGATGTC 416

V7(1) TCCATGGTTAGAGCGTCCAATAATTTACGATGTGAGGACTCGTCCTAGGACTCTGATGTC 414

V8(1) TCCCTGGTTAGAGCGTCCAATAATTTACGATGTGAGGACTCGTCCTAGGACTCTGATGTC 417

V9(2) TCCCTGGTTAGAGCGTCCAATAATTTACGATGTGAGGACTCGTCCTAGGACTCTGATGTC 414

V10(1) TCCCTGGTTAGAGCGTCCAATAATTTACGATGTGAGGACTCGTCCTAGGACTCTGATGTC 417

V11(1) TCCATGGTTAGAGCGTCCAATAATTTACGATGTGAGGACTCGTCCTAGGACTCTGATGTC 417

V12(2) TCCCTGGCTAGAACGTCCAATAATTTACGATGTGAGGACTCGTCCTAGGACTCTGATGTC 417

V13(6) TCCATGGTTAGAGCGTCCAATAATTTACGATGTGAGGACTCGTCCTAGGACTCTGATGTC 417

*** *** **** ***********************************************

TP01_0188(genomic) TCTCACCGGAAGCCGTGACTTGCAGATGGTTAACATCACCTGCCGTGTGTTGTCTCGTCC 538

XM_765715(mRNA) TCTCACCGGAAGCCGTGACTTGCAGATGGTTAACATCACCTGCCGTGTGTTGTCTCGTCC 324

V1(4) TCTCACCGGAAGCCGTGACTTGCAGATGGTTAACATCACCTGCCGTGTGTTGTCTCGTCC 477

V2(1) TCTCACCGGAAGCCGTGACTTGCAGATGGTTAACATCACCTGCCGTGTGTTGTCTCGTCC 477

V3(1) TCTCACCGGAAGCCGTGACTTGCAGATGGTTAACATCACCTGCCGTGTGTTGTCTCGTCC 477

V4(1) TCTCACCGGAAGCCGTGACTTGCAGATGGTTAACATCACCTGCCGTGTGTTGTCTCGTCC 477

V5(1) TCTCACCGGAAGCCGTGACTTGCAGATGGTTAACATCACCTGCCGTGTGTTGTCTCGTCC 383

V6(1) TCTCACCGGAAGCCGTGACTTGCAGATGGTTAACATCACCTGCCGTGTGTTGTCTCGTCC 476

V7(1) TCTCACCGGAAGCCGTGACTTGCAGATGGTTAACATCACCTGCCGTGTGTTGTCTCGTCC 474

V8(1) TCTCACCGGAAGCCGTGACTTGCAGATGGTTAACATCACGTGCCGTGTGTTGTCTCGTCC 477

V9(2) TCTCACCGGAAGCCGTGACTTGCAGATGGTTAACATCACCTGCCGTGTGTTGTCTCGTCC 474

V10(1) TCTCACCGGAAGCCGTGACTTGCAGATGGTTAACATCACCTGCCGTGTGTTGTCTCGTCC 477

V11(1) TCTCACCGGAAGCCGTGACTTGCAGATGGTTAACATCACCTGCCGTGTGTTGTCTCGTCC 477

V12(2) TCTCACCGGAAGCCGTGACTTGCAGATGGTTAACATCACCTGCCGTGTGTTGTCTCGTCC 477

V13(6) TCTCACCGGAAGCCGTGACTTGCAGATGGTTAACATCACCTGCCGTGTGTTGTCTCGTCC 477

*************************************** ********************

TP01_0188(genomic) CGATGAGCGCAGACTCAGGGATATTTACAGGCACTTGGGCAAAGATTACGACGAGCGAGT 598

XM_765715(mRNA) CGATGAGCGCAGACTCAGGGATATTTACAGGCACTTGGGCAAAGATTACGACGAGCGAGT 384

V1(4) CGATGAGCGCAGACTCAGGGATATTTACAGGCACTTGGGCAAAGATTACGACGAGCGAGT 537

V2(1) CGATGAGCGCAGACTCAGGGATATTTACAGGCACTTGGGGAAAGACTATGACGAGCGAGT 537

V3(1) CGATGAGCGCAGACTCAGGGATATTTACAGGCACTTGGGGAAAGACTATGACGAGCGAGT 537

V4(1) CGATGAGCGCAGACTCAGGGATATTTACAGGCACTTGGGGAAAGACTATGACGAGCGAGT 537

V5(1) CGATGAGCGCAGACTCAGGGATATTTACAGGCACTTGGGGAAAGATTATGACGAGCGAGT 443

V6(1) CGATGAGCGCAGACTCAGGGATATTTACAGGCACTTGGGGAAAGACTATGACGAGCGAGT 536

V7(1) TGATGAGCGCAGACTCAGGGATATTTACAGGCACTTGGGGAAAGACTATGACGAGCGAGT 534

V8(1) CGATGAGCGCAGACTCAGGGATATTTACAGGCACTTGGGGAAAGACTATGACGAGCGAGT 537

V9(2) CGATGAGCGCAGACTCAGGGATATTTACAGGCACTTGGGGAAAGACTATGACGAGCGAGT 534

V10(1) CGATGAGCGCAGACTCAGGGATATTTACAGGCACTTGGGGAAAGACTATGACGAGCGAGT 537

V11(1) CGATGAGCGCAGACTCAGGGATATTTACAGGCACTTGGGGAAAGACTATGACGAGCGAGT 537

V12(2) CGATGAGCGCAGACTCAGGGATATTTACAGGCACTTGGGGAAAGACTATGACGAGCGAGT 537

V13(6) CGATGAGCGCAGACTCAGGGATATTTACAGGCACTTGGGGAAAGACTATGACGAGCGAGT 537

************************************** ***** ** ***********

TP01_0188(genomic) CCTGCCTTCAATAATAAACGAGGTTCTGAAGAGTATTGTGGCCCAGTACAACGCCTCTCA 658

XM_765715(mRNA) CCTGCCTTCAATAATAAACGAGGTTCTGAAGAGTATTGTGGCCCAGTACAACGCCTCTCA 444

V1(4) CCTGCCTTCAATAATAAACGAGGTTCTGAAGAGTATTGTGGCCCAGTACAACGCCTCTCA 597

V2(1) CCTGCCTTCAATAATAAACGAGGTTCTGAAGAGTATTGTGGCCCAGTACAACGCCTCTCA 597

V3(1) CCTGCCTTCAATAATAAACGAGGTTCTGAAGAGTATAGTTGCCCAGTACAACGCCTCTCA 597

V4(1) CCTGCCTTCAATAATAAACGAGGTTCTGAAGAGTATTGTGGCCCAGTACAACGCCTCTCA 597

V5(1) CCTGCCTTCAATAATAAACGAGGTTCTGAAGAGTATTGTGGCCCAGTACAACGCCTCTCA 503

V6(1) CCTGCCTTCAATAATAAACGAGGTTCTGAAGAGTATTGTGGCCCAGTACAACGCCTCTCA 596

V7(1) CCTGCCTTCAATAATAAACGAGGTTCTGAAGAGTATTGTGGCCCAGTACAACGCCTCTCA 594

V8(1) CCTGCCTTCAATAATAAACGAGGTTCTGAAGAGTATAGTTGCCCAGTACAACGCCTCTCA 597

V9(2) CCTGCCTTCAATAATAAACGAGGTTCTGAAGAGTATAGTTGCCCAGTACAACGCCTCTCA 594

V10(1) CCTGCCTTCAATAATAAACGAGGTTCTGAAGAGTATAGTTGCCCAGTACAACGCCTCTCA 597

V11(1) CCTGCCTTCAATAATAAACGAGGTTCTGAAGAGTATTGTGGCCCAGTACAACGCCTCTCA 597

V12(2) CCTGCCTTCAATAATAAACGAGGTTCTGAAGAGTATTGTGGCCCAGTACAACGCCTCTCA 597

V13(6) CCTGCCTTCAATAATAAACGAGGTTCTGAAGAGTATTGTGGCCCAGTACAACGCCTCTCA 597

************************************ ** ********************

TP01_0188(genomic) GCTCATTACTCAGAGAGAAAGAGTTAGCAAAGCAGTCAGGGACCAGCTGGTGAACAGGGC 718

XM_765715(mRNA) GCTCATTACTCAGAGAGAAAGAGTTAGCAAAGCAGTCAGGGACCAGCTGGTGAACAGGGC 504

V1(4) GCTCATTACTCAGAGAGAAAGAGTTAGCAAAGCAGTCAGGGACCAGCTGGTGAACAGGGC 657

V2(1) GCTCATTACTCAGAGAGAAAGAGTTAGCAAAGCAGTCAGGGACCAGCTGGTGAACAGGGC 657

V3(1) GCTCATTACTCAGAGAGAAAGGGTTAGCAAAGCAGTCAGGGACCAGCTGGTGAACAGGGC 657

V4(1) GCTCATTACTCAGAGAGAAAGAGTTAGCAAAGCAGTCAGGGACCAGCTGGTGAACAGGGC 657

V5(1) GCTCATTACTCAGAGAGAAAGAGTTAGCAAAGCAGTCAGGGACCAGCTGGTGAACAGGGC 563

V6(1) GCTCATTACTCAGAGAGAAAGAGTTAGCAAAGCAGTCAGGGACCAGCTGGTGAACAGGGC 656

V7(1) GCTCATTACTCAGAGAGAAAGAGTTAGCAAAGCAGTCAGGGACCAGCTGGTGAACAGGGC 654

V8(1) GCTCATTACTCAGAGAGAAAGGGTTAGCAAAGCAGTCAGGGACCAGCTGGTGAACAGGGC 657

V9(2) GCTCATTACTCAGAGAGAAAGGGTTAGCAAAGCAGTCAGGGACCAGCTGGTGAACAGGGC 654

V10(1) GCTCATTACTCAGAGAGAAAGGGTTAGCAAAGCAGTCAGGGACCAGCTGGTGAACAGGGC 657

V11(1) GCTCATTACTCAGAGAGAAAGAGTTAGCAAAGCAGTCAGGGACCAGCTGGTGAACAGGGC 657

V12(2) GCTCATTACTCAGAGAGAAAGAGTTAGCAAAGCAGTCAGGGACCAGCTGGTGAACAGGGC 657

V13(6) GCTCATTACTCAGAGAGAAAGAGTTAGCAAAGCAGTCAGGGACCAGCTGGTGAACAGGGC 657

********************* **************************************

TP01_0188(genomic) CAGGGACTTTAATATTCTTCTCGATGATGTCTCCTTAACCCACTTAAGCTTCAGTCCTGA 778

XM_765715(mRNA) CAGGGACTTTAATATTCTTCTCGATGATGTCTCCTTAACCCACTTAAGCTTCAGTCCTGA 564

V1(4) CAGGGACTTTAATATTCTTCTCGATGATGTCTCCTTAACCCACTTAAGCTTCAGTCCTGA 717

V2(1) CAGGGACTTCAACATTCTTCTTGATGATGTCTCCTTAACCCACTTAAGCTTCAGTCCTGA 717

V3(1) CAGGGACTTTAACATTCTTCTTGATGATGTCTCCTTAACCCACTTAAGCTTCAGTCCTGA 717

V4(1) CAGGGACTTTAATATTCTTCTTGATGATGTCTCCTTAACCCACTTAAGCTTCAGTCCTGA 717

V5(1) CAGGGACTTTAACATTCTTCTTGATGATGTCTCCTTAACCCACTTAAGCTTCAGTCCTGA 623

V6(1) CAGGGACTTTAACATTCTTCTTGATGATGTCTCCTTAACCCACTTAAGCTTCAGTCCTGA 716

V7(1) CAGGGACTTTAACATTCTTCTTGATGATGTCTCCTTAACCCACTTAAGCTTCAGTCCTGA 714

V8(1) CAGGGACTTTAACATTCTTCTTGATGATGTCTCCTTAACCCACTTAAGCTTCAGTCCTGA 717

V9(2) CAGGGACTTTAACATTCTTCTTGATGATGTCTCCTTAACCCACTTAAGCTTCAGTCCTGA 714

V10(1) CAGGGACTTTAACATTCTTCTTGATGATGTCTCCTTAACCCACTTAAGCTTCAGTCCTGA 717

V11(1) CAGGGACTTTAATATTCTTCTTGATGATGTCTCCTTAACCCACTTAAGCTTCAGTCCTGA 717

V12(2) CAGGGACTTCAACATTCTTCTTGATGATGTCTCCTTAACCCACTTAAGCTTCAGTCCTGA 717

V13(6) CAGGGACTTTAATATTCTTCTTGATGATGTCTCCTTAACCCACTTAAGCTTCAGTCCTGA 717

********* ** ******** **************************************

TP01_0188(genomic) ATATGAAAAGGCTGTAGAGGCTAAACAAGTAGCTCAACAGCAAGCTGAACGCAGTAAATA 838

XM_765715(mRNA) ATATGAAAAGGCTGTAGAGGCTAAACAAGTAGCTCAACAGCAAGCTGAACGCAGTAAATA 624

V1(4) ATATGAAAAGGCTGTAGAGGCTAAACAAGTAGCTCAACAGCAAGCTGAACGCAGTAAATA 777

V2(1) ATATGAAAAGGCTGTAGAGGCTAAACAAGTAGCTCAACAGCAAGCTGAACGCAGTAAATA 777

V3(1) ATATGAAAAGGCTGTAGAGGCTAAACAAGTAGCTCAACAGCAAGCTGAACGCAGTAAATA 777

V4(1) ATATGAAAAGGCTGTAGAGGCTAAACAAGTAGCTCAACAGCAAGCTGAACGCAGTAAGTA 777

V5(1) ATATGAAAAGGCTGTAGAGGCTAAACAAGTAGCTCAACAGCAAGCTGAACGTAGTAAATA 683

V6(1) ATATGAAAAGGCTGTAGAGGCTAAACAAGTAGCTCAACAGCAAGCTGAACGTAGTAAATA 776

V7(1) ATATGAAAAGGCTGTAGAGGCTAAACAAGTAGCTCAACAGCAAGCTGAACGCAGTAAATA 774

V8(1) ATATGAAAAGGCTGTAGAGGCTAAACAAGTAGCTCAACAGCAAGCTGAACGCAGTAAATA 777

V9(2) ATATGAAAAGGCTGTAGAGGCTAAACAAGTAGCTCAACAGCAAGCTGAACGCAGTAAATA 774

V10(1) ATATGAAAAGGCTGTAGAGGCTAAACAAGTAGCTCAACAGCAAGCTGAACGCAGTAAATA 777

V11(1) ATATGAAAAGGCTGTAGAGGCTAAACAAGTAGCTCAGCAGCAAGCTGAACGCAGTAAATA 777

V12(2) ATATGAAAAGGCTGTAGAGGCTAAACAAGTAGCTCAACAGCAAGCTGAACGCAGTAAATA 777

V13(6) ATATGAAAAGGCTGTAGAGGCTAAACAAGTAGCTCAGCAGCAAGCTGAACGCAGTAAATA 777

************************************ ************** ***** **

TP01_0188(genomic) TATAGTGTTGAAGGCTCAGGAGGAGAAGAAATCGACGATAATTAAGGCTCAGGGAGAGTC 898

XM_765715(mRNA) TATAGTGTTGAAGGCTCAGGAGGAGAAGAAATCGACGATAATTAAGGCTCAGGGAGAGTC 684

V1(4) TATAGTGTTGAAGGCTCAGGAGGAGAAGAAATCGACGATAATTAAGGCTCAGGGAGAGTC 837

V2(1) TATAGTGTTGAAGGCTCAGGAGGAGAAGAAATC--------------------------- 810

V3(1) TATAGTGTTGAAGGCTCAGGAGGAGAAGAAATCGACGATAATTAAGGCTCAGGGAGATTC 837

V4(1) TATAGTGTTGAAGGCTCAGGAGGAGAAGAAATCGACGATAATTAAGGCTCAGGGAGAGTC 837

V5(1) TATAGTGTTGAAGGCTCAGGAGGAGAAGAAATCGACGATAATTAAGGCTCAGGGAGAGTC 743

V6(1) TATAGTGTTGAAGGCTCAGGAGGAGAAGAAATCGACGATAATTAAGGCTCAGGGAGAGTC 836

V7(1) TATAGTGTTGAAGGCTCAGGAGGAGAAGAAATCGACGATAATTAAGGCTCAGGGAGAGTC 834

V8(1) TATAGTGTTGAAGGCTCAGGAGGAGAAGAAATCGACGATAATTAAGGCTCAGGGAGAGTC 837

V9(2) TATAGTGTTGAAGGCTCAGGAGGAGAAGAAATCGACGATAATTAAGGCTCAGGGAGAGTC 834

V10(1) TATAGTGTTGAAGGCTCAGGAGGAGAAGAAATCGACGATAATTAAGGCTCAGGGAGATTC 837

V11(1) TATAGTGTTGAAGGCTCAGGAGGAGAAGAAATCGACGATAATTAAGGCTCAGGGAGAGTC 837

V12(2) TATAGTGTTGAAGGCTCAGGAGGAGAAGAAATC--------------------------- 810

V13(6) TATAGTGTTGAAGGCTCAGGAGGAGAAGAAATCGACGATAATTAAGGCTCAGGGAGAGTC 837

*********************************

TP01_0188(genomic) TGAGGCTGCAAGGCTTATTGGAAGTGCAATTAAGGATAACCCTGCCTTTATTACGCTTCG 958

XM_765715(mRNA) TGAGGCTGCAAGGCTTATTGGAAGTGCAATTAAGGATAACCCTGCCTTTATTACGCTTCG 744

V1(4) TGAGGCTGCAAGGCTTATTGGAAGTGCAATTAAGGATAACCCTGCCTTTATTACGCTTCG 897

V2(1) ------------------------------------------------------------ 810

V3(1) TGAGGCTGCAAGGCTTATTGGAAGTGCAATTAAGGATAACCCTGCCTTTATTACGCTTCG 897

V4(1) TGAGGCTGCAAGGCTTATTGGAAGTGCAATTAAGGATAACCCTGCCTTTATTACGCTTCG 897

V5(1) TGAGGCTGCAAGGCTTATTGGAAGTGCAATTAAGGATAACCC------------------ 785

V6(1) TGAGGCTGCAAGGCTTATTGGAAGTGCAATTAAGGATAACCCTGCCTTTATTACGCTTCG 896

V7(1) TGAGGCTGCAAGG----------------------------------------------- 847

V8(1) TGAGGCTGCAAGGCTTATTGGAAGTGCAATTAAGGATAACCCTGCCTTTATTACGCTTCG 897

V9(2) TGAGGCTGCAAGGCTTATTGGAAGTGCAATTAAGGATAACCCTGCCTTTATTACGCTTCG 894

V10(1) TGAGGCTGCAAGGCTTATTGGAAGTGCAATTAAGGATAACCCTGCCTTTATTACGCTTCG 897

V11(1) TGAGGCTGCAAGGCTTATTGGAAGTGCAATTAAGGATAACCCTGCCTTTATTACGCTTCG 897

V12(2) ------------------------------------------------------------ 810

V13(6) TGAGGCTGCAAGGCTTATTGGAAGTGCAATTAAGGATAACCCTGCCTTTATTACGCTTCG 897

TP01_0188(genomic) GAGAATTGAAACCGCTAAGGAAGTGGCTAACATTCTCTCCAAATCGCAGAATAAAATCAT 1018

XM_765715(mRNA) GAGAATTGAAACCGCTAAGGAAGTGGCTAACATTCTCTCCAAATCGCAGAATAAAATCAT 804

V1(4) GAGAATTGAAACCGCTAAGGAAGTGGCTAACATT-------------------------- 931

V2(1) ------------------------------------------------------------ 810

V3(1) GAGAATTGAAACCGCTAAGGAAGTGGCTAACATT-------------------------- 931

V4(1) GAGAATTGAAACCGCTAAGGAAGTGGCTAACATT-------------------------- 931

V5(1) ------------------------------------------------------------ 785

V6(1) GAGAATTGAAACCGCTAAGGAAGTGGCTAACATT-------------------------- 930

V7(1) ------------------------------------------------------------ 847

V8(1) GAGAATTGAAACCGCTAAGGAAGTGGCTAACATT-------------------------- 931

V9(2) GAGAATTGAAACCGCTAAGGAAGTGGCTAACATT-------------------------- 928

V10(1) GAGAATTGAAACCGCTAAGGAAGTGGCTAACATT-------------------------- 931

V11(1) GAGAATTGAAACCGCTAAGGAAGTGGCTAACATT-------------------------- 931

V12(2) ------------------------------------------------------------ 810

V13(6) GAGAATTGAAACCGCTAAGGAAGTGGCTAACATT-------------------------- 931

TP01_0188(genomic) GCTCAATAGTAATACTCTCTTACTCTCAACTGATAAATAATATTATCAATCCTCCAATAT 1078

XM_765715(mRNA) GCTCAATAGTAATACTCTCTTACTCTCAACTGATAAATAATATTATCAATCCTCCAATAT 864

V1(4) ------------------------------------------------------------ 931

V2(1) ------------------------------------------------------------ 810

V3(1) ------------------------------------------------------------ 931

V4(1) ------------------------------------------------------------ 931

V5(1) ------------------------------------------------------------ 785

V6(1) ------------------------------------------------------------ 930

V7(1) ------------------------------------------------------------ 847

V8(1) ------------------------------------------------------------ 931

V9(2) ------------------------------------------------------------ 928

V10(1) ------------------------------------------------------------ 931

V11(1) ------------------------------------------------------------ 931

V12(2) ------------------------------------------------------------ 810

V13(6) ------------------------------------------------------------ 931

TP01_0188(genomic) GCTATTATAT 1088

XM_765715(mRNA) GCTATTATAT 874

V1(4) ---------- 931

V2(1) ---------- 810

V3(1) ---------- 931

V4(1) ---------- 931

V5(1) ---------- 785

V6(1) ---------- 930

V7(1) ---------- 847

V8(1) ---------- 931

V9(2) ---------- 928

V10(1) ---------- 931

V11(1) ---------- 931

V12(2) ---------- 810

V13(6) ---------- 931

Primer regions are in bold, underlined and italicized text.

^1^ Include X.1 variants.

**Table S8b: Tp6 protein variant Clustal alignment**

XP_765715 MAQIPVDKFAKLVTGAGSALLLFGSGAWLVNSSLYDVGAGHRAVVYNRITGISETTHGEG 60

V1(22)^1^ ------------------------------------VGAGHRAVVYNRITGISETTHGEG 24

V2(1) ------------------------------------VGAGHRAVVYNRITGISETTHGEG 24

************************

XP_765715 THFIIPWLERPIIYDVRTRPRTLMSLTGSRDLQMVNITCRVLSRPDERRLRDIYRHLGKD 120

V1(22) THFIIPWLERPIIYDVRTRPRTLMSLTGSRDLQMVNITCRVLSRPDERRLRDIYRHLGKD 84

V2(1) THFIIPWLERPIIYDVRTRPRTLMSLTGSRDLQMVNITCRVLSRPDERRLRDIYRHLGKD 84

************************************************************

XP_765715 YDERVLPSIINEVLKSIVAQYNASQLITQRERVSKAVRDQLVNRARDFNILLDDVSLTHL 180

V1(22) YDERVLPSIINEVLKSIVAQYNASQLITQRERVSKAVRDQLVNRARDFNILLDDVSLTHL 144

V2(1) YDERVLPSIINEVLKSIVAQYNASQLITQRERVSKAVRDQLVNRARDFNILLDDVSLTHL 144

************************************************************

XP_765715 SFSPEYEKAVEAKQVAQQQAERSKYIVLKAQEEKKSTIIKAQGESEAARLIGSAIKDNPA 240

V1(22) SFSPEYEKAVEAKQVAQQQAERSKYIVLKAQEEKKSTIIKAQGESEAARLIGSAIKDNPA 204

V2(1) SFSPEYEKAVEAKQVAQQQAERSKYIVLKAQEEKKSTIIKAQGDSEAARLIGSAIKDNPA 204

******************************************* ****************

XP_765715 FITLRRIETAKEVANILSKSQNKIMLNSNTLLLSTDK 277

V1(22) FITLRRIETAKEVANI--------------------- 220

V2(1) FITLRRIETAKEVANI--------------------- 220

****************

^1^ Include X.1 variants.

Eptiope sequence unknown.

**Table S9a: Tp7 nucleotide variant Clustal alignment**

TP02_0244(genomic) CATTC***ATGGAGGCACTGCAAGCAGGC***TCGGACATGTCAATGATCGGACAGTTTGGTGTCG 720

XM_759717(mRNA) CATTCATGGAGGCACTGCAAGCAGGCTCGGACATGTCAATGATCGGACAGTTTGGTGTCG 490

V1(2) ------------------------------------------------------------ 0

V2(4)^1^ ------------------------------------------------------------ 0

V3(1) ------------------------------------------------------------ 0

V4(1) ------------------------------------------------------------ 0

V5(2) ------------------------------------------------------------ 0

V6(1) ------------------------------------------------------------ 0

V7(7) ------------------------------------------------------------ 0

V8(7)^1^ ------------------------------------------------------------ 0

V9(1) ------------------------------------------------------------ 0

TP02_0244(genomic) GTTTCTACTCAGCATACCTGGTCGCAGATAAGGTGACAGTAGTGTCCAAGAACAACGCAG 780

XM_759717(mRNA) GTTTCTACTCAGCATACCTGGTCGCAGATAAGGTGACAGTAGTGTCCAAGAACAACGCAG 550

V1(2) ---------------ACCTGGTCGCAGATAAGGTGACAGTAGTGTCCAAGAACAACGCAG 45

V2(4) -----------------------GCAGATAAGGTGACAGTAGTGTCCAAGAACAACGCAG 37

V3(1) -----------------------GCAGATAAGGTGACAGTAGTGTCCAAGAACAACGCAG 37

V4(1) --------------------------------------------------------GCAG 4

V5(2) -----------------------GCAGATAAGGTGACAGTAGTGTCCAAGAACAACGCAG 37

V6(1) -----------------------GCAGATAAGGTGACAGTAGTGTCCAAGAACAACGCAG 37

V7(7) -----------------------GCAGATAAGGTGACAGTAGTGTCCAAGAACAACGCAG 37

V8(7) -----------------------GCAGATAAGGTGACAGTAGTGTCCAAGAACAACGCAG 37

V9(1) -----------------------GCAGATAAGGTGACAGTAGTTTCCAAGAACAATGCAG 37

****

TP02_0244(genomic) ACGACCAGTACGTCTGGGAGTCAACAGCCTCAGGCCACTTTACAGTGAAGAAGGACGACT 840

XM_759717(mRNA) ACGACCAGTACGTCTGGGAGTCAACAGCCTCAGGCCACTTTACAGTGAAGAAGGACGACT 610

V1(2) ACGACCAGTACGTCTGGGAGTCAACAGCCTCAGGCCACTTTACAGTGAAGAAGGACGACT 105

V2(4) ACGACCAGTACGTCTGGGAGTCAACAGCCTCAGGCCACTTTACAGTGAAGAAGGACGACT 97

V3(1) ACGACCAGTACGTCTGGGAGTCAACAGCCTCAGGCCACTTTACAGTGAAGAAGGACGACT 97

V4(1) ACGACCAGTACGTCTGGGAGTCAACAGCCTCAGGCCACTTTACAGTGAAGAAGGACGACT 64

V5(2) ACGACCAGTACGTCTGGGAGTCAACAGCCTCAGGCCACTTTACAGTGAAGAAGGACGACT 97

V6(1) ACGACCAGTACGTCTGGGAGTCAACAGCCTCAGGCCACTTTACAGTGAAGAAGGACGACT 97

V7(7) ACGACCAGTACGTCTGGGAGTCAACAGCCTCAGGCCACTTTACAGTGAAGAAGGACGACT 97

V8(7) ACGACCAGTACGTCTGGGAGTCAACAGCCTCAGGCCACTTTACAGTGAAGAAGGACGACT 97

V9(1) ATGACCAGTACGTCTGGGAGTCAACAGCCTCAGGTCACTTTACAGTGAAGAAGGACGACT 97

* ******************************** *************************

TP02_0244(genomic) CGCACGAGCCGCTCAAAAGAGGAACTAGACTAATACTGCACTTGAAGGAGGACCAAACTG 900

XM_759717(mRNA) CGCACGAGCCGCTCAAAAGAGGAACTAGACTAATACTGCACTTGAAGGAGGACCAAACTG 670

V1(2) CGCACGAGCCGCTCAAAAGAGGAACTAGACTAATACTGCACTTGAAGGAGGACCAAACTG 165

V2(4) CGCACGAGCCGCTCAAAAGAGGAACGAGACTAATACTGCACTTGAAGGAGGACCAAACTG 157

V3(1) CGCACGAGCCGCTCAAAAGAGGAACGAGACTAATACTGCACTTGAAGGAGGACCAAACTG 157

V4(1) CGCACGAGCCGCTCAAAAGAGGAACGAGACTAATACTGCACTTGAAGGAGGACCAAACTG 124

V5(2) CGCACGAGCCGCTCAAAAGAGGAACGAGACTAATACTGCACTTGAAGGAGGACCAAACTG 157

V6(1) CGCACGAGCCGCTCAAAAGAGGAACGAGACTAATACTGCACTTGAAGGAGGACCAAACTG 157

V7(7) CGCACGAGCCGCTCAAAAGAGGAACGAGACTAATACTGCACTTGAAGGAGGACCAAACTG 157

V8(7) CGCACGAGCCGCTCAAAAGAGGAACGAGACTAATACTGCACTTGAAGGAGGACCAAACTG 157

V9(1) CACACGAACCCCTCAAAAGAGGAACGAGACTAATAYTGCACTTGAAGGAGGAYCAGTCTG 157

* ***** ** ************** ********* **************** ** ***

TP02_0244(genomic) AGTACCTTGAGGAGAGAAGGCTGAAAGAGCTTGTTAAGAAGCACAGCGAGTTCATTTCAT 960

XM_759717(mRNA) AGTACCTTGAGGAGAGAAGGCTGAAAGAGCTTGTTAAGAAGCACAGCGAGTTCATTTCAT 730

V1(2) AGTACCTTGAGGAGAGAAGGCTGAAAGAGCTTGTTAAGAAGCACAGCGAGTTCATTTCAT 225

V2(4) AGTACCTTGAGGAGAGAAGACTGAAAGAGCTTGTTAAGAAGCACAGCGAGTTCATTTCAT 217

V3(1) AGTACCTTGAGGAGAGAAGGCTGAAAGAGCTTGTTAAGAAGCACAGCGAGTTCATTTCAT 217

V4(1) AGTACCTTGAGGAGAGAAGGCTGAAAGAGCTTGTTAAGAAGCACAGCGAGTTCATTTCAT 184

V5(2) AGTACCTTGAGGAGAGAAGGCTGAAAGAGCTTGTTAAGAAGCACAGCGAGTTCATTTCAT 217

V6(1) AGTACCTTGAGGAGAGAAGGCTGAAAGAGCTTGTTAAGAAGCACAGCGAGTTCATTTCAT 217

V7(7) AGTACCTTGAGGAGAGAAGGCTGAAAGAGCTTGTTAAGAAGCACAGCGAGTTCATTTCAT 217

V8(7) AGTACCTTGAGGAGAGAAGGCTGAAAGAGCTTGTTAAGAAGCACAGCGAGTTCATTTCAT 217

V9(1) AGTACCTTGAGGAGAGAAGGTTGAAGGAACTTGTYAAGAAACACAGCGAGTTCATTTCRT 217

******************* **** ** ***** ***** ***************** *

TP02_0244(genomic) TCCCAATCTCGCTCTCAGTAGAGAAGACCCAGGAGACCGAGGTCACTGACGACGAGGCAG 1020

XM_759717(mRNA) TCCCAATCTCGCTCTCAGTAGAGAAGACCCAGGAGACCGAGGTCACTGACGACGAGGCAG 790

V1(2) TCCCAATCTCGCTCTCAGTAGAGAAGACCCAGGAGACCGAGGTCACTGACGACGAGGCAG 285

V2(4) TCCCAATCTCGCTCTCGGTAGAGAAGACTCAGGAGACCGAGGTCACTGACGACGAGGCAG 277

V3(1) TCCCAATCTCGCTCTCGGTAGAGAAGACCCAGGAGACCGAGGTTACTGACGACGAGGCAG 277

V4(1) TCCCAATCTCGCTCTCAGTAGAGAAGACCCAGGAGACCGAGGTCACTGACGACGAGGCAG 244

V5(2) TCCCAATCTCACTCTCGGTAGAGAAGACTCAGGAGACCGAGGTCACTGACGACGAGGCAG 277

V6(1) TCCCAATCTCGCTCTCAGTAGAGAAGACTCAGGAGACCGAGGTCACTGACGACGAGGCAG 277

V7(7) TCCCAATCTCGCTCTCAGTAGAGAAGACCCAGGAGACCGAGGTCACTGACGACGAGGCAG 277

V8(7) TCCCAATCTCGCTCTCGGTAGAGAAGACTCAGGAGACCGAGGTCACTGACGACGAGGCAG 277

V9(1) TCCCAATCTCACTCTCAGTAGAGAAGACTCAAGAGACCGAAGTTACTGACGATGAGGCCG 277

********** ***** *********** ** ******** ** ******** ***** *

TP02_0244(genomic) AGCTAGACGAGGACAAGAAGCCCGAGGAGGAGAAGCCCAAGGACGATAAGGTGGAGGACG 1080

XM_759717(mRNA) AGCTAGACGAGGACAAGAAGCCCGAGGAGGAGAAGCCCAAGGACGATAAGGTGGAGGACG 850

V1(2) AGCTAGACGAGGACAAGAAGCCCGAGGAGGAGAAGCCCAAGGACGATAAGGTGGAGGACG 345

V2(4) AGCTAGACGAGGACAAGAAGCCCGAGGAGGAAAAGGCCAAGGACGATAAGGTGGA----- 332

V3(1) AGCTAGACGAGGACAAGAAGCCCGAGGAGGAAAAGGCCAAGGACGATAAGGTGGA----- 332

V4(1) AGCTAGACGAGGACAAGAAGCCCGAGGAGGAAAAGCCCAAGGACGATAAGGTGGA----- 299

V5(2) AGCTAGACGAGGACAAGAAGCCCGAGGAGGAAAAGGCCAAGGACGATAAGGTGGA----- 332

V6(1) AGCTAGACGAGGACAAGAAGCCCGAGGAGGAAAAGGCCAAGGACGATAAGGTGGA----- 332

V7(7) AGCTAGACGAGGACAAGAAGCCCGAGGAGGAAAAGGCCAAGGACGATAAGGTGGA----- 332

V8(7) AGCTAGACGAGGACAAGAAGCCCGAGGAGGAAAAGGCCAAGGACGATAAGGTGGA----- 332

V9(1) ACCTAGATGAGGATAAGAAACCTGAGGAAGAAAAGGACAAGGACCATAAGGTGGA----- 332

* ***** ***** ***** ** ***** ** *** ******* * ********

TP02_0244(genomic) TTACTGACGAGAAAGTGACCGAC***GTCACTGACGAGGAGGAGAAAAAG***GAGGAAAAGAAAA 1140

XM_759717(mRNA) TTACTGACGAGAAAGTGACCGACGTCACTGACGAGGAGGAGAAAAAGGAGGAAAAGAAAA 910

V1(2) TTACTGACGAGA------------------------------------------------ 357

V2(4) ------------------------------------------------------------ 332

V3(1) ------------------------------------------------------------ 332

V4(1) ------------------------------------------------------------ 299

V5(2) ------------------------------------------------------------ 332

V6(1) ------------------------------------------------------------ 332

V7(7) ------------------------------------------------------------ 332

V8(7) ------------------------------------------------------------ 332

V9(1) ------------------------------------------------------------ 332

Primer regions are in bold, underlined and italicized text.

^1^ Include X.1 variants.

**Table S9b: Tp7 protein variant Clustal alignment**

XP_764810 MSMIGQFGVGFYSAYLVADKVTVVSKNNADDQYVWESTASGHFTVKKDDSHEPLKRGTRL 180

V1(3) -----------------ADKVTVVSKNNADDQYVWESTASGHFTVKKDDSHEPLKRGTRL 43

V2(21) -----------------ADKVTVVSKNNADDQYVWESTASGHFTVKKDDSHEPLKRGTRL 43

V3(1) -----------------ADKVTVVSKNNADDQYVWESTASGHFTVKKDDSHEPLKRGTRL 43

*******************************************

XP_764810 ILHLKEDQTEYLEERRLKELVKKHSEFISFPISLSVEKTQETEVTDDEAELDEDKKPEEE 240

V1(3) ILHLKEDQTEYLEERRLKELVKKHSEFISFPISLSVEKTQETEVTDDEAELDEDKKPEEE 103

V2(21) ILHLKEDQTEYLEERRLKELVKKHSEFISFPISLSVEKTQETEVTDDEAELDEDKKPEEE 103

V3(1) ILHLKEDQSEYLEERRLKELVKKHSEFISFPISLSVEKTQETEVTDDEADLDEDKKPEEE 103

********:****************************************:**********

XP_764810 KPKDDKVEDVTDEKVTDVTDEEEKKEEKKKKKRKVTNVTREWEMLNKQKPIWMRLPSEVT 300

V1(3) KPKDDKV----------------------------------------------------- 110

V2(21) KAKDDKV----------------------------------------------------- 110

V3(1) KDKDHKV----------------------------------------------------- 110

* **.**

Eptiope region highlighted in grey.

**Table S10a: Tp8 nucleotide variant Clustal alignment**

TP02_0140(genomic) ***ATGCTTGGAAATCATGTCATGGGA***TCTAATTCCCCCCACATTAAAATTTTATCATCTGTT 60

XM_759616(mRNA) ATGCTTGGAAATCATGTCATGGGATCTAATTCCCCCCACATTAAAATTTTATCATCTGTT 60

V1(3) ------------------------------------------------------------ 0

V2(2) ------------------------------------------------------------ 0

V3(1) ------------------------------------------------------------ 0

V4(1) ------------------------------------------------------------ 0

V5(1) ------------------------------------------------------------ 0

V6(3) ------------------------------------------------------------ 0

V7(1) ------------------------------------------------------------ 0

V8(1) ------------------------------------------------------------ 0

V9(1) ------------------------------------------------------------ 0

V10(2)^1^ ------------------------------------------------------------ 0

V11(5)^1^ ------------------------------------------------------------ 0

V12(2) ------------------------------------------------------------ 0

TP02_0140(genomic) ACATTCTTACATATTGCTAAAATGGAAGAAGTAGAAAACGTAAAAGTCGACGCCTTGGAG 120

XM_759616(mRNA) ACATTCTTACATATTGCTAAAATGGAAGAAGTAGAAAACGTAAAAGTCGACGCCTTGGAG 120

V1(3) ---------------------------------------------GTCGACGCCTTGGAG 15

V2(2) ---------------------------------------------GTCGACGCCTTGGAG 15

V3(1) ---------------------------------------------GTCGACGCCTTGGAG 15

V4(1) ---------------------------------------------GTCGACGCCTTGGAG 15

V5(1) ---------------------------------------------GTCGACGCCTTGGAG 15

V6(3) ---------------------------------------------GTCGACGCCTTGGAG 15

V7(1) ---------------------------------------------GTCGACGCCTTGGAG 15

V8(1) ---------------------------------------------GTCGACGCCTTGGAG 15

V9(1) ---------------------------------------------GTCGACGCCTTGGAG 15

V10(2) ---------------------------------------------GTCGACGCCTTGGAG 15

V11(5) ---------------------------------------------GTCGACGCCTTGGAG 15

V12(2) ---------------------------------------------GTCGACGCCTTGGAG 15

***************

TP02_0140(genomic) CGTGTTGACACTGAGTCTGTCCTTAATTATGACACTGTGTTAGAAAAGAAACCATTGCGC 180

XM_759616(mRNA) CGTGTTGACACTGAGTCTGTCCTTAATTATGACACTGTGTTAGAAAAGAAACCATTGCGC 180

V1(3) CGTGTTGACACTGAGTCTGTCCTTAATTATGACACTGTGTTAGAAAAGAAACCATTGCGC 75

V2(2) CGTGTTGACACTGAGTCTGTCCTTAATTATGACACTGTGTTAGAAAAGAAACCATTGCGC 75

V3(1) CGTGTTGACACTGAGTCTGTCCTTAATTATGACACTGTGTTAGAAAAGAAACCATTGCGC 75

V4(1) CGTGTTGACACTGAGTCTGTCCTTAATTATGACACTGTGTTAGAAAAGAAACCATTGCGC 75

V5(1) CGTGTTGACACTGAGTCTGTCCTTAATTATGACACTGTGTTAGAAAAGAAACCATTGCGC 75

V6(3) CGTGTTGACACTGAGTCTGTCCTTAATTATGACACTGTGTTAGAAAAGAAACCATTGCGC 75

V7(1) CGTGTTGACACTGAGTCTGTCCTTAATTATGACACTGTGTTAGAAAAGAAACCATTGCGC 75

V8(1) CGTGTTGACACTGAGTCTGTCCTTAATTATGACACTGTGTTAGAAAAGAAACCATTGCGC 75

V9(1) CGTGTTGACACTGAGTCTGTCCTTAATTATGACACTGTGTTAGAAAAGAAACCATTGCGC 75

V10(2) CGTGTTGACACTGAGTCTGTCCTTAATTATGACACTGTGTTAGAAAAGAAACCATTGCGC 75

V11(5) CGTGTTGACACTGAGTCTGTCCTTAATTATGACACTGTGTTAGAAAAGAAACCATTGCGC 75

V12(2) CGTGTTGACACTGAGTCTGTCCTTAATTATGACACTGTGTTAGAAAAGAAACCATTGCGC 75

************************************************************

TP02_0140(genomic) AGCAGTGTTGCCTCTTTCTTCAAAAGATACAGTGCTGTTCTCGTAATATTAACTGCCGTG 240

XM_759616(mRNA) AGCAGTGTTGCCTCTTTCTTCAAAAGATACAGTGCTGTTCTCGTAATATTAACTGCCGTG 240

V1(3) AGCAGTGTTGCCTCTTTCTTCAAAAGATACAGTGCTGTTCTCGTAATATTAACTGCCGTG 135

V2(2) AGCAGTGTTGCCTCTTTCTTCAAAAGATACAGTGCTGTTCTCGTAATATTAACTGCCGTG 135

V3(1) AGCAGTGTTGCCTCTTTCTTCAAAAGATACAGTGCTGTTCTCGTAATATTAACTGCCGTG 135

V4(1) AGCAGTGTTGCCTCTTTCTTCAAAAGATACAGTGCTGTTCTCGTAATATTAACTGCCGTG 135

V5(1) AGCAGTGTTGCCTCTTTCTTCAAAAGATACAGTGCTGTTCTCGTAATATTAACTGCCGTG 135

V6(3) AGCAGTGTTGCCTCTTTCTTCAAAAGATACAGTGCTGTTCTCGTAATATTAACTGCCGTG 135

V7(1) AGCAGTGTTGCCTCTTTCTTCAAAAGATACAGTGCTGTTCTCGTAATATTAACTGCCGTG 135

V8(1) AGCAGTGTTGCCTCTTTCTTCAAAAGATACAGTGCTGTTCTCGTAATATTAACTGCCGTG 135

V9(1) AGCAGTGTTGCCTCTTTCTTCAAAAGATACAGTGCTGTTCTCGTAATATTAACTGCCGTG 135

V10(2) AGCAGTGTTGCCTCTTTCTTCAAAAGATACAGTGCTGTTCTCGTAATATTAACTGCCGTG 135

V11(5) AGCAGTGTTGCCTCTTTCTTCAAAAGATACAGTGCTGTTCTCGTAATATTAACTGCCGTG 135

V12(2) AGCAGTGTTGCCTCTTTCTTCAAAAGATACAGTGCTGTTCTCGTAATATTAACTGCCGTG 135

************************************************************

TP02_0140(genomic) CTATTATTCACATTCACTTTTGCAGCAATAGCATTGTCATCAGGCAGAAGCGCAATCAGA 300

XM_759616(mRNA) CTATTATTCACATTCACTTTTGCAGCAATAGCATTGTCATCAGGCAGAAGCGCAATCAGA 300

V1(3) CTATTATTCACATTCACTTTTGCAGCAATAGCATTGTCATCAGGCAGAAGCGCAATCAGA 195

V2(2) CTATTATTCACATTCACTTTTGCAGCAATAGCATTGTCATCAGGCAGAAGCGCAATCAGA 195

V3(1) CTATTATTCACATTCACTTTTGCAGCAATAGCATTGTCATCAGGCAGAAGCGCAATCAGA 195

V4(1) CTATTATTCACATTCACTTTTGCAGCAATAGCATTGTCATCAGGCAGAAGCGCAATCAGA 195

V5(1) CTATTATTCACATTCACTTTTGCAGCAATAGCATTGTCATCAGGCAGAAGCGCAATCAGA 195

V6(3) CTATTATTCACATTCACTTTTGCAGCAATAGCATTGTCATCAGGCAGAAGCGCAATCAGA 195

V7(1) CTATTATTCACATTCACTTTTGCAGCAATAGCATTGTCATCAGGCAGAAGCGCAATCAGA 195

V8(1) CTATTATTCACATTCACTTTTGCAGCAATAGCATTGTCATCAGGCAGAAGCGCAATCAGA 195

V9(1) CTATTATTCACATTCACTTTTGCAGCAATAGCATTGTCATCAGGCAGAAGCGCAATCAGA 195

V10(2) CTATTATTCACATTCACTTTTGCAGCAATAGCATTGTCATCAGGCAGAAGCGCAATCAGA 195

V11(5) CTATTATTCACATTCACTTTTGCAGCAATAGCATTGTCATCAGGCAGAAGCGCAATCAGA 195

V12(2) CTATTATTCACATTCACTTTTGCAGCAATAGCATTGTCATCAGGCAGAAGCGCAATCAGA 195

************************************************************

TP02_0140(genomic) AAGAACAGAGAACTCCTGTCAGTCGAATTTGAAAAGCTTCAGTTCGATAATTTCGTGACA 360

XM_759616(mRNA) AAGAACAGAGAACTCCTGTCAGTCGAATTTGAAAAGCTTCAGTTCGATAATTTCGTGACA 360

V1(3) AAGAACAGAGAACTCCTGTCAGTCGAATTTGAAAAGCTTCAGTTCGATAATTTCGTGACA 255

V2(2) AAGAACAGAGAACTCCTGTCAGTCGAATTTGAAAAGCTTCAGTTCGATAATTACGTGACA 255

V3(1) AAGAACAGAGAACTCCTGTCAGTCGAATTTGAAAAGCTTCAGTTCGATAATTTCGTGACA 255

V4(1) AAGAACAGAGAACTCCTGTCAGTCGAATTTGAAAAGCTTCAGTTCGATAATTTCGTGACA 255

V5(1) AAGAACAGAGAACTCCTGTCAGTCGAATTTGAAAAGCTTCAGTTCGATAATTTCGTGACA 255

V6(3) AAGAACAGAGAACTCCTGTCAGTCGAATTTGAAAAGCTTCAGTTCGATAATTTCGTGACA 255

V7(1) AAGAACAGAGAACTCCTGTCAGTCGAATTTGAAAAGCTTCAGTTCGATAATTTCGTGACA 255

V8(1) AAGAACAGAGAACTCCTGTCAGTCGAATTTGAAAAGCTTCAGTTCGATAATTTCGTGACA 255

V9(1) AAGAACAGAGAACTCCTGTCAGTCGAATTTGAAAAGCTTCAGTTCGATAATTTCGTGACA 255

V10(2) AAGAACAGAGAACTCCTGTCAGTCGAATTTGAAAAGCTTCAGTTCGATAATTTCGTGACA 255

V11(5) AAGAACAGAGAACTCCTGTCAGTCGAATTTGAAAAGCTTCAGTTCGATAATTTCGTGACA 255

V12(2) AAGAACAGAGAACTCCTGTCAGTCGAATTTGAAAAGCTTCAGTTCGATAATTTCGTGACA 255

**************************************************** *******

TP02_0140(genomic) ATTAAGGGAGAAAGGGAAGAGGACTTCCCCAAGATGGTAGCTGAAGTTCTTTACAAGGTT 420

XM_759616(mRNA) ATTAAGGGAGAAAGGGAAGAGGACTTCCCCAAGATGGTAGCTGAAGTTCTTTACAAGGTT 420

V1(3) ATTAAGGGAGAAAGGGAAGAGGACTTCCCCAAGATGGTAGCTGAAGTTCTTTACAAGGTT 315

V2(2) ATTAAGGGAGAAAGGGAAGAGGACTTCCCCAAGATGGTAGCTGAAGTTCTTTACAAAGTT 315

V3(1) ATTAAGGGAGAAAGGGAAGAGGACTTCCCCAAGATGGTAGCTGAAGTTCTTTACAAAGTT 315

V4(1) ATTAAGGGAGAAAGGGAAGAGGACTTCCCCAAGATGGTAGCTGAAGTTCTTTACAAAGTT 315

V5(1) ATTAAGGGAGAAAGGGAAGAGGACTTCCCCAAGATGGTAGCTGAAGTTCTTTACAAAGTT 315

V6(3) ATTAAGGGAGAAAGGGAAGAGGACTTCCCCAAGATGGTAGCTGAAGTTCTTTACAAAGTT 315

V7(1) ATTAAGGGAGAAAGGGAAGAGGACTTCCCCAAGATGGTAGCTGAAGTTCTTTACAAAGTT 315

V8(1) ATTAAGGGAGAAAGGGAAGAGGACTTCCCCAAGATGGTAGCTGAAGTTCTTTACAAAGTT 315

V9(1) ATTAAGGGAGAAAGGGAAGAGGACTTCCCCAAGATGGTAGCTGAAGTTCTTTACAAAGTT 315

V10(2) ATTAAGGGAGAAAGGGAAGAGGACTTCCCCAAGATGGTAGCTGAAGTTCTTTACAAAGTT 315

V11(5) ATTAAGGGAGAAAGGGAAGAGGACTTCCCCAAGATGGTAGCTGAAGTTCTTTACAAAGTT 315

V12(2) ATTAAGGGAGAAAGGGAAGAGGACTTCCCCAAGATGGTAGCTGAAGTTCTTTACAAGGTT 315

******************************************************** ***

TP02_0140(genomic) GCAGTCGAGTTTGACCCAAAAGAAGAGGCCTTGATCTACGTCCAGTTCAATGACTTCAAC 480

XM_759616(mRNA) GCAGTCGAGTTTGACCCAAAAGAAGAGGCCTTGATCTACGTCCAGTTCAATGACTTCAAC 480

V1(3) GCAGTCGAGTTTGACCCAAAAGAAGAGGCCTTGATCTACGTCCAGTTCAATGACTTCAAC 375

V2(2) GCAGTCGAGTTTGACCCAAAAGAAGAGGCTTTGATCTACGTCCAGTTCAATGACTTTAAC 375

V3(1) GCAGTCGAGTTTGACCCAAAAGAAGAGGCTTTGATCTACGTCCAGTTCAATGACTTTAAC 375

V4(1) GCAGTCGAGTTTGACCCAAAAGAAGAGGCCTTGATCTACGTCCAGTTCAATGACTTCAAC 375

V5(1) GCAGTCGAGTTTGACCCAAAAGAAGAGGCCTTGATCTACGTCCAGTTCAATGACTTCAAC 375

V6(3) GCAGTCGAGTTTGACCCAAAAGAAGAGGCCTTGATCTACGTCCAGTTCAATGACTTCAAC 375

V7(1) GCAGTCGAGTTTGACCCAAAAGAAGAGGCCTTGATCTACGTCCAGTTCAATGACTTCAAC 375

V8(1) GCAGTCGAGTTTGACCCAAAAGAAGAGGCCTTGATCTACGTCCAGTTCAATGACTTCAAC 375

V9(1) GCAGTCGAGTTTGACCCAAAAGAAGAGGCCTTGATCTACGTCCAGTTCAATGACTTCAAC 375

V10(2) GCAGTCGAGTTTGACCCAAAAGAAGAGGCCTTGATCTACGTCCAGTTCAATGACTTCAAC 375

V11(5) GCAGTCGAGTTTGACCCAAAAGAAGAGGCCTTGATCTACGTCCAGTTCAATGACTTCAAC 375

V12(2) GCAGTCGAGTTTGACCCAAAAGAAGAGGCCTTGATCTACGTCCAGTTCAATGACTTCAAC 375

***************************** ************************** ***

TP02_0140(genomic) AAGCAACACGACAAGAAGCACAACAATTACAGGCACAAGAAGACCTCGTACACCAACTTC 540

XM_759616(mRNA) AAGCAACACGACAAGAAGCACAACAATTACAGGCACAAGAAGACCTCGTACACCAACTTC 540

V1(3) AAGCAACACGACAAGAAGCACAACAATTACAGGCACAAGAAGACCTCGTACACCAACTTC 435

V2(2) AAGCAACACGACAAGAAGCACAACAATTACAGGCACAAGAAGACCTCGTACACCAACTTC 435

V3(1) AAGCAACACGACAAGAAGCACAACAATTACAGGCACAAGAAGACCTCGTACACCAACTTC 435

V4(1) AAGCAACACGACAAGAAGCACAACAATTACAGGCACAAGAAGACCTCGTACACCAACTTC 435

V5(1) AAGCAACACGACAAGAAGCACAACAATTACAGGCACAAGAAGACCTCGTACACCAACTTC 435

V6(3) AAGCAACACGACAAGAAGCACAACAATTACAGGCACAAGAAGACCTCGTACACCAACTTC 435

V7(1) AAGCAACACGACAAGAAGCACAACAATTACAGGCACAAGAAGACCTCGTACACCAACTTC 435

V8(1) AAGCAACACGACAAGAAGCACAACAATTACAGGCACAAGAAGACCTCGTACACCAACTTC 435

V9(1) AAGCAACACGACAAGAAGCACAACAATTACAGGCACAAGAAGACCTCGTACACCAACTTC 435

V10(2) AAGCAACACGACAAGAAGCACAACAATTACAGGCACAAGAAGACCTCGTACACCAACTTC 435

V11(5) AAGCAACACGACAAGAAGCACAACAATTACAGGCACAAGAAGACCTCGTACACCAACTTC 435

V12(2) AAGCAACACGACAAGAAGCACAACAATTACAGGCACAAGAAGACCTCGTACACCAACTTC 435

************************************************************

TP02_0140(genomic) AGAAACAACCTTAATGATATAAACGAGCACAACGCAAAACCAAACCTGTCGTACACCAAG 600

XM_759616(mRNA) AGAAACAACCTTAATGATATAAACGAGCACAACGCAAAACCAAACCTGTCGTACACCAAG 600

V1(3) AGAAACAACCTTAATGATATAAACGAGCACAACGCAAAACCAAACCTGTCGTACACCAAG 495

V2(2) AGAAACAACCTTAATGATATAAACGAGCACAACGCAAAACCAAACCTGTCGTACACCAAG 495

V3(1) AGAGACAACCTTAATGATATAAACGAGCACAACGCAAAACCAAACCTGTCCTACACCAAG 495

V4(1) AGAAACAACCTTAATGATATAAACGAGCACAACGCAAAACCAAACCTGTCGTACACCAAG 495

V5(1) AGAAACAACCTTAATGATATAAACGAGCACAACGCAAAACCAAACCTGTCGTACACCAAG 495

V6(3) AGAAACAACCTTAATGATATAAACGAGCACAACGCAAAACCAAACCTGTCGTACACCAAG 495

V7(1) AGAAACAACCTTAATGATATAAACGAGCACAACGCAAAACCAAACCTGTCGTACACCAAG 495

V8(1) AGAAACAACCTTAATGATATAAACGAGCACAACGCAAAACCAAACCTGTCGTACACCAAG 495

V9(1) AGAAACAACCTTAATGATATAAACGAGCACAACGCAAAACCAAACCTGTCGTACACCAAG 495

V10(2) AGAAACAACCTTAATGATATAAACGAGCACAACGCAAAACCAAACCTGTCGTACACCAAG 495

V11(5) AGAAACAACCTTAATGATATAAACGAGCACAACGCAAAACCAAACCTGTCGTACACCAAG 495

V12(2) AGAAACAACCTTAATGATATAAACGAGCACAACGCAAAACCAAACCTGTCGTACACCAAG 495

*** *******************************************************

TP02_0140(genomic) AACATGAACCACTTCGGTGACATATCATCCAAGGATTTCATGAAGAGATACACCAAGAAA 660

XM_759616(mRNA) AACATGAACCACTTCGGTGACATATCATCCAAGGATTTCATGAAGAGATACACCAAGAAA 660

V1(3) AACATGAACCACTTCGGTGACATATCATCCAAGGATTTCATGAAGAGATACACCAAGAAA 555

V2(2) AGCATGAACCACTTCGGTGACATATCATCCAAGGATTTCATGAAGAGATACACCAAGAAA 555

V3(1) AGCATGAACCACTTCGGTGACATATCATCCAAGGATTTCATGAAGAGATACACCAAGAAA 555

V4(1) AGCATGAACCACTTCGGTGACATATCATCCAAGGATTTCATGAAGAGATACACCAAGAAA 555

V5(1) AGCATGAACCACTTCGGTGACATATCATCCAAGGATTTCATGAAGAGATACACCAAGAAA 555

V6(3) AGCATGAACCACTTCGGTGACATATCATCCAAGGATTTCATGAAGAGATACACCAAGAAA 555

V7(1) AGCATGAACCACTTCGGTGACATATCATCCAAGGATTTCATGAAGAGATACACCAAGAAA 555

V8(1) AGCATGAACCACTTCGGTGACATATCATCCAAGGATTTCATGAAGAGATACACCAAGAAA 555

V9(1) AGCATGAACCACTTCGGTGACATATCATCCAAGGATTTCATGAAGAGATACACCAAGAAA 555

V10(2) AGCATGAACCACTTCGGTGACATATCATCCAAGGATTTCATGAAGAGATACACCAAGAAA 555

V11(5) AGCATGAACCACTTCGGTGACATATCATCCAAGGATTTCATGAAGAGATACACCAAGAAA 555

V12(2) AGCATGAACCACTTCGGTGACATATCATCCAAGGATTTCATGAAGAGATACACCAAGAAA 555

* **********************************************************

TP02_0140(genomic) GTACTCTTGAACTTGCCAAAAGACCACGTGTCCACCTATAACAACAACAGACCAATGTCA 720

XM_759616(mRNA) GTACTCTTGAACTTGCCAAAAGACCACGTGTCCACCTATAACAACAACAGACCAATGTCA 720

V1(3) GTACTCTTGAACTTGCCAAAAGACCACGTGTCCACCTATAACAACAACAGACCAATGTCA 615

V2(2) GTACTCTTGAACTTGCCAAAAGACCACGCGTCCACCTATAACAACAACAGACCAATGTCA 615

V3(1) GTACTCTTGAACTTGCCAAAAGACCACGTGTCCACCTATAACAACAACAGACCAATGTCA 615

V4(1) GTACTCTTGAACTTGCCAAAAGACCACGCGTCCACCTATAACAACAACAGACCAATGTCA 615

V5(1) GTACTCTTGAACTTGCCAAAAGACCACGTGTCCACCTATAATAACAACAGACCAATGTCA 615

V6(3) GTACTCTTGAACTTGCCAAAAGACCACGTGTCCACCTATAATAACAACAGACCAATGTCA 615

V7(1) GTACTCTTGAACTTGCCAAAAGACCACGTGTCCACCTATAACAACAACAGACCAATGTCA 615

V8(1) GTACTCTTGAACTTGCCAAAAGACCACGTGTCCACCTATAACAACAACAGACCAATGTCA 615

V9(1) GTACTCTTGAACTTGCCAAAAGACCACGTGTCCACCTATAACAACAACAGACCAATGTCA 615

V10(2) GTACTCTTGAACTTGCCAAAAGACCACGTGTCCACCTATAACAACAACAGACCAATGTCA 615

V11(5) GTACTCTTGAACTTGCCAAAAGACCACGTGTCCACCTATAACAACAACAGACCAATGTCA 615

V12(2) GTACTCTTGAACTTGCCAAAAGACCACGTGTCCACCTATAACAACAACAGACCAATGTCA 615

**************************** ************ ******************

TP02_0140(genomic) GTTGATCTCAGAAGCCATGGTGTATTGACTCCAGTCAAGTGCCAAGAAGAAAATGAACTC 780

XM_759616(mRNA) GTTGATCTCAGAAGCCATGGTGTATTGACTCCAGTCAAGTGCCAAGAAGAAAATGAACTC 780

V1(3) GTTGATCTCAGAAGCCATGGTGTATTGACTCCAGTCAAGTGCCAAGAAGAAAATGAACTC 675

V2(2) GTTGATCTCAGAAGCCATGGTGTATTGACTCCAGTCAAGTGCCAAGAAGAAAATGAACTC 675

V3(1) GTTGATCTCAGAAGCCATGGTGTATTGACTCCAGTCAAGTGCCAAGAAGAAAATGAACTC 675

V4(1) GTTGATCTCAGAAGCCATGGTGTATTGACTCCAGTCAAGTGCCAAGAAGAAAATGAACTC 675

V5(1) GTTGATCTCAGAAGCCATGGTGTATTGACTCCAGTCAAGTGCCAAGAAGAAAATGAACTC 675

V6(3) GTTGATCTCAGAAGCCATGGTGTATTAACTCCAGTCAAGTGCCAAGAAGAAAATGAACTC 675

V7(1) GTTGATCTCAGAAGCCATGGTGTATTAACTCCAGTCAAGTGCCAAGAAGAAAATGAACTC 675

V8(1) GTTGATCTCAGAAGCCATGGTGTATTAACTCCAGTCAAGTGCCAAGAAGAAAATGAACTC 675

V9(1) GTTGATCTCAGAAGCCATGGTGTATTAACTCCAGTCAAGTGCCAAGAAGAAAATGAACTC 675

V10(2) GTTGATCTCAGAAGCCATGGTGTATTGACTCCAGTCAAGTGCCAAGAAGAAAATGAACTC 675

V11(5) GTTGATCTCAGAAGCCATGGTGTATTGACTCCAGTCAAGTGCCAAGAAGAAAATGAACTC 675

V12(2) GTTGATCTCAGAAGCCATGGTGTATTGACTCCAGTCAAGTGCCAAGAAGAAAATGAACTC 675

************************** *********************************

TP02_0140(genomic) TCATGGCCATACTCCGTAGTAGCAGTCGCCGAGTCATTCGTTAAGAAGACATCACAAAAG 840

XM_759616(mRNA) TCATGGCCATACTCCGTAGTAGCAGTCGCCGAGTCATTCGTTAAGAAGACATCACAAAAG 840

V1(3) TCATGGCCATACTCCGTAGTAGCAGTCGCCGAGTCATTCGTTAAGAAGACATCACAAAAG 735

V2(2) TCATGGCCGTACTCCGTAGTAGCAGTCGCCGAGTCATTCGTTAAGAAGACATCACAAAAG 735

V3(1) TCATGGCCATACTCCGTAGTAGCAGTCGCCGAATCATTCGTTAAGAAGACATCACAAAAG 735

V4(1) TCATGGCCATACTCCGTAGTAGCAGTCGCCGAGTCATTCGTTAAGAAGACATCACAAAAG 735

V5(1) TCATGGCCGTACTCCGTAGTAGCAGTCGCCGAGTCATTCGTTAAGAAGACATCACAAAAG 735

V6(3) TCATGGCCATACTCCGTAGTAGCAGTCGCCGAGTCATTCGTTAAGAAGACATCACAAAAG 735

V7(1) TCATGGCCATACTCCGTAGTAGCAGTCGCCGAGTCATTCGTTAAGAAGACATCACAAAAG 735

V8(1) TCATGGCCATACTCCGTAGTAGCAGTCGCCGAGTCATTCGTTAAGAAGACATCACAAAAG 735

V9(1) TCATGGCCATACTCCGTAGTAGCAGTCGCCGAGTCATTCGTTAAGAAGACATCACAAAAG 735

V10(2) TCATGGCCATACTCCGTAGTAGCAGTCGCCGAGTCATTCGTTAAGAAGACATCACAAAAG 735

V11(5) TCATGGCCGTACTCCGTAGTAGCAGTCGCCGAGTCATTCGTTAAGAAGACATCACAAAAG 735

V12(2) TCATGGCCGTACTCCGTAGTAGCAGTCGCCGAGTCATTCGTTAAGAAGACATCACAAAAG 735

******** *********************** ***************************

TP02_0140(genomic) ACCGTATCCCTCAGCGAAAAACAATTAGTAGATTGCGTTACAGATAAGAAATCTGCAAAC 900

XM_759616(mRNA) ACCGTATCCCTCAGCGAAAAACAATTAGTAGATTGCGTTACAGATAAGAAATCTGCAAAC 900

V1(3) ACCGTATCCCTCAGCGAAAAACAATTAGTAGATTGCGTTACAGATAAGAAATCTGCAAAC 795

V2(2) ACCGTATCCCTCAGCGAAAAACAATTAGTAGATTGCGTTACAGATAAGAAATCTGCAAAC 795

V3(1) ACCGTATCCCTCAGCGAAAAACAATTAGTAGATTGCGTTACAGATAAGAAATCAGCAAAC 795

V4(1) ACCGTATCCCTCAGCGAAAAACAATTAGTAGATTGCGTCACAGATAAGAAATCTGCAAAC 795

V5(1) ACCGTATCCCTCAGCGAAAAACAATTAGTAGATTGCGTTACAGATAAGAAATCTGCAAAC 795

V6(3) ACCGTATCCCTCAGCGAAAAACAATTAGTAGATTGCGTTACAGATAAGAAATCTGCAAAC 795

V7(1) ACCGTATCCCTCAGCGAAAAACAATTAGTAGATTGCGTTACAGATAAGAAATCTGTAAAC 795

V8(1) ACCGTATCCCTCAGCGAAAAACAATTAGTAGATTGCGTTACAGATAAGAAATCTGCAAAC 795

V9(1) ACCGTATCCCTCAGCGAAAAACAATTAGTAGATTGCGTTACAGATAAGAAATCTGCAAAC 795

V10(2) ACCGTATCCCTCAGCGAAAAACAATTAGTAGATTGCGTTACAGATAAGAAATCTGCAAAC 795

V11(5) ACCGTATCCCTCAGCGAAAAACAATTAGTAGATTGCGTTACAGATAAGAAATCTGCAAAC 795

V12(2) ACCGTATCCCTCAGCGAAAAACAATTAGTAGATTGCGTTACAGATAAGAAATCTGCAAAC 795

************************************** ************** * ****

TP02_0140(genomic) AACCCATTCTTGGGTTACAAATACCTTAAGGACTTGGGTCTGTTCGAATCAGAACTCGTA 960

XM_759616(mRNA) AACCCATTCTTGGGTTACAAATACCTTAAGGACTTGGGTCTGTTCGAATCAGAACTCGTA 960

V1(3) AACCCATTCTTGGGTTACAAATACCTTAAGGACTTGGGTCTGTTCGAATCAGAACTCGTA 855

V2(2) AACCCATTCTTGGGTTACAAATACCTTAAGGACTTGGGTCTGTTCGAATCAGAACTCGTA 855

V3(1) AACCCATTCTTGGGTTACAAATACCTTAAGGACTTGGGTCTGTTCGAATCAGAACTCGTA 855

V4(1) AACCCATTCTTGGGTTACAAATACCTTAAGGACTTGGGTCTGTTCGAATCAGAACTCGTA 855

V5(1) AACCCATTCTTGGGTTACAAATACCTTAAGGACTTGGGTCTGTTCGAATCAGAACTCGTA 855

V6(3) AACCCATTCTTGGGTTACAAATACCTTAAGGACTTGGGTCTGTTCGAATCAGAACTCGTA 855

V7(1) AACCCATTCTTGGGTTACAAATACCTTAAGGACTTGGGTCTGTTCGAATCAGAACTCGTA 855

V8(1) AACCCATTCTTGGGTTACAAATACCTTAAGGACTTGGGTCTGTTCGAATCAGAACTCGTA 855

V9(1) AACCCATTCTTGGGTTACAAATACCTTAAGGACTTGGGTCTGTTCGAATCAGAACTCGTA 855

V10(2) AACCCATTCTTGGGTTACAAATACCTTAAGGACTTGGGTCTGTTCGAATCAGAACTCGTA 855

V11(5) AACCCATTCTTGGGTTACAAATACCTTAAGGACTTGGGTCTGTTCGAATCAGAACTCGTA 855

V12(2) AACCCATTCTTGGGTTACAAATACCTTAAGGACTTGGGTCTGTTCGAATCAGAACTCGTA 855

************************************************************

TP02_0140(genomic) GACAAATCCACAACCAAGTGCCCAGCATTGGAAGGTGAAAGATTCAAAGTCCCATCATAC 1020

XM_759616(mRNA) GACAAATCCACAACCAAGTGCCCAGCATTGGAAGGTGAAAGATTCAAAGTCCCATCATAC 1020

V1(3) GACAAATCCACAACCAAGTGCCCAGCATTGGAAGGTGAAAGATTCAAAGTCCCATCATAC 915

V2(2) GACAAATCCACAACCAAGTGCCCAGCATTGGAAGGTGAAAGATTCAAAGTCCCATCATAC 915

V3(1) GACAAATCCACAACCAAGTGCCCAGCATTGGAAGGTGAAAGATTCAAAGTCCCATCATAC 915

V4(1) GACAAATCCACAACCAAGTGCCCAGCATTGGAAGGTGAAAGATTCAAAGTCCCATCATAC 915

V5(1) GACAAATCCACAACCAAGTGCCCAGCATTGGAAGGTGAAAGATTCAAAGTCCCATCATAC 915

V6(3) GACAAATCCACAACCAAGTGCCCAGCATTGGAAGGTGAAAGATTCAAAGTCCCATCATAC 915

V7(1) GACAAATCCGCAACCAAGTGCCCAGCATTGGAAGGTGAAAGATTCAAAGTCCCGTCATAC 915

V8(1) GACAAATCCGCAACCAAGTGCCCAGCATTGGAAGGTGAAAGATTCAAAGTCCCGTCATAC 915

V9(1) GACAAATCCGCAACCAAGTGCCCAGCATTGGAAGGTGAAAGATTCAAAGTCCCATCATAC 915

V10(2) GACAAATCCGCAACCAAGTGCCCAGCATTGGAAGGTGAAAGATTCAAAGTCCCATCATAC 915

V11(5) GACAAATCCGCAACCAAGTGCCCAGCATTGGAAGGTGAAAGATTCAAAGTCCCATCATAC 915

V12(2) GACAAATCCGCAACCAAGTGCCCAGCATTGGAAGGTGAAAGATTCAAAGTCCCATCATAC 915

********* ******************************************* ******

TP02_0140(genomic) TCATACTCATATGAGCCAGATTTGGTGGCACTCTTGTTGAATGCAGGACCACTCACTGTA 1080

XM_759616(mRNA) TCATACTCATATGAGCCAGATTTGGTGGCACTCTTGTTGAATGCAGGACCACTCACTGTA 1080

V1(3) TCATACTCATATGAGCCAGATTTGGTGGCACTCTTGTTGAATGCAGGACCACTCACTGTA 975

V2(2) TCATACTCATATGAGCCAGATTTGGTGGCACTCTTGTTGAATGCAGGACCACTCACTGTA 975

V3(1) TCATACTCATATGAGCCCGATTTGGTGGCACTCTTGTTGAATGCAGGACCACTCACTGTA 975

V4(1) TCATACTCATATGAGCCAGATTTGGTGGCACTCTTGTTGAATGCAGGACCACTCACTGTA 975

V5(1) TCATACTCATATGAGCCCGATTTGGTGGCACTCTTGTTGAATGCAGGACCACTCACTGTA 975

V6(3) TCATACTCATATGAGCCCGATTTGGTGGCACTCTTGTTGAATGCAGGACCACTCACTGTA 975

V7(1) TCATACTCATATGAGCCAGATTTGGTGGCACTCTTGTTGAATGCAGGACCACTCACTGTA 975

V8(1) TCATACTCATATGAGCCAGATTTGGTGGCACTCTTGTTGAATGCAGGACCACTCACTGTA 975

V9(1) TCATACTCATATGAGCCAGATTTGGTGGCACTCTTGTTGAATGCAGGACCACTCACTGTA 975

V10(2) TCATACTCATATGAGCCAGATTTGGTGGCACTCTTGTTGAATGCAGGACCACTCACTGTA 975

V11(5) TCATACTCATATGAGCCAGATTTGGTGGCACTCTTGTTGAATGCAGGACCACTCACTGTA 975

V12(2) TCATACTCATATGAGCCAGATTTGGTGGCACTCTTGTTGAATGCAGGACCACTCACTGTA 975

***************** ******************************************

TP02_0140(genomic) CCAGTTGCAGTGAGCGAGGATTGGCAATTCTACGCTGATGGAACCTTGGATGTATGCGGT 1140

XM_759616(mRNA) CCAGTTGCAGTGAGCGAGGATTGGCAATTCTACGCTGATGGAACCTTGGATGTATGCGGT 1140

V1(3) CCAGTTGCAGTGAGCGAGGATTGGCAATTCTACGCTGATGGAACCTTGGATGTATGCGGT 1035

V2(2) CCAGTTGCAGTGAGCGAGGATTGGCAATTCTACGCTGATGGAACCTTGGATGTGTGCGGT 1035

V3(1) CCAGTTGCAGTGAGCGAGGATTGGCAATTCTACGCTGATGGAACCTTGGATGTGTGCGGT 1035

V4(1) CCAGTTGCAGTGAGCGAGGATTGGCAATTCTACGCTGATGGAACCTTGGATGTGTGCGGT 1035

V5(1) CCAGTTGCAGTGAGCGAGGATTGGCAATTCTACGCTGATGGAACCTTGGATGTGTGCGGT 1035

V6(3) CCAGTTGCAGTGAGCGAGGATTGGCAATTCTACGCTGATGGAACCTTGGATGTGTGCGGT 1035

V7(1) CCAGTTGCAGTGAGCGAGGATTGGCAATTCTACGCTGATGGAACCTTGGATGTGTGCGGT 1035

V8(1) CCAGTTGCAGTGAGCGAGGATTGGCAATTCTACGCTGATGGAACCTTGGATGTGTGCGGT 1035

V9(1) CCAGTTGCAGTGAGCGAGGATTGGCAATTCTACGCTGATGGAACCTTGGATGTGTGCGGT 1035

V10(2) CCAGTTGCAGTGAGCGAGGATTGGCAATTCTACGCTGATGGAACCTTGGATGTGTGCGGT 1035

V11(5) CCAGTTGCAGTGAGCGAGGATTGGCAATTCTACGCTGATGGAACCTTGGATGTGTGCGGT 1035

V12(2) CCAGTTGCAGTGAGCGAGGATTGGCAATTCTACGCTGATGGAACCTTGGATGTGTGCGGT 1035

***************************************************** ******

TP02_0140(genomic) GCTGAATTGAACCACTTCTTGACCCTAGTAGGTGTCAGCTTTGACGAAAAAGGCAATCAC 1200

XM_759616(mRNA) GCTGAATTGAACCACTTCTTGACCCTAGTAGGTGTCAGCTTTGACGAAAAAGGCAATCAC 1200

V1(3) GCTGAATTGAACCACTTCTTGACCCTAGTAGGTGTCAGCTTTGACGAAAAAGGCAATCAC 1095

V2(2) GCTGAATTGAACCACTTCTTGACCCTAGTAGGTGTCAGCTTTGACGAAAAAGGCAATCAC 1095

V3(1) GCTGAATTGAACCACTTCTTGACCCTAGTAGGTGTCAGCTTTGACGAAAAAGGCAATCAC 1095

V4(1) GCTGAATTGAACCACTTCTTGACCCTAGTAGGTGTCAGCTTTGACGAAAAAGGCAATCAC 1095

V5(1) GCTGAATTGAACCACTTCTTGACCCTAGTAGGTGTCAGCTTTGACGAAAAAGGCAATCAC 1095

V6(3) GCTGAATTGAACCACTTCTTGACCCTAGTAGGTGTCAGCTTTGACGAAAAAGGCAATCAC 1095

V7(1) GCTGAATTGAACCACTTCTTGACCCTAGTAGGTGTCAGCTTTGACGAAAAAGGCAATCAC 1095

V8(1) GCTGAATTGAACCACTTCTTGACCCTAGTAGGTGTCAGCTTTGACGAAAAAGGCAATCAC 1095

V9(1) GCTGAATTGAACCACTTCTTGACCCTAGTAGGTGTCAGCTTTGACGAAAAAGGCAATCAC 1095

V10(2) GCTGAATTGAACCACTTCTTGACCCTAGTAGGTGTCAGCTTTGACGAAAAAGGCAATCAC 1095

V11(5) GCTGAATTGAACCACTTCTTGACCCTAGTAGGTGTCAGCTTTGACGAAAAAGGCAATCAC 1095

V12(2) GCTGAATTGAACCACTTCTTGACCCTAGTAGGTGTCAGCTTTGACGAAAAAGGCAATCAC 1095

************************************************************

TP02_0140(genomic) TGGATACTCAAAAACTCATTCGGTGAAGGCTGGGGAAACAAGGGATACCTACTGTTGACT 1260

XM_759616(mRNA) TGGATACTCAAAAACTCATTCGGTGAAGGCTGGGGAAACAAGGGATACCTACTGTTGACT 1260

V1(3) TGGATACTCAAAAA---------------------------------------------- 1109

V2(2) TGGATACTCAAAAA---------------------------------------------- 1109

V3(1) TGGATACTCAAAAA---------------------------------------------- 1109

V4(1) TGGATACTCAAAAA---------------------------------------------- 1109

V5(1) TGGATACTCAAAAA---------------------------------------------- 1109

V6(3) TGGATACTCAAAAA---------------------------------------------- 1109

V7(1) TGGATACTCAAAAA---------------------------------------------- 1109

V8(1) TGGATACTCAAAAA---------------------------------------------- 1109

V9(1) TGGATACTCAAAAA---------------------------------------------- 1109

V10(2) TGGATACTCAAAAA---------------------------------------------- 1109

V11(5) TGGATACTCAAAAA---------------------------------------------- 1109

V12(2) TGGATACTCAAAAA---------------------------------------------- 1109

**************

TP02_0140(genomic) CGCAATAGCAAGGAATACAAAGATGATTGTGGATTGAC***CTCCTTCGCAGTGTACGCAGTT*** 1320

XM_759616(mRNA) CGCAATAGCAAGGAATACAAAGATGATTGTGGATTGACCTCCTTCGCAGTGTACGCAGTT 1320

V1(3) ------------------------------------------------------------ 1109

V2(2) ------------------------------------------------------------ 1018

V3(1) ------------------------------------------------------------ 1109

V4(1) ------------------------------------------------------------ 1107

V5(1) ------------------------------------------------------------ 1107

V6(3) ------------------------------------------------------------ 1109

V7(1) ------------------------------------------------------------ 1109

V8(1) ------------------------------------------------------------ 1109

V9(2) ------------------------------------------------------------ 1109

V10(2) ------------------------------------------------------------ 1109

V11(5) ------------------------------------------------------------ 1109

V12(2) ------------------------------------------------------------ 1109

TP02_0140(genomic) ***TAA***AGATCAAAAATTGATATTTAATTATAATTGTACCACAAATTCTGTCCAAAGCTTTAG 1380

XM_759616(mRNA) TAAAGATCAAAAATTGATATTTAATTATAATTGTACCACAAATTCTGTCCAAAGCTTTAG 1380

V1(3) ------------------------------------------------------------ 1109

V2(2) ------------------------------------------------------------ 1018

V3(1) ------------------------------------------------------------ 1109

V4(1) ------------------------------------------------------------ 1107

V5(1) ------------------------------------------------------------ 1107

V6(3) ------------------------------------------------------------ 1109

V7(1) ------------------------------------------------------------ 1109

V8(1) ------------------------------------------------------------ 1109

V9(1) ------------------------------------------------------------ 1109

V10(2) ------------------------------------------------------------ 1109

V11(5) ------------------------------------------------------------ 1109

V12(2) ------------------------------------------------------------ 1109

Primer regions are in bold, underlined and italicized text.

^1^ Include X.1 variants.

**Table S10b: Tp8 protein variant Clustal alignment**

XP_764709 MLGNHVMGSNSPHIKILSSVTFLHIAKMEEVENVKVDALERVDTESVLNYDTVLEKKPLR 60

V1(3) -----------------------------------VDALERVDTESVLNYDTVLEKKPLR 25

V2(1) -----------------------------------VDALERVDTESVLNYDTVLEKKPLR 25

V3(1) -----------------------------------VDALERVDTESVLNYDTVLEKKPLR 25

V4(2) -----------------------------------VDALERVDTESVLNYDTVLEKKPLR 25

V5(1) -----------------------------------VDALERVDTESVLNYDTVLEKKPLR 25

V6(5)^1^ -----------------------------------VDALERVDTESVLNYDTVLEKKPLR 25

V7(10)^1^ -----------------------------------VDALERVDTESVLNYDTVLEKKPLR 25

*************************

XP_764709 SSVASFFKRYSAVLVILTAVLLFTFTFAAIALSSGRSAIRKNRELLSVEFEKLQFDNFVT 120

V1(3) SSVASFFKRYSAVLVILTAVLLFTFTFAAIALSSGRSAIRKNRELLSVEFEKLQFDNFVT 85

V2(1) SSVASFFKRYSAVLVILTAVLLFTFTFAAIALSSGRSAIRKNRELLSVEFEKLQFDNFVT 85

V3(1) SSVASFFKRYSAVLVILTAVLLFTFTFAAIALSSGRSAIRKNRELLSVEFEKLQFDNFVT 85

V4(2) SSVASFFKRYSAVLVILTAVLLFTFTFAAIALSSGRSAIRKNRELLSVEFEKLQFDNYVT 85

V5(1) SSVASFFKRYSAVLVILTAVLLFTFTFAAIALSSGRSAIRKNRELLSVEFEKLQFDNFVT 85

V6(5) SSVASFFKRYSAVLVILTAVLLFTFTFAAIALSSGRSAIRKNRELLSVEFEKLQFDNFVT 85

V7(10) SSVASFFKRYSAVLVILTAVLLFTFTFAAIALSSGRSAIRKNRELLSVEFEKLQFDNFVT 85

************************************************************

XP_764709 IKGEREEDFPKMVAEVLYKVAVEFDPKEEALIYVQFNDFNKQHDKKHNNYRHKKTSYTNF 180

V1(3) IKGEREEDFPKMVAEVLYKVAVEFDPKEEALIYVQFNDFNKQHDKKHNNYRHKKTSYTNF 145

V2(1) IKGEREEDFPKMVAEVLYKVAVEFDPKEEALIYVQFNDFNKQHDKKHNNYRHKKTSYTNF 145

V3(1) IKGEREEDFPKMVAEVLYKVAVEFDPKEEALIYVQFNDFNKQHDKKHNNYRHKKTSYTNF 145

V4(2) IKGEREEDFPKMVAEVLYKVAVEFDPKEEALIYVQFNDFNKQHDKKHNNYRHKKTSYTNF 145

V5(1) IKGEREEDFPKMVAEVLYKVAVEFDPKEEALIYVQFNDFNKQHDKKHNNYRHKKTSYTNF 145

V6(5) IKGEREEDFPKMVAEVLYKVAVEFDPKEEALIYVQFNDFNKQHDKKHNNYRHKKTSYTNF 145

V7(10) IKGEREEDFPKMVAEVLYKVAVEFDPKEEALIYVQFNDFNKQHDKKHNNYRHKKTSYTNF 145

************************************************************

XP_764709 RNNLNDINEHNAKPNLSYTKNMNHFGDISSKDFMKRYTKKVLLNLPKDHVSTYNNNRPMS 240

V1(3) RNNLNDINEHNAKPNLSYTKNMNHFGDISSKDFMKRYTKKVLLNLPKDHVSTYNNNRPMS 205

V2(1) RNNLNDINEHNAKPNLSYTKSMNHFGDISSKDFMKRYTKKVLLNLPKDHVSTYNNNRPMS 205

V3(1) RDNLNDINEHNAKPNLSYTKSMNHFGDISSKDFMKRYTKKVLLNLPKDHVSTYNNNRPMS 205

V4(2) RNNLNDINEHNAKPNLSYTKSMNHFGDISSKDFMKRYTKKVLLNLPKDHASTYNNNRPMS 205

V5(1) RNNLNDINEHNAKPNLSYTKSMNHFGDISSKDFMKRYTKKVLLNLPKDHASTYNNNRPMS 205

V6(5) RNNLNDINEHNAKPNLSYTKSMNHFGDISSKDFMKRYTKKVLLNLPKDHVSTYNNNRPMS 205

V7(10) RNNLNDINEHNAKPNLSYTKSMNHFGDISSKDFMKRYTKKVLLNLPKDHVSTYNNNRPMS 205

*.******************.****************************.**********

XP_764709 VDLRSHGVLTPVKCQEENELSWPYSVVAVAESFVKKTSQKTVSLSEKQLVDCVTDKKSAN 300

V1(3) VDLRSHGVLTPVKCQEENELSWPYSVVAVAESFVKKTSQKTVSLSEKQLVDCVTDKKSAN 265

V2(1) VDLRSHGVLTPVKCQEENELSWPYSVVAVAESFVKKTSQKTVSLSEKQLVDCVTDKKSVN 265

V3(1) VDLRSHGVLTPVKCQEENELSWPYSVVAVAESFVKKTSQKTVSLSEKQLVDCVTDKKSAN 265

V4(2) VDLRSHGVLTPVKCQEENELSWPYSVVAVAESFVKKTSQKTVSLSEKQLVDCVTDKKSAN 265

V5(1) VDLRSHGVLTPVKCQEENELSWPYSVVAVAESFVKKTSQKTVSLSEKQLVDCVTDKKSAN 265

V6(5) VDLRSHGVLTPVKCQEENELSWPYSVVAVAESFVKKTSQKTVSLSEKQLVDCVTDKKSAN 265

V7(10) VDLRSHGVLTPVKCQEENELSWPYSVVAVAESFVKKTSQKTVSLSEKQLVDCVTDKKSAN 265

**********************************************************.*

XP_764709 NPFLGYKYLKDLGLFESELVDKSTTKCPALEGERFKVPSYSYSYEPDLVALLLNAGPLTV 360

V1(3) NPFLGYKYLKDLGLFESELVDKSTTKCPALEGERFKVPSYSYSYEPDLVALLLNAGPLTV 325

V2(1) NPFLGYKYLKDLGLFESELVDKSATKCPALEGERFKVPSYSYSYEPDLVALLLNAGPLTV 325

V3(1) NPFLGYKYLKDLGLFESELVDKSTTKCPALEGERFKVPSYSYSYEPDLVALLLNAGPLTV 325

V4(2) NPFLGYKYLKDLGLFESELVDKSTTKCPALEGERFKVPSYSYSYEPDLVALLLNAGPLTV 325

V5(1) NPFLGYKYLKDLGLFESELVDKSTTKCPALEGERFKVPSYSYSYEPDLVALLLNAGPLTV 325

V6(5) NPFLGYKYLKDLGLFESELVDKSTTKCPALEGERFKVPSYSYSYEPDLVALLLNAGPLTV 325

V7(10) NPFLGYKYLKDLGLFESELVDKSATKCPALEGERFKVPSYSYSYEPDLVALLLNAGPLTV 325

***********************:************************************

XP_764709 PVAVSEDWQFYADGTLDVCGAELNHFLTLVGVSFDEKGNHWILKNSFGEGWGNKGYLLLT 420

V1(3) PVAVSEDWQFYADGTLDVCGAELNHFLTLVGVSFDEKGNHWILK---------------- 369

V2(1) PVAVSEDWQFYADGTLDVCGAELNHFLTLVGVSFDEKGNHWILK---------------- 369

V3(1) PVAVSEDWQFYADGTLDVCGAELNHFLTLVGVSFDEKGNHWILK---------------- 369

V4(2) PVAVSEDWQFYADGTLDVCGAELNHFLTLVGVSFDEKGNHWILK---------------- 369

V5(1) PVAVSEDWQFYADGTLDVCGAELNHFLTLVGVSFDEKGNHWILK---------------- 369

V6(5) PVAVSEDWQFYADGTLDVCGAELNHFLTLVGVSFDEKGNHWILK---------------- 369

V7(10) PVAVSEDWQFYADGTLDVCGAELNHFLTLVGVSFDEKGNHWILK---------------- 369

********************

Eptiope region highlighted in grey.

**Table S11a: Tp9 nucleotide variant Clustal alignment**

TP02_0140 ***ATGAATGTTCTAACTACTGG***AATAATTTTATACTCATTCTATCTTTCCATTTGTA-TGGA

XM_760370 ATGAATGTTCTAACTACTGGAATAATTTTATACTCATTCTATCTTTCCATTTGTA-TGGA

V1(3) ---------------------ATAATTTTATACTCATTCTATCTTTCCATTTGTA-TGGA

V2(3) ------------------------------------------------ATTTGTA-TGGA

V3(2) ------------------------------------------------ACTTCTA-AGGA

V4(3) ------------------------------------------------------------

V5(1) ---------------------ATAATTTTATACTCATTTTACACATGCCTTTGTA-TGGA

V6(2) ------------------------------------------------GTTTGTA-AGGA

V7(1) -------------------------------------------------CTTGCAGTGGA

V8(1) ----------------------------AATAATTTTATACTCATTCTATCTTTC-ATTG

V9(1) ------------------------------------------------CTTGCAG-TGGA

V10(1) ------------------------------------------------GCTGCTG-AGGA

V11(1) ------------------------------------------------------------

V12(1) ------------------------------------------------ATTTGTA-TGGA

V13(1) ------------------------------------------------GCTGCTG-AGGA

V14(1) ------------------------------------------------TTTAGTG-TGGA

V15(1) ------------------------------------------------------------

V16(1) ---------------------------GTCAGACTGGTCGTACGATGCATTTGTC-TGGA

V17(1) ---------------------------------------------------GTTA-CAGT

V18(2) ---ATGAATGTTCTAACTACTGGTATAATTTTATACTCATTCTACATGTCCATTT-GTAT

V19(1) --------------------AATAATTTTATTCTCATTTTACACATGCCTTTGTA-TGGA

V20(1) ---ATGAATGTTCTAACTACTGGAATAATTTTATACTCATTCTATCTTTCCATTT-GTAT

V21(1) --------------------AATAATTTTATACTCATTCTATCTTTCCATTTGTA-TGGA

V22(1) ATGAATGTTCTAACTACTGGAATAATTTTATACTCATTCTATCAATGTATTTGTA-TGGA

V23(1) --------------------AATAATTTTATACTCATTCTACCAATGCATTTGTA-TGGA

V24(1) --------------------AATAATTTTGTACTCATTCTACACATGCCTTTGTA-TGAA

V25(1) --------------------AATAATTTTGTACTCATTCTACACATGCCTTTGTA-TGAA

V26(1) --------------------AATAATTTTATACTCATTCTACATGTCCATTTGTA-TGGA

V27(1) --------------------AATAATTTTATACTCATTCTATCTTTCCATTTGTA-TGGA

V28(1) ATGAATGTTCTAACTACTGGAATAATTTTATACTCATTCTATCTTTCCATTTGTA-TGGA

TP02_0140 TCCTGATGATGATGTATTTGGATTCGATCCTGATGACGATGGAGACGGAAGCATGCTCCC

XM_760370 TCCTGATGATGATGTATTTGGATTCGATCCTGATGACGATGGAGACGGAAGCATGCTCCC

V1(3) TCCTGATGATGATGTATTTGGATTCGATCCTGATGACGATGGAGACGGAAGCATGCTCCC

V2(3) TTCTGGTG--AAGAA----GAAGATGATGTTTTCGGTGCTGAAGGAGGTAGCATGCTACC

V3(2) TTCTGGTG--ATGAT----GATGATGATGTTTTCGGTGCTGCGGGAGGTAGCATGCAACC

V4(3) -TCTGGTG--ATGAT----GATGATGATGTTTTCGGTGCTGCGGGAGGTAGCATGCTACC

V5(1) TCCTGATG--AT----------GATGATGCTTTCGGTGATGACGGAGGTAGCATGCTACC

V6(2) TACTGGTG--ATG-------ATGATGATGTTTTCGGTGATGACGGAGGTAGCATGCTAAC

V7(1) TTCTGATG--ATGAT----GATGATGATGTTTTCGGTGCTGCGGGAGGTAGCATGCTACC

V8(1) TATGGATACTGTGAT----GATGATGATGTTTTCGGTGATGACGCGGATAGCATGCTCCC

V9(1) TTCTGATG--ATGAT----GATGATGATGTTTTCGGTGCTGCGGGAGGTAGCATGCTACC

V10(1) TCCTGGTG--AAGAA----GAAGATGATGTTTTCGGTGCTGAAGGAGGTAGCATGCAACC

V11(1) -------------------------GATGTTTTCGGTGCTGAAGGAGGTAGCATGCTACC

V12(1) TTCTGGTG--AAGAA----GAAGATGATGTTTTCGGTGCTGAAGGAGGTAGCATGCTACC

V13(1) TCCTGGTG--AAGAA----GAAGATGATGTTTTCGGTGCTGAAGGAGGTAGCATGCAACC

V14(1) TCCTGGTG--AAGAA----GAAGATGATGTTTTCGGTGCTGAAGGAGGTAGCATGCTACC

V15(1) -----------------------------------------------------------C

V16(1) TTCTGGTG--AAGAA----GAAGATGATGTTTTCGGTGCTGAAGGAGGTAGCATGCTCCC

V17(1) GGATCCTG--ATGAT----AATGATGATGTTTTCGGTGATGACGAAGGTAGCATGCTCCC

V18(2) GGATCCTG--ATGAT----AATGATGATGTTTTCGGTGATGACGAAGGTAGCATGCTCCC

V19(1) TTCTGATG---------------ATGATGTTTTCGGTGCTGACGACGGTAGCATGCTACC

V20(1) GGATACTG--GTGAT----GATGATGATGTTTTCGGTGCTCCGGGAGGTAGCATGCTACC

V21(1) TCCTGATGATGATGTATTTGGATTCGATCCTGATGACGATGGAGACGGAAGCATGCTCCC

V22(1) TTCTGGTG--ATGAT----GATGATGATGTTTTCGGTGCTGCGGGAGGTAGCATGCTACC

V23(1) TTCTGGTG--ATGAT----GATGATGATGTTTTCGGTGCTGCGGGAGGTAGCATGCTACC

V24(1) TTCTGACG--ATGAC----GATGATGATGCTTTCGGTGCTGAAGGAGGTAGCATGCTCCC

V25(1) TTCTGACG--ATGAC----GATGATGATGCTTTCGGTGCTGAAGGAGGTAGCATGCTCCC

V26(1) TACTGATG--AAGAA----GATGATGATGTTTTCGGAGATCCAGGAGGTAGCATGCTACC

V27(1) TACTGGTG--ATG-------ATGATGATGTTTTCGGTGCTGACGGAGGTAGCATGCTCCC

V28(1) TACTGGTG--ATG-------ATGATGATGTTTTCGGTGCTGACGGAGGTAGCATGCTCCC

TP02_0140 TCCGCACCAAAGAAGCTCTATGTTTCGTGATGATCTCGGATCATCATTTACCTCAGGTTA

XM_760370 TCCGCACCAAAGAAGCTCTATGTTTCGTGATGATCTCGGATCATCATTTACCTCAGGTTA

V1(3) TCCGCACCAAAGAAGCTCTATGTTTCGTGATGATCTCGGATCATCATTTACCTCAGGTTA

V2(3) TCCACGCCAAAGAAGCTCTATGTTTGGTGAGCCTCTCGGATCATTACATACAGGAGGTTA

V3(2) TCCACGCCAAAGAAGCCCTATGTTTAGTGGTGGTCTCGGATCATCATTCACCTCAGGTTA

V4(3) TCCACGCCAAAGAAGCCCTATGTTTAGTGGTGGTCTCGGATCATCATTCACCTCAGGTTA

V5(1) TGAGCACAAACCATTCTCTATGATTAGCGGTGGTCTAGGATCATCACATACCGGAGGTTA

V6(2) TCCACACCAAAGAAGCTCTATGTTTCGTGATGATCTCGGATCATCATTTACCTCAGGTTA

V7(1) TCCACGCCAAAGAAGCCCTATGTTTAGTGGTGGTCTCGGATCATCATTCACCTCAGGTTA

V8(1) TAAACACCAAAGAAGCTCCGTGTTTGGTAGTGGTCTCGGATCAACATTTACCTCAGGTTA

V9(1) TCCACGCCAAAGAAGCTCTATGTTTAGTGAGCCTCTTGGATCAACATTTACCTCAGGTTA

V10(1) TCCACACCAAAGAAGCTCTATGCTTGGTAGTGGTCTCGGATCAACAATTACCTCAGGTTA

V11(1) TCCACGCCAAAGAAGCTCTATGTTTGGTGAGCCTCTCGGATCATTACATACAGGAGGTTA

V12(1) TCCACGCCAAAGAAGCTCTATGTTTGGTGAGCCTCTCGGATCATTACTTACCGGAGGTTA

V13(1) TCCACACCAAAGAAGCTCTATGCTTGGTAGTGGTCTCGGATCAACATTTACCTCAGGTTA

V14(1) TCCACACCAAAGAAGCTCTATGCTTGGTAGTGGTCTCGGATCAACATTTACCTCAGGTTA

V15(1) TTCACACCAAAGAAGCTCTATGCTTGGTAGTGGTCTCGGATCAACATTTACCTCAGGTTA

V16(1) TAAACACCAAAGAAGCTCTATGCTTGGTAGTGGTCTCGGATCAACATTTACCTCAGGTTA

V17(1) TAAACACCAAAGAAGCTCTATGCTTGGTAGTGGTCTCGGATCAACATTTACCTCAGGTTA

V180(2) TAAACACCAAAGAAGCTCTATGCTTGGTAGTGGTCTCGGATCAACATTTACCTCAGGTTA

V19(1) TCCACACCAAAGAAGCTCTATGTTTAGTGAGCCTCTCGGATCATTACATACCGGAGGTTA

V20(1) TCCACGCCAAAGAAGCTCTGTGTTTAGTGGTGGTATAGGATCAACATTTACCTCAGGTTA

V21(1) TCCGCACCAAAGAAGCTCCATCTTTAGTAGTGATCTAGGATCAACATTTACCACAGGTTA

V22(1) TCCACGCCAAAGAAGCCCTATGTTTAGTGGTGGTCTCGGATCATCATTCACCTCAGGTTA

V23(1) TCCACGCCAAAGAAGCCCTATGTTTAGTGGTGGTCTCGGATCATCATTCACCTCAGGTTA

V24(1) TAGGCACAAAAGAAGCTCTATGTTTAGTGAGCCTCTTGGATCAACATTTACCTCAGGTTA

V25(1) TAGGCACAAAAGAAGCTCTATGTTTAGTGAGCCTCTTGGATCAACATTTACCTCAGGTTA

V26(1) TCCACGCCAAAGAAGCTCCGTGTTTAGTGGTGGTCTCGGATCAACATTTACCTCAGGTTA

V27(1) TAGGCACCAAAGAAGCTCCGTGTTTAGTGGTGGTCTAGGAGAAACATTTACCTCAGGTTA

V28(1) TAGGCACCAAAGAAGCTCCGTGTTTAGTGGTGGTCTAGGAGAAACATTTACCTCAGGTTA

* ** ***

TP02_0140 TACTAAACAAGATTTGGATGCCAAATTTCCAGGTATGAAAAAGAGTAAGGGGCCCAAAGA

XM_760370 TACTAAACAAGATTTGGATGCCAAATTTCCAGGTATGAAAAAGAGTAAGGGGCCCAAAGA

V1(3) TACTAAACAAGATTTGGATGCCAAATTTCCAGGTATGAAAAAGAGTAAGGGGCCCAAAGA

V2(3) TGGTGATGAAGAATTTGAAAATAAATTTAAAAGTATGGGAATTGGTAAGAAACCCAAAGA

V3(2) TACTAGACAAGAGTTGGATGCAAAATTTCCAGGTATGAAAAAGGGTAAGGGATCCAAAGA

V4(3) TACTAGACAAGAGTTGGATGCAAAATTTCCAGGTATGAAAAAGGGTAAGGGATCCAAAGA

V5(1) TGATGATGATGAATTTCAACGAAAATTTCAACGTATGGGAATTGGTAAGAAACCCAAAGA

V6(2) TACTAGACAAGAGTTGGATGCAAAATTTCCAAATTTGAAAGCTCGTAGAGGGCCTATAGG

V7(1) TACTAGACAAGAGTTGGATGCAAAATTTCCAGGTATGAAAAAGGGTAAGGGATCCAAAGA

V8(1) CAGTGATCAAGAATTGGAGGAGAAATTTCAACGTATGGGAATTGGTAAGAAACCCAAAGA

V9(1) CAGTGATGATGAATTTGAAGAGAAATTTAAGCGTATGGGAATGAAGAAGAAACCCAAAGA

V10(1) CAGTGATCAAGAATTTGACGAGAAATTTCAACGTATGGGAATTGGTAAGAAACCCAAAGA

V11(1) TGGTGATGAAGAATTTGAAAATAAATTTAAAAGTATGGGAATTGGTAAGAAACCCAAAGA

V12(1) TGGTGATGAAGAATTTGAAAATAAATTTAAAAGTATGGGAATTGGTAAGAAACCCAAAGA

V13(1) CAGTGATCAAGAATTTGACGAGAAATTTCAACGTATGGGAATTGGTAAGAAACCCAAAGA

V14(1) CAGTGATCAAGAATTTGACGAGAAATTTCAACGTATGGGAATTGGTAAGAAACCCAAAGA

V15(1) CAGTGATCAAGAATTTGACGAGAAATTTCAACGTATGGGAATTGGTAAGAAACCCAAAGA

V16(1) CAGTGATCAAGAATTTGACGAGAAATTTAAACATATGGGAATTGGTAAGAAACCCAAAGA

V17(1) CAGTGATCAAGAATTTGACGAGAAATTTCAACGTATGGGAATTGGTAAGAAACCCAAAGA

V18(2) CAGTGATCAAGAATTTGACGAGAAATTTCAACGTATGGGAATTGGTAAGAAACCCAAAGA

V191(1) TGGTGATGAAGAATTTGAAAATAAATTTAAACATATGAGAATTGGTAAGAAACCCAAAGA

V20(1) CAGTGATAAAGAATTTGAGGAGAAATTTCAACGTATGGGAATTGGTAAGAAACCCAAAGA

V21(1) TACTTGCTGGGATTTGGAAAATAAATTTCCGGGTATGAAAAAGGGTAAGGGGCATAAAGA

V22(1) TACTAGACAAGAGTTGGATGCAAAATTTCCAGGTATGAAAAAGGGTAAGGGATCCAAAGA

V23(1) TACTAGACAAGAGTTGGATGCAAAATTTCCAGGTATGAAAAAGGGTAAGGGATCCAAAGA

V24(1) CAGTGATGATGAATTTGAAGAGAAATTTAAGCGTATGGGAATGAAGAAGAAACCCAAAGA

V25(1) CAGTGATGATGAATTTGAAGAGAAATTTAAGCGTATGGGAATGAAGAAGAAACCCAAAGA

V26(1) CAGTGATCAAGAATTTGACGAGAAATTTAAACATATGAGAATTGGTAAGAAACCCAAAGA

V27(1) CAGTGATCAAGAATTTGACGAGAAATTTAAACATATGGGAATTGGTAAGAAACCCAAAGA

V28(1) CAGTGATCAAGAATTTGACGAGAAATTTAAACATATGGGAATTGGTAAGAAACCCAAAGA

* * * * * ** * *

TP02_0140 CAAAG-------GGAAACCTCA------TCCTACAAAGCCTGTCAAGAGTACCTTAATGC

XM_760370 CAAAG-------GGAAACCTCA------TCCTACAAAGCCTGTCAAGAGTACCTTAATGC

V1(3) CAAAG-------GGAAACCTCA------TCCTACAAAGCCTGTCAAGAGTACCTTAATGC

V2(3) CAAAG-------GGAAACCTCA------TCCTACAAAGCCTGTCAAGAGTACCTTAATGC

V3(2) CAAAG-------GGCAACCTAA------GCTTCCAAAGCCCGTCAAAAGTACCTTAATGC

V4(3) CAAAG-------GGCAACCTAA------GCTTCCAAAGCCCGTCAAAAGTACCTTAATGC

V5(1) CAAATTA-----GGAAGGACAA---------ACCAACCTCAACCGGGAGCTGCA------

V6(2) CAAACCC-----AGAGGCCAAC---------CTCAAACTCATCAAGCAGCTGGC------

V7(1) CAAAGCCCCGTGGAACAACCCA-CCTCAACCTCCAAAGCCTGTTAAAAGTACCTTAATTC

V8(1) CAAAG-------GGAAACCTCA------TCCTACAAAGCCTGTCAAGAGTACCTTAATGC

V9(1) CAAACTC-----GGAAGGACAA---------ACCAACCTCAACCAGGAACAGTA------

V10(1) CAAAG-------GGAAACCTCA------TCCTACAAAGCCCGTCAAAAGTACCTTAATGC

V11(1) CAAAG-------GGAAACCTCA------TCCTACAAAGCCTGTCAAGAGTACCTTAATGC

V12(1) CAAAG-------GGAAACCTCA------TCCTACAAAGCCTGTCAAGAGTACCTTAATGC

V13(1) CAAAG-------GGAAACCTCA------TCCTACAAAGCCCGTCAAAAGTACCTTAATGC

V14(1) CAAAG-------GGAAACCTCA------TCCTACAAAGCCCGTCAAAAGTACCTTAATGC

V15(1) CAAAG-------GGAAACCTCA------TCCTACAAAGCCCGTCAAAAGTACCTTAATGC

V16(1) CAAAG-------GGAAACCTCA------TCCTACAAAGCCCGTCAAAAGTACCTTAATGC

V17(1) CAAAG-------GGAAACCTCA------TCCTACAAAGCCCGTCAAAAGTACCTTAATGC

V180(2) CAAAG-------GGAAACCTCA------TCCTACAAAGCCCGTCAAAAGTACCTTAATGC

V19(1) CAAACTC-----GGAAGGACAA---------ACCAACCTCAACCGGGAGCACCT------

V20(1) CAAAG-------GGAAACCTCA------TCCTACAAAGCCCGTCAAAAGTACCTTAATGC

V21(1) CAAAG-------GACAACCTCAACCTCAGCCTCCAAAGCCCGTCAAAAGTACCTTAATGC

V22(1) CAAGGCCCCGTGGAACAACCCA-CCTCAACCTCCAAAGCCTGTTAAAAGTACCTTAATTC

V23(1) CAAAGCCCCGTGGAACAACCCA-CCTCAACCTCCAAAGCCTGTTAAAAGTACCTTAATTC

V24(1) CAAACTC-----GGAAGGACAA---------ACCAACCTCAACCAGGA------------

V25(1) CAAACTC-----GGAAGGACAA---------ACCAACCTCAACCAGGAACAGTA------

V26(1) CAAACTC-----GGAAGGACAA---------ACCAACCTCAACAAGGAGCACCT------

V27(1) CAAACTC-----AAAAGAACAA---------ACCAACCTCCACCAGGTCCTACT------

V28(1) CAAACTC-----AAAAGAACAA---------ACCAACCTCCACCAGGTCCTACT------

***

TP02_0140 CAGGAAATGAT-GGTCA--AACAG------------GAGCTACTGGAGGTTAT---CCAG

XM_760370 CAGGAAATGAT-GGTCA--AACAG------------GAGCTACTGGAGGTTAT---CCAG

V1(3) CAGGAAATGAT-GGTCA--AACAG------------GAGCTACTGGAGGTTAT---CCAG

V2(3) CAGGAAATGAT-GGTCA--AACAG------------GAGCTACTGGACCCTATGGACAAA

V3(2) CAGAGGGTGGTCAATCAGGAGCAGTTGGAGGTTACGGAGCTACTGGACCCTATGGACAAG

V4(3) CAGAGGGTGGTCAATCAGGAGCAGTTGGAGGTTACGGAGCTACTGGACCCTATGGACAAG

V5(1) --GGAGG---------------------------TTATCCAGGCGGTTACCCAACTCAAC

V6(2) --GGTTATGG------------------------TCAACCACAAGG--------------

V7(1) CAGGTGATGATGTTCCACAAG---------------GAGCCGTTGGACCTTACGG---AG

V8(1) CAGAGGCTGGTCAATCAGGAGCAGTTGGAGGTTACGGAGCTGCTGGACCCTATGGACAAA

V9(1) --GGAGG---------------------------CTATGGG-------------------

V10(1) CAGAGGCTGGTCAATCAGGAGCAGTTGGAGGTTACGGAGCTGCTGGACCCTATGGACAAA

V11(1) CAGGAAATGAT-GGTCA--AACAG------------GAGCTACTGGACCCTATGGACAAA

V12(1) CAGGAAATGAT-GGTCA--AACAG------------GACCTACTGGACCCTATGGACAAA

V13(1) CAGAGGCTGGTCAATCAGGAGCAGTTGGAGGTTACGGAGCTGCTGGACCCTATGGACAAA

V14(1) CAGAGGCTGGTCAATCAGGAGCAGTTGGAGGTTACGGAGCTGCTGGACCCTATGGACAAA

V15(1) CAGAGGCTGGTCAATCAGGAGCAGTTGGAGGTTACGGAGCTGCTGGACCCTATGGACAAA

V16(1) CAGAGGCTGGTCAATCAGGAGCAGTTGGAGGTTACGGAGCTGCTGGACCCTATGGACAAA

V17(1) CAGAGGCTGGTCAATCAGGAGCAGTTGGAGGTTACGGAGCTGCTGGACCCTATGGACAAA

V180(2) CAGAGGCTGGTCAATCAGGAGCAGTTGGAGGTTACGGAGCTGCTGGACCCTATGGACAAA

V19(1) --GGGGCATATCCTCCACCTGCAG------------CAGCTGGCGGATTCGGT-------

V20(1) CAGAGGCTGGTCAATCAGGAGCAGTTGGAGGTTACGGAGCTGCTGGACCCTATGGACAAA

V21(1) CAGAATCCGGTCGTCCACAAG---------------GTGCCGCTGCAGGTTAT---CCAG

V22(1) CAGGTGATGATGTTCCACAAG---------------GAGCCGTTGGACCTTACGG---AG

V23(1) CAGGTGATGATGTTCCACAAG---------------GAGCCGTTGGACCTTACGG---AG

V24(1) ------------------------------------------------------------

V25(1) --GGAGG---------------------------CTATGGG-------------------

V26(1) --GGAGCATATCCTCCACCTGCAGCCGCTGGACCTTACGGAGGCGGTTACCCAAGTCAAC

V27(1) --GGGGGCTA------------------------TGGGCAACCTGGTTATCCAAGTCAAC

V28(1) --GGGGGCTA------------------------TGGGCAACCTGGTTACCCAAGTCAAC

TP02_0140 GTGGTTATCCCACTCAA---GGA---------------------------------CC--

XM_760370 GTGGTTATCCCACTCAA---GGA---------------------------------CC--

V1(3) GTGGTTATCCCACTCAA---GGA---------------------------------CC--

V2(3) CTGGTTATCCCACTCAACCTGG----------------T-----------------CC--

V3(2) CAGGTTATGTCGGTCAACCGGG------------------AGCTGCTGGAAATTATCCAG

V4(3) CAGGTTATGTCGGTCAACCGGG------------------AGCTGCTGGAAATTATCCAG

V5(1) CAGGAGCTGCAGGAGGTTATCC--------------------------------------

V6(2) ------------------------------------------------------------

V7(1) GCGGTTATCCAAGTCAA---GGA---------------------------------CC--

V8(1) CAGGTTATGTCGGTCAACCTGGTCCTTATGGTCAAACTGGAGCTGCTGGAGGTTATCAAG

V9(1) ------------------------------------------------------------

V10(1) CAGGTTATGTCGGTCAACCTGG----------------T-----------------CC--

V11(1) CTGGTTATCCCACTCAACCTGG----------------T-----------------CC--

V12(1) CTGGTTATCCCACTCAACCTGG----------------T-----------------CC--

V13(1) CAGGTTATGTCGGTCAACCTGG----------------T-----------------CC--

V14(1) CAGGTTATGTCGGTCAACCTGG----------------T-----------------CC--

V15(1) CAGGTTATGTCGGTCAACCTGG----------------T-----------------CC--

V16(1) CAGGTTATGTCGGTCAACCTGG----------------T-----------------CC--

V17(1) CAGGTTATGTCGGTCAACCTGG----------------T-----------------CC--

V18(2) CAGGTTATGTCGGTCAACCTGG----------------T-----------------CC--

V19(1) --GGCTATCCGAGTCAATCAGG--------------------------------------

V20(1) CAGGTTATGTCGGTCAACCTGG----------------T-----------------CC--

V21(1) CAGGTTATCCCACTCAA---GGA---------------------------------CC--

V22(1) GCGGTTATCCAAGTCAA---GGA---------------------------------CC--

V23(1) GCGGTTATCCAACTCAA---GGA---------------------------------CC--

V24(1) ------------------------------------------------------------

V25(1) ------------------------------------------------------------

V26(1) AAGGAGCACCTGGAGCATATCCTCCACCTGCAGCCGCTGGACCTTACGGAGGCGGTTACC

V27(1) CACAAGCTGCAGGACCTTACGG---------------------------AGGCGGTTACC

V28(1) CACAAGCTGCAGGACCTTACGG---------------------------AGGCGGTTACC

TP02_0140 ----TTAT---GGTCAACCAGG------------------AGCTACTGGACCCTATGGAC

XM_760370 ----TTAT---GGTCAACCAGG------------------AGCTACTGGACCCTATGGAC

V1(3) ----TTAT---GGTCAACCAGG------------------AGCTACTGGACCCTATGGAC

V2(3) ----TTAC---GGTCAACCGGG------------------ACCCGTTGG-----------

V3(2) GTGGTTATGCTGGTCAACCTGGTCCTTATGGTCAACCAGGAGCTACTGGACCCTATGGAC

V4(3) GTGGTTATGCTGGTCAACCTGGTCCTTATGGTCAACCAGGAGCTACTGGACCCTATGGAC

V5(1) ----------------AGGCGGTTACCCAACTCAACCAGGAGCTGCAGGAGGTTATCCA-

V6(2) -------------AGGACCTGGGGTATATCCTCCACCGGCAGCAGCTGGACCTTACGGA-

V7(1) ----TTAT---GGTCAACCAGG---------------------AGCTGGACCCTATGGAC

V8(1) GTGGTTATCCCACTCAACCTGGTCCTTATGGTCAAACAGGAGCCACTGGACCCTATGGAC

V9(1) --------------------------------------------------------CAA-

V10(1) ----TTAT---GGTCAAACGGGTTACGTTGGTCAACCGGGAGCCGTCGGACCTTACGGA-

V11(1) ----TTAC---GGTCAACCGGG------------------AGCCACTGGAGGTTATCAA-

V12(1) ----TTAC---GGTCAACCGGG------------------AGCCACTGGAGGTTATCAA-

V13(1) ----TTAT---GGTCAAACGGGTTACGTTGGTCAACCGGGAGCCGTTGGACCTTACGGA-

V14(1) ----TTAT---GGTCAAACGGGTTACGTTGGTCAACCGGGAGCCGTTGGACCTTACGGA-

V15(1) ----TTAT---GGTCAAACGGGTTACGTTGGTCAACCGGGAGCCGTCGGACCTTACGGA-

V16(1) ----TTAT---GGTCAAACGGGTTACGTT-------------------------------

V17(1) ----TTAT---GGTCAAACGGGTTACGTTGGTCAACCGGGAGCCGTTGGACCTTACGGA-

V18(2) ----TTAT---GGTCAAACGGGTTACGTTGGTCAACCGGGAGCCGTTGGACCTTACGGA-

V19(1) ----------------ACAAGG---------------------CGTTTATCCTCCACAA-

V20(1) ----TTAT---GGTCAAACGGGTTACGTTGGTCAACCGGGAGCCGTTGGACCTTACGGA-

V21(1) ----TTAT---GGTCAACCAGG------------------AGCTAGTGGAGGTTATCCA-

V22(1) ----TTAT---GGTCAATCAGG---------------------AGCTGGACCTTATGGAC

V23(1) ----TTAT---GGTCAACCAGG---------------------AGCTGGACCCTATGGAC

V24(1) ------------------------------------------------------------

V25(1) --------------------------------------------------------CAA-

V26(1) CAAGTCAACAAGGAGCACCTGGAGCATATCCTCCACCTGCAGCCGCTGGACCTTACGGA-

V27(1) CAAGTCAACCACAAGGACCTGGGGTATATCCTCCACCAGGAGCAGCTGGACCTTACGGA-

V28(1) CAAGTCAACCACAAGGACCTGGGGTATATCCTCCACCAGGAGCAGCTGGACCTTACGGA-

TP02_0140 AAGCAGGTTACGTTGGTCAACCGGGAGCCGTTGGACCTTACGGTCAACCAGGAGCT----

XM_760370 AAGCAGGTTACGTTGGTCAACCGGGAGCCGTTGGACCTTACGGTCAACCAGGAGCT----

V1(3) AAGCAGGTTACGTTGGTCAACCGGGAGCCGTTGGACCTTACGGTCAACCAGGAGCT----

V2(3) ------------------------------------------------------------

V3(2) AAGCAGGTTATGTCGGTCAACC---------GGGAGCTGCTGGAAATTATCCAGGT----

V4(3) AAGCAGGTTATGTCGGTCAACC---------GGGAGCTGCTGGAAATTATCCAGGT----

V5(1) --GGCGGTTACCCAAGTCAACA---------AGGAGCTGCAGGAGG--------------

V6(2) --GGTGGTTACCCAAGTCAACC---------AGGAGCATCTGGAGGTTATGGAGGCG---

V7(1) AAACGGGTTACGTTGGACCTTACGGAGGCGGTTATCCA----------------------

V8(1) AAGCAGGTTATGTCGGTCAACC---------GGGAGCTGCTGGAGGTTATCCAGGT----

V9(1) --CCTGGTTATCCATCTCAACC---------ACAAGCAGCTGGAGC--------------

V10(1) --GGGGGTTATCCAACTCAA------------GGACCTTATGGTCAACCAGGAGCT----

V11(1) --GGTGGTTATCCAACTCAACC---------TGGTCCTTATCCTCCACCAGGAGCTA---

V12(1) --GGTGGTTATCCAACTCAACC---------TGGTCCTTATCCTCCACCAGGAGCTA---

V13(1) --GGGGGTTATCCAACTCAA------------GGACCTTATGGTCAACCAGGAGCT----

V14(1) --GGGGGTTATCCAACTCAA------------GGACCTTATGGTCAACCAGGAGCT----

V15(1) --GGGGGTTATCCAACTCAA------------GGACCTTATGGTCAACCAGGAGCT----

V16(1) ------------------------------------------------------------

V17(1) --GGGGGTTATCCAACTCAA------------GGACCTTATGGTCAACCAGGAGCT----

V18(2) --GGGGGTTATCCAACTCAA------------GGACCTTATGGTCAACCAGGAGCT----

19(1) -----GGTTACGTTGGACAA---------------CC-----------------------

V20(1) --GGGGGTTATCCAACTCAA------------GGACCTTATGGTCAACCAGGAGCT----

V21(1) --GCTGGTTATCCCACTCAACC---------TGGTCCTTATGTTCAACCAGGAGCTA---

V22(1) AAACGGGTTACGTTGGTCAACC---------TGGTCCTTATC------------------

V23(1) AAACGGGTTACGTTGGACCTTACGGAGGCGGTTATCCAACTCAAGGACCTTATGGTCAAC

V24(1) -----------------------------------GCATCTGGAGG--------------

V25(1) --CCTGGTTATCCATCTCAACC---------ACAAGCAGCTGGAGC--------------

V26(1) --GGCGGTTACCCAACTCAACA---------AGGAGCACCTGGAGCATATCC--------

V27(1) --GGCGGTTACCCAAGTCAACC---------ACAAGGACCTGGGGTATATCC--------

V28(1) --GGCGGTTACCCAAGTCAACC---------ACAAGGACCTGGGGTATATCC--------

TP02_0140 --------GTACCCTATGGACAAACTGGTTATCCCACTCAACCAGGAGCAGCTGGAGGTT

XM_760370 --------GTACCCTATGGACAAACTGGTTATCCCACTCAACCAGGAGCAGCTGGAGGTT

V1(3) --------GTACCCTATGGACAAACTGGTTATCCCACTCAACCAGGAGCAGCTGGAGGTT

V2(3) ------------------------------------------------------------

V3(2) --------GGTTATGATGGTCAACCTGGTCCTTATGGTCAACCAGGAGCTACTGGACCCT

V4(3) --------GGTTATGATGGTCAACCTGGTCCTTATGGTCAACCAGGAGCTACTGGACCCT

V5(1) ----------------------------------------------------------TT

V6(2) ------------------------------------GTGCATCAGGTGGCGCTGGACCTT

V7(1) ------------------------------------------------------------

V8(1) --------GGTTATGCTGGTCAACCTGGTCCTTATGGTCAACCAGGAGCTACTGGACCCT

V9(1) ----------------------------------------------------------TT

V10(1) --------GGACCCTATGGACAAACTGGTTATCCCACTCAACCAGGAGCAGCTGGAGGTT

V11(1) ------CTGGACCCTATGGACAAACTAGTTATCCCACTCAACCAGGAGCTGCTGGAGGTT

V12(1) ------CTGGACCCTATGGACAAACTAATTATCCCACTCAACCAGGAGCTGCTGGAGGTT

V13(1) --------GGACCCTATGGACAAACTGGTTATCCCACTCAACCAGGAGCAGCTGGAGGTT

V14(1) --------GGACCCTATGGACAAACTGGTTATCCCACTCAACCAGGAGCAGCTGGAGGTT

V15(1) --------GGACCCTATGGACAAACTGGTTATCCCACTCAACCAGGAGCAGCTGGAGGTT

V16(1) ------------------------------------------------------------

V17(1) --------GGACCCTATGGACAAACTGGTTATCCCACTCAACCAGGAGCAGCTGGAGGTT

V18(2) --------GGACCCTATGGACAAACTGGTTATCCCACTCAACCAGGAGCAGCTGGAGGTT

V19(1) -------------------------------------------AGGAGCACCTGGGGCCT

V20(1) --------GGACCCTATGGACAAACTGGTTATCCCACTCAACCAGGAGCAGCTGGAAATT

V21(1) ------CTGGACCCTATCGACAAACGGGTTATCCCACTCAACCAGGAGCTGCTGGAGGTT

V22(1) ------------------------------------CTCCACCAGGAGCTACTGGACCCT

V23(1) CAGGAGCTGGACCCTATGGACAAACGGGTTATCCCACTCAACCAGGAGCAGCTGGAGGTT

V24(1) ----------------------------------------------------------TT

V25(1) ----------------------------------------------------------TT

V26(1) -------------------------------------TCCACCTGCAGCCGCTGGACCTT

V27(1) -------------------------------------TCCACCAGGAGCAGCTGGACCTT

V28(1) -------------------------------------TCCACCAGGAGCAGCTGGACCTT

TP02_0140 AT---CCAGGTGGTTATGCTGGCCAACCTGGTCCTTATGGT-------------------

XM_760370 AT---CCAGGTGGTTATGCTGGCCAACCTGGTCCTTATGGT-------------------

V1(3) AT---CCAGGTGGTTATGCTGGCCAACCTGGTCCTTATGGT-------------------

V2(3) ------------------------------------------------------------

V3(2) ATGGACAAGCAGGTTATGTCGGTCAACCGGGAGC--------------------------

V4(3) ATGGACAAGCAGGTTATGTCGGTCAACCGGGAGC--------------------------

V5(1) ATC---CAGGCGGTTACCCAAGTCAACAAGGAGGACCTGGAGCAT---------------

V6(2) ACG---GAGGTGGTTACCCAAGTCAACCAGGAGCATCTGGAGGTT---------------

V7(1) --------GGTGGTTATGCTGGTCAACCGGGAGC--------------------------

V8(1) ATGGACAAGCAGGTTATGTCGGTCAACCGGGAGCTGCTGGAGGTTATCCAGGTGGTTATG

V9(1) ACG---GAGGCGGTTATCCAACTCAACAAGGAGGACCTGGTTATC---------------

V10(1) AT---CCAGGTGGTTATACTGGTCAACCAGGAGC--------------------------

V11(1) AT---CCAGGTGGTTATCCCACTCAACCAGGAGC--------------------------

V12(1) AT---CCAGGTGGTTATCCCACTCAACCAGGAGC--------------------------

V13(1) AT---CCAGGTGGTTATACTGGCCAACCAGGAGC--------------------------

V14(1) AT---CCAGGTGGTTATACTGGTCAACCAGGAGC--------------------------

V15(1) AT---CCAGGTGGTTATACTGGTCAACCAGGAGC--------------------------

V16(1) ------------------------------------------------------------

V17(1) AT---CCAGGTGGTTATACTGGTCAACCAGGAGC--------------------------

V18(2) AT---CCAGGTGGTTATACTGGTCAACCAGGAGC--------------------------

V19(1) ATG---GAGGCGGTTATCCTAGTCAGCAAGGGGC--------------------------

V20(1) AT---CCAGGTGGTTATGCTGGTCAACCGGGAGC--------------------------

V21(1) AT---CCGGCTGGTTATGCTGGTCAACCGGGAGC--------------------------

V22(1) ATGGACAAACGGGTTACGTTGGTCAACCGGGAGC--------------------------

V23(1) AT---CCAGGTGGTTATGCTGGTCAACCGGGAGC--------------------------

V24(1) ACG---GAGGTGGTTACCCAA---------------------------------------

V25(1) ACG---GAGGCGGTTATCCAACTCAACAAGGAGGACCTGGTTATC---------------

V26(1) ACG---GAGGCGGTTACCCAAGTCAACAAGGAGCACCTGGAGCAT---------------

V27(1) ACG---GAGGCGGTTACCCAAGTCAACCACAAGGACCTGGGGTAT---------------

V28(1) ACG---GAGGCGGTTACCCAAGTCAACCACAAGGACCTGGGGTAT---------------

TP02_0140 ---------------CAACCAGGAGCTACTGGACCCTATGGACAAGC--AGGTTATGTCG

XM_760370 ---------------CAACCAGGAGCTACTGGACCCTATGGACAAGC--AGGTTATGTCG

V1(3) ---------------CAACCAGGAGCTACTGGACCCTATGGACAAGC--AGGTTATGTCG

V2(3) ------------------------------------------------------------

V3(2) ----------------ATCTGGAGGTTACGGAGCCGGTTATTCTGGTCAAGGTGGTTATC

V4(3) ----------------ATCTGGAGGTTACGGAGCCGGTTATTCTGGTCAAGGTGGTTATC

V5(1) ---------------ATCC------TCCACCGGCAGCAGCTGGAGGTCAAGGGCCGGCTT

V6(2) ---------------ATGGAGGCGGTGCATCAGGTGGCGCTGGACCTTACGGAGGTGGTT

V7(1) ----------------ATCTGGAGGTTACGGAGCCGGTTATTCTGGTCAAGGTGGTTATC

V8(1) CTGGTCAACCGGGAGCATCTGGAGGTTACGGAGCCGGTTATTCTGGTCAAGGTGGTTATC

V9(1) ---------------CACC------TCCACCGGCAGCAGCTGGAGCTCAAGGACATGCTT

V10(1) ----------------TACTGGAGGTTACGGAGCTGGTTATTCAGGTCAAGGTGGTTATC

V11(1) ----------------GGTTGGAGGCTACGGAGGCGGTTATTCAGGTCAAGGTGGTTATC

V12(1) ----------------GGTTGGAGGCTACGGACGCGGTTATTCAGGTCAAGGTGGTTATC

V13(1) ----------------TACTGGAGGTTACGGAGCTGGTTATTCAGGTCAAGGTGGTTATC

V14(1) ----------------TACTGGAGGTTACGGAGCTGGTTATTCAGGTCAAGGTGGTTATC

V15(1) ----------------TACTGGAGGTTACGGAGCTGGTTATTCAGGTCAAGGTGGTTATC

V16(1) ------------------------------------------------------------

V17(1) ----------------TACTGGAGGTTACGGAGCTGGTTATTCAGGTCAAGGTGGTTATC

V180(2) ----------------TACTGGAGGTTACGGAGCTGGTTATTCAGGTCAAGGTGGTTATC

V19(1) ----------------AACTGGCGGATTCGG---TGGCTATCCGAGTCAATCAGG-----

V20(1) ----------------ATCTGGAGGTTACGGAGCCGGTTATTCTGGTCAAGGTGGTTATC

V21(1) ----------------ATCTGGAGGTTACGGAGCCGGTTATTCTGGTCAAGGTGGTTATC

V22(1) ----------------ATCTGGAGGTTACGGAGCTGGTTATTCAGGTCAAGGTGGTTATC

V23(1) ----------------ATCTGGAGGTTACGGAGCAGGTTATTCTGGTCAAGGTGGTTATC

V24(1) ------------------------------------------------------------

V25(1) ---------------CACC------TCCACCGGCAGCAGCTGGAGCTCAAGGACATGCTT

V26(1) ---------------ATCC------TCCACCTGCAGCCGCTGGAGCTCAAGGGCCGGCTT

V27(1) ---------------ATCC------TCCACCAGGAGCAGCTGGACCTTACGGAGGCGGTT

V28(1) ---------------ATCC------TCCACCAGGAGCAGCTGGACCTTACGGAGGCGGTT

TP02_0140 GTCAACCGGGAGCTGCTGGGGGTTATCCAGGTGGTTATCCTCCACCAGGAGCA-------

XM_760370 GTCAACCGGGAGCTGCTGGGGGTTATCCAGGTGGTTATCCTCCACCAGGAGCA-------

V1(3) GTCAACCGGGAGCTGCTGGGGGTTATCCAGGTGGTTATCCTCCACCAGGAGCA-------

V2(3) ------------------------------------------------------------

V3(2) CTCCACAAGGTTATGGACAACCCGGGCAAGGTCCTTATCCTCCGCCAGGAGCA-------

V4(3) CTCCACAAGGTTATGGACAACCCGGGCAAGGTCCTTATCCTCCGCCAGGAGCA-------

V5(1) ATCCTCCAGGCTATGGTCAACCGGGAGCAGCTGGAGGTTATGGAGGCGGTGCATCAGGTG

V6(2) ACCCA---------AGTCAACCAGGAGCATCTGGAGGTTATGGAGGCGGTGCATCAGGTG

V7(1) CTCCACAAGGTTACGTACAACCCGGGCAAGGTCCTTATCCTCCGTCAGGACCATCTGGAG

V8(1) CTCCACAAGGTTATGGACAACCCGGGCAAGGTCCTTATCCTCCGCCAGGAGCA-------

V9(1) ATCCTCCAGGCTATGGTCAACCGGTAGCAGCTGGAGGTTATGGAGGCGGTGCATCAGGTG

V10(1) CTCCACAAGGCTATGGACAACCCGGGCAAGGTCCTTATCCTCCGCCAGGACCATCTGGGG

V11(1) CACCACAAGGCTATGGACAACCCGGGAAAGGTCCTTATCCTCCGCCAGGACCATCTGGGG

V12(1) CACCACAAGGCTATGGACAACCCGGGAAAGGTCCTTATCCTCCGCCAGGACCATCTGGGG

V13(1) CTCCACAAGGCTATGGACAACCCGGGCAAGGTCCTTATCCTCCGCCAGGACCATCTGGGG

V14(1) CTCCACAAGGCTATGGACAACCCGGGCAAGGTCCTTATCCTCCGCCAGGACCATCTGGGG

V15(1) CTCCACAAGGCTATGGACAACCCGGGCAAGGTCCTTATCCTCCGCCAGGACCATCTGGGG

V16(1) ------------------------------------------------------------

V17(1) CTCCACAAGGTTATGGACAACCCGGGCAAGGTCCTTATCCTCCGTCAGGACCATCTGGAG

V180(2) CTCCACAAGGTTATGGACAACCCGGGCAAGGTCCTTATCCTCCGTCAGGACCATCTGGAG

V19(1) ----ACAAGGC---GTTTATCCTCCACAAGGTCCTTATGCTCCACCCGGAGCAGCCGGAG

V20(1) CTCCACAAGGTTATGGACAACCCGGGCAAGGTCCTTATCCTCCGCCAGGAGCA-------

V21(1) CTCCACAAGGTTATGGACAACCCGGGCAAGGTCCTTATCCTCCGTCAGGACCATCTGGAG

V22(1) CTCCACAAGGTTATGGACAACCCGGGCAAGGTCCTTATCCTCCGCCAGGAGCA-------

V23(1) CTCCACAAGGTTACGTACAACCCGGGCAAGGTCCTTATCCTCCGTCAGGACCATCTGGAG

V24(1) ---------------GTCAACCAGGAGCATCTGGAGGTTATGGAGGCGGTGCATCAGGTG

V25(1) ATCCTCCAGGCTATGGTCAACCGGTAGCAGCTGGAGGTTATGGAGGCGGTGCATCAGGTG

V26(1) ATCCTCCAGGCTATGGTCAACCGGTAGCATCTGGAGGTTATGGAGGCGGTGCATCAGGTG

V27(1) ACCCA---------AGTCAACCACAAGGACCTGGGGTATATCC------TCCACCAGGAG

V28(1) ACCCA---------AGTCAACCACAAGGACCTGGGGTATATCC------TCCACCAGGAG

TP02_0140 --------------GCTGGAGGCTATGGAGGCGGCCCACCCGGTC---CAGGTCAGCCAA

XM_760370 --------------GCTGGAGGCTATGGAGGCGGCCCACCCGGTC---CAGGTCAGCCAA

V1(3) --------------GCTGGAGGCTATGGAGGCGGCCCACCCGGTC---CAGGTCAGCCAA

V2(3) ------------------------------------------------------------

V3(2) --------------GCTGGAGGCTATGGAGGTGGCCCACCCGGTC---CAGGTCAGCCAA

V4(3) --------------GCTGGAGGCTATGGAGGTGGCCCACCCGGTC---CAGGTCAGCCAA

V5(1) GC------------GCTGGAGGCTACGGAGGTGGTTATCCAGGTCAACAAGGTGGTCCAC

V6(2) GC------------GCTGGAGGTTACGGAGGCGGTTATCCAAGTCAACCAGGTGGTCCAC

V7(1) GCCATGGAGCAGGTGCTGGAGGCTATGGAGGCGGCGCACCCGGTC---CAGGTCAGCCAA

V8(1) --------------GCTGGAGGCTATGGAGGTGGCCCACCCGGTC---CAGGTCAGCCAA

V9(1) GC------------GCTGGAGGTTACGGAGGTGGTTATCCAAGTCAACCAGGTGGTCCAC

V10(1) GCTATGGAGCAGGTGCTGGAGGCTATGGAGGTAGTGCACCCGGTC---CAGGTCACCCAA

V11(1) GCTATGGATCAGGTGCTGGAGGCTATGGAGGCGGCGAACCGGGTT---CAGGTCACCCAA

V12(1) GCTATGGATCACGTGCTGGAGGCTATGGAGGCGGCAAACCGGGTT---CAGGTCACCCAA

V13(1) GCTATGGAGCAGGTGCTGGAGGCTATGGAGGTAGTGCACCCGGTC---CAGGTCACCCAA

V14(1) GCTATGGAGCAGGTGCTGGAGGCTATGGAGGCGGCGAACCGGGTC---CAGGTCACCCAA

V15(1) GCTATGGAGCAGGTGCTGGAGGCTATGGAGGTAGTGCACCCGGTC---CAGGTCACCCAA

V16(1) ------------------------------------------------------------

V17(1) GCCATGGAGCAGGTGCTGGAGGCTATGGAGGCGGCGCGCCTGGTC---CAGGTCAGCCAA

V180(2) GCCATGGAGCAGGTGCTGGAGGCTATGGAGGCGGCGCGCCTGGTC---CAGGTCAGCCAA

V19(1) GCTATGGTGGCGGTGCTGGCGGTTATGGCGGTGGCGCATCAGGTC---CAGGTCAGCCGA

V20(1) --------------GCTGGAGGCTATGGAGGTGGCCCACCCGGTC---CAGGTCAGCCAA

V21(1) GCCCTGGAGCAGGTGCTGGAGGTTATGGAGGTGGCGCACCCGGTC---CAGGTCAGCCAA

V22(1) --------------GCTGGAGGATATGGAGGTGGCCCACCCGGTC---CAGGTCAGCCAA

V23(1) GCCATGGAGCAGGTGCTGGAGGCTATGGAGGCGGCGCACCCGGTC---CAGGTCAGCCAA

V24(1) GC------------GCTGGACCTTACGGAGGTGGTTACCCAAGTCAACCAGGTGGTCCAC

V25(1) GC------------GCTGGAGGTTACGGAGGTGGTTATCCAAGTCAACCAGGTGGTCCAC

V26(1) GC------------GCTGGAGGTTACGGAGGTGGTTATCCAAGTCAACCAGGTGGTCCAC

V27(1) CA------------GCTGGACCTTACGGAGGCGGTTACCCAAGTCAACCAGGTGGTCCAC

V28(1) CA------------GCTGGACCTTACGGAGGCGGTTACCCAAGTCAACCAGGTGGTCCAC

TP02_0140 GCGGTCCCCCTGGTCAAGCTCCTGGCAATATGCAATTGTTGAAAA-TAGATGTAAAAAAT

XM_760370 GCGGTCCCCCTGGTCAAGCTCCTGGCAATATGCAATTGTTGAAAA-TAGATGTAAAAAAT

V1(3) GCGGTCCCCCTGGTCAAGCTCCTGGCAATATGCAATTGTTGAAAA-TAGATGTAAAAAAT

V2(3) ------------------------------------------------------------

V3(2) GCGGTCCCCCTGGTCAAGCTCCTGGCAATATGCAATTGTTGAAAA-TAGATGTAA-----

V4(3) GCGGTCCCCCTGGTCAAGCTCCTGGCAATATGCAATTGTTGAAAA-TAGATGTAAAAAAT

V5(1) AAGGAGCGGCTGGAC---CTCCTGGCGAGCCGATTATGGTGAAAA-TGGATGCAAAAAGC

V6(2) AAGGAGCAGCTGGAC---CTCCCGGCCAGCCGATTATGATGAGAA-TTGATGGGAAGAAC

V7(1) GTGGCCCACCTGGTCAAGCCCCTGGAGGGA---ATATGTTGAAAA-TAGATGCAAAAAGT

V8(1) GCGGTCCCCCTGGTCAAGCTCCTGGCAATATGCAATTGTTGAAAA-TAGATGTAAAAAAT

V9(1) AAGGAGCAGCTGGAC---CTCCTGGCCAGCCGATTATGATGAAAA-TTGATGTGAAGAAC

V10(1) GCGGTCCCACTGGCCATGCTTCTGGCGGGA---ATTTGTTGAAAA-TAGATGTAAAAAAT

V11(1) GCGGTCCCACTGGCCATGCTTCTGGCGGGA---ATTTGTTGAAAA-TAGATGTAAAAAAT

V12(1) TCGATCCCACTGGCCATGCTTCTGGCGGGA---ATTTGTTGAAGA-TAGATGTAAAAAAT

V13(1) GCGGTCCCACTGGCCATGCTTCTGGCGGGA---ATTTGTTGAAAA-TAGATGTAAAAAAT

V14(1) GCGGTCCCACTGGCCATGCTTCTGGCGGGA---ATTTGTTGAAAAATAGATGTAAAAAAT

V15(1) GCGGTCCCACTGGCCATGCTTCTGGCGGGA---ATTTGTTGAAAA-TAGATGTAAAAAAT

V16(1) ------------------------------------------------------------

V17(1) GTGGCCCAACTGGTCAAACCCATGGAGGGA---ATATGTTGACAA-TAGATGCAAAAACT

V18(2) GTGGCCCAACTGGTCAAACCCATGGAGGGA---ATATGTTGACAA-TAGATGCAAAAACT

V19(1) CCGGTCCTCCTGGCCATACTCCTGGGGTGA---ATTTGTTGACAA-TAGATGCAAAAACT

V20(1) GCGGTCCCCCTGGTCAAGCTCCTGGCAATATGCAATTGTTGAAAA-TAGATGTAAAAAAT

V21(1) GTGGCCCACCTAGTCAATCTCCTGATGGGAAAAATTTAGGCCCTG-TAGATGGAAAAACA

V22(1) GCGGTCCCCCTGGTCAAGCCCCTGGCAATATGCAATTGTTGACAA-TAGATGCAAAAACT

V23(1) GTGGCCCACCTGGTCAAGCCCCTGGAGGGA---ATATGTTGAAAA-TAGATGCAAAAAGT

V24(1) AAGGAGCGGCTGGAC---CTCCTGGAGAGCCGATTATGCTGAAAA-TGGATGCAAAGAAC

V25(1) AAGGAGCAGCTGGAC---CTCCTGGCCAGCCGATTATGATGAAAA-TTGATGTGAAGAAC

V26(1) AAGGAGCAGCTGGAC---ATCCTGGCCAGCCGATTATGATGAAAA-TTGATGCGAAGAAC

V27(1) AAGGAGCAACTGGAC---CTCCTGGCCAGCCGATTATGATGAAAA-TTGATGCGAAGAAC

V28(1) AGGGAGCAACTGGAC---CTCCTGGCCAGCCGATTATGATGAAAA-TTGATGCGAAGAAC

TP02_0140 CCAAACAATGGTCCAAATATTATTTTTGAAGAATATATAGCCGGAAAGCCAGAACGTCGT

XM_760370 CCAAACAATGGTCCAAATATTATTTTTGAAGAATATATAGCCGGAAAGCCAGAACGTCGT

V1(3) CCAAACAATGGTCCAAATATTATTTTTGAAGAATATATAGCCGGAAAGCCAGAACGTCGT

V2(3) ------------------------------------------------------------

V3(2) ------------------------------------------------------------

V4(3) CCAAACAATGGTCCAAATATTATTTTTGAAGAATATATAGCCGGAA--------------

V5(1) AGAGTAAAT---AGAGATATTAATCTTACTAAATTTACACT---GACAAATGGTAAAACT

V6(2) AGAAGAAAT---CCAAATACTAGACTTGATAGATTTACACT---ACCAGATGGTAGAACT

V7(1) CCAAACAATGGCAAAAATATTATTGTTGAAGAATTTAGAGCGGGTAGACCAGAACGTACT

V8(1) CCAAACAATGGTCCAAATATTATTTTTGAAGAATATATAGCCGGAAAGCCAGAACGTCGT

V9(1) AGAAGAAAC---CCAAATACTAAACTTGATAGATTTACACT---AACAGATGGTAGAACT

V10(1) CCAAACAATGGTCCAAA-------------------------------------------

V11(1) CCAAACAATGGTCCAAATATTATTTTTGAAGAATATATAGCCGGAAAGCCAGAACGTCGT

V12(1) CCAAACAATGGTCCAAATATTATTTTTGAAGAATATATACCCA-----------------

V13(1) CCAAACAATGGTCCAAA-------------------------------------------

V14(1) CCAAACAATGGTCCAAA-------------------------------------------

V15(1) CCAAACAATGGTCCAAATATTATTTTTGAAGAATATATAGCC------------------

V16(1) ------------------------------------------------------------

V17(1) AAACACAATGGCCCAAA-------------------------------------------

V18(2) AAACACAATGGCCCAAATATTATTGTTGACGAATATAGAGCCGGACCACAGGATCGTCGT

V19(1) AAACACAATGGCGCAAATATTATTGTTGACGAATATAGAGCCGGTCGACCAGAACGTATT

V20(1) CCAAACAATGGTCCAAATATTATTTTTGAAGAATATATAGCCGGAAAGCCAGAACGTCGT

V21(1) GGAAAAAACGATCCATTTGTTATTTTCGAAAGATTTAGGGCAGGACCACAAAATCGTGTT

V22(1) AAACACAATGGCGCAAATATTATTGTTGACGAATATAGAGCCGGTCGACCAGAACGTATT

V23(1) CCAAACAATGGCAAAAATATTATTGTTGAAGAATTTAGAGCGGGTAGACCAGAACGTACT

V24(1) AGAACAAAT---CGAAATATTAATCTTACTAAATTTACACT---ACCAAATGGTAAAACT

V25(1) AGAAGAAAC---CCAAATACTAAACTTGATAGATTTACACT---AACAGATGGTAGAACT

V26(1) AGAAGAAAT---CCAAATACTAAACTTGATAGATTTACACT---ATCAAATGGTGGAACT

V27(1) AGAAGAAAT---CCAAATACTAAACTTGATAGATTTACGCT---ATCAAATGGTGGAACT

V28(1) AGAAGAGAT---CCAAATACTAAACTTGATAGATTTACGCT---ATCAAATGGTGGAACT

TP02_0140 CACCGTCAGTTTACCCCTGTTCCTGGATGTGGAATTAATCAAGTAAATTATGACGGCGAA

XM_760370 CACCGTCAGTTTACCCCTGTTCCTGGATGTGGAATTAATCAAGTAAATTATGACGGCGAA

V1(3) CACCGTCAGTTTACCCCTGTTCCTGGATGTGGAATTAATCAAGTAAATTATGACGGCGAA

V2(3) ------------------------------------------------------------

V3(2) ------------------------------------------------------------

V4(3) ------------------------------------------------------------

V5(1) CAACATGTGTTTAGAGCTAATCCTGGACATGGAATTAAACAAGTAAATTATGACGGTGAT

V6(2) CAACATGTGTTTAGACCTAATCTTGGATATGCAATTAAACAAGTAAATTATGACGGTGAT

V7(1) CACCGTCAGTTTACCCCTGTTCCTGGATGTGGAATTAATCAAGTAAATTA----------

V8(1) CACCGTCAGTTTACCCCTGTTCCTGGATGTGGAATTAATCAAGTAAATTATGACGGCGAA

V9(1) CAACATGCGTTTAGAGCTAATCCTGGATATGCAATTAAACAAGTAAATTATGACGGTGAT

V10(1) ------------------------------------------------------------

V11(1) CACCGTCAGTTTACCCCTGTTCCTGGATGTGGAATTAATCAAGTAAATTATGACGGCGAA

V12(1) ------------------------------------------------------------

V13(1) ------------------------------------------------------------

V14(1) ------------------------------------------------------------

V15(1) ------------------------------------------------------------

V16(1) ------------------------------------------------------------

V17(1) ------------------------------------------------------------

V18(2) CACCGTCAGTTTGATCCTGTTCCTGGATGTGGAATTATTCAAGTAAATTATGACGGTCGC

V19(1) CACCGTCAGTTTGATCCTGTTCCTGGATGTGGAATTAATCAAGTAAATTATAATGGTAAA

V20(1) CACCGTCAGTTTACCCCTGTTCCTGGATGTGGAATTAATCAAGTAAATTATGACGGTGGT

V21(1) CACCATCAGTATACTCCCCGTAGTGGTTGTGGGTTCAATATGGTAAAATATTCAGGTGGA

V22(1) CACCGTCAGTTTGATCCCGTTCCTGGATGTGGAATTAATCAAGTAAATTATAATGGTCAA

V23(1) CACCGTCAGTTTACCCCTGTTCCTGGATGTGGAATTAATCAAGTAAATTATGACGGTCGC

V24(1) CAACATGTGTTTAGAGCTAATCCTGGACATGGAATTAAACAAGTAAATTATGACGGTGAT

V25(1) CAACATGCGTTTAGAGCTAATCCTGGATATGCAATTAAACAAGTAAATTATGACGGTGAT

V26(1) CAACATGTGTTTAGACCTGATCCTGGACATGGAATTAAACAAGTAAATTATGACGGTGAT

V27(1) CAACATGTGTTTAGACCTGATCCTGGACATGGAATTAAACAAGTAAATTATGACGGTGAT

V28(1) CAACATGTGTTTAGACCTGATCCTGGACATGGAATTAAACAAGTAAATTATGACGGTGAT

TP02_0140 AAAGTTTGGTCATTAGAAGTGGGTG---GCGATTATGCAGTAAAAGTTCTTGTTTTTCCT

XM_760370 AAAGTTTGGTCATTAGAAGTGGGTG---GCGATTATGCAGTAAAAGTTCTTGTTTTTCCT

V1(3) AAAGTTTGGTCATTAGAAGTGGGTG---GCGATTATGCAGTAAAAGTTCTTGTTTTTCCT

V2(3) ------------------------------------------------------------

V3(2) ------------------------------------------------------------

V4(3) ------------------------------------------------------------

V5(1) CCTGTTTGGTCAATGGATGGTAAT------AATTTTGGAACAGAAGTTCTTCTGGATCCT

V6(2) CGTGTTTGGTCAATGGATGAT---------------------------------------

V7(1) ------------------------------------------------------------

V8(1) AAAGTTTGGTCATTAGAAGTGGGT------GGCGATTATGCAGTAAAAGTTCTTGTTTTT

V9(1) CGTGTTTGGTCAATGGATGGTGGT------AATTTTGGAACAGAAGTTCTTGTGGATCCT

V10(1) ------------------------------------------------------------

V11(1) AAAGTTTGGT--------------------------------------------------

V12(1) ------------------------------------------------------------

V13(1) ------------------------------------------------------------

V14(1) ------------------------------------------------------------

V15(1) ------------------------------------------------------------

V16(1) ------------------------------------------------------------

V17(1) ------------------------------------------------------------

V18(2) AAAGTTTGGACAGCGAGTGGTCCC------GAACATGCAGTAAAAGTAGTTGTGTTTCCT

V19(1) CCAGTTTGGAAAATGATAGGTGAA------AAATATGCAGTAAAAGTAGTTCTGTTTCCT

V20(1) AACGTTTGGACAATGAACGATGAC------ATTCATGCATTAAAAGTAGTTGTGTTTCCT

V21(1) ATCGTTTGGATATTAGGGGGTGACGGTAGTAATTATGCAACAAAGGTTATTGTGCATCCT

V22(1) CCAGTTTGGAAAATGATAGGTGAA------AAATATGCAGTAACAGTAGTTTTGTTTCCT

V23(1) AAAGTTTGGTCATTAGAAGTGGGTGGCGATCATGCAGTAAAAGTTCTTGTGTTTCCTATA

V24(1) TTTGTTTGGTCAATGGATGGTGGT------AATTTTGGAACAGAAGTTCTTGTGGATCCT

V25(1) CGTGTTTGGTCAATGGATGGTGGT------AATTTTGGAACAGAAGTTCTTGTGGATCCT

V26(1) CGTGTTTGGTCAATGGATCGTGAT------AATTTTGGCACAGAAGTTCTTGTAGATCCT

V27(1) CCTGTTTGGTCAATGGATGGTAAT------AATTTTGGAACAGAAGTTCTTCTGGATCCT

V28(1) CCTGTTTGGTCAATGGATGGTAAT------AATTTTGGAACAGAAGTTCTTCTGGATCCT

TP02_0140 ATAGGGTTTAAAGAAAAAACTATTGAAATCACATTTATTGGCGGTGAAAAGGAAATCTAT

XM_760370 ATAGGGTTTAAAGAAAAAACTATTGAAATCACATTTATTGGCGGTGAAAAGGAAATCTAT

V1(3) ATAGGGTTTAAAGAAAAAACTATTGAAATCACATTTATTGGCGGTGAAAAGGAAATCTAT

V2(3) ------------------------------------------------------------

V3(2) ------------------------------------------------------------

V4(3) ------------------------------------------------------------

V5(1) ATAGGGTCTCCAGCAAAAAATATGACAATCAAACTGGCAAACGGTGATAAGGTTACTTTT

V6(2) ------------------------------------------------------------

V7(1) ------------------------------------------------------------

V8(1) CCTATAGGGTTTAAAGAAAAAACTATTGAAATCACATTTATTGGCGGTGAAAAGGAAATC

V9(1) ATAGGGTCTGGAGCAAAAACTATGACAATCAAACTGGCAAACGG----------------

V10(1) ------------------------------------------------------------

V11(1) ------------------------------------------------------------

V12(1) ------------------------------------------------------------

V13(1) ------------------------------------------------------------

V14(1) ------------------------------------------------------------

V15(1) ------------------------------------------------------------

V16(1) ------------------------------------------------------------

V17(1) ------------------------------------------------------------

V18(2) CTAGGATTTAAAGAAAAAACTATTGAAATCACATTTATTGGCGGTGAAAAGGCGATCTAT

V19(1) ATAGGATTTGAAGAAAAAACTATTGTAATCACAATGATAGGTGGTGAAAACGAGATCTAT

V20(1) ATAGGGTTTAAAGAAAAAACTATTGAAATCACATTTATTGGCGGTGAAAAGGAAATCTAT

V21(1) GTAGGGTTTAAAGAAAAAACTATTGAAATCACATTTATTGGCGGTGAAAAGGAGATCTAT

V22(1) ATAGGGTTTGAAGAAAAAACTATTGTAATCACAATGATAGGTGGTGAAAAGGAGATCTAT

V23(1) GGGTTTAAAGAAAAAACTATTGAAATCACATTTATTGGCGGTGAAAAGGAGATCTATAAA

V24(1) ATAGGGTCTGGAGCAAAAACTATGACAATCAAATTGGCAAACGGTGATAAGGTTATTCAT

V25(1) ATAGGGTCTGGAGCAAAAACTATGACAATCAAACTGGCAAACGGTGATAATGTTATTTAT

V26(1) ATAGGGTCTGGAGCAAAAACTATGACAATCAAAATGGTAAACGGTGATGAGGTTACTTTT

V27(1) ATAGGGTCTCCAGCAAAAAATATGACAATCAAAATGGTAAACGGTGATAAGGAAATCTAT

V28(1) ATAGG-TCTCCAGCAAAAA-TATGACA-TCAAATTTATTGGCGGTGAAAAGGAAATCTAT

TP02_0140 AAAAAGAAAGGG***CGTAATAAACCATGGACAAAACAATAA***

XM_760370 AAAAAGAAAGGGCGTAATAAACCATGGACAAAACAATAA

V1(3) AAAAAGAAAGGG---------------------------

V2(3) ---------------------------------------

V3(2) ---------------------------------------

V4(3) ---------------------------------------

V5(1) ATAAAGAAAGGG---------------------------

V6(2) ---------------------------------------

V7(1) ---------------------------------------

V8(1) TATAAAAAGAAAGGG------------------------

V9(1) ---------------------------------------

V10(1) ---------------------------------------

V11(1) ---------------------------------------

V12(1) ---------------------------------------

V13(1) ---------------------------------------

V14(1) ---------------------------------------

V15(1) ---------------------------------------

V16(1) ---------------------------------------

V17(1) ---------------------------------------

V18(2) AAAAAGCCAGGGCGTAATAAACCATGGACAAAACAATAA

V19(1) AAAAAGAAAGGG---------------------------

V20(1) AAAAAGAAAGGGCGTAATAAACCATGGACAAAACAATAA

V21(1) AAAAAGAAAGGG---------------------------

V22(1) AAAAAGAAAGGGCGTAATAAACCATGGACAAAACAATAA

V23(1) AAGAAAGGG------------------------------

V24(1) ACAAAGCCAGGA---------------------------

V25(1) ACAAAGCCAGGA---------------------------

V26(1) ACAAAGTCAGGA---------------------------

V27(1) AAAAAGAAAGGG---------------------------

V28(1) AAAAAGAAAGGGCGTAATAAACCATGGACAAAACAATAA

Primer regions are in bold, underlined and italicized text.

**Table S11b: Tp9 protein variant Clustal alignment**

TP02_0895 MNVLTTGIILYSFYLSICMDP---DDDVFGFDPDDDGDGSMLPPHQRSSMFRDDLGSSFT

V1(3) MNVLTTGIILYSFYLSICMDP---DDDVFGFDPDDDGDGSMLPPHQRSSMFRDDLGSSFT

V2(1) ----------------ICMDSGEEEDDVFGAE-----GGSMLPPRQRSSMFGEPLGSLLT

V3(1) --------------------------DVFGAE-----GGSMLPPRQRSSMFGEPLGSLHT

V4(3) ----------------ICMDSGEEEDDVFGAE-----GGSMLPPRQRSSMFGEPLGSLHT

V5(2) ----------------TSKDSGDDDDDVFGAA-----GGSMQPPRQRSPMFSGGLGSSFT

V6(2) MNVLTTGIILYSFYMSICMDP-DDNDDVFGDD-----EGSMLPKHQRSSMLGSGLGSTFT

V7(1) MNVLTTGIILYSFYMSICMDTDEEDDDVFGDP-----GGSMLPPRQRSSVFSGGLGSTFT

V8(1) ----------------LAVDSDDDDDDVFGAA-----GGSMLPPRQRSSMFSEPLGSTFT

V9(1) MNVLTTGIILFSFYTCLCMDS---DDDVFGAD-----DGSMLPPHQRSSMFSEPLGSLHT

V10(1) -------IILYSFYTCLCMDPDDDD--AFGDD-----GGSMLPEHKPFSMISGGLGSSHT

V11(1) MNVLTTGIILYSFYTCLCMNSDDDDDDAFGAE-----GGSMLPRHKRSSMFSEPLGSTFT

V12(1) MNVLTTGIILYSFYTCLCMNSDDDDDDAFGAE-----GGSMLPRHKRSSMFSEPLGSTFT

V13(1) MNVLTTGIILYSFYLSICMDTGDDDD-VFGAD-----GGSMLPRHQRSSVFSGGLGETFT

V14(1) MNVLTTGIILYSFYLSICMDTGDDDD-VFGAD-----GGSMLPRHQRSSVFSGGLGETFT

V15(2) ----------------AAEDPGEEEDDVFGAE-----GGSMQPPHQRSSMLGSGLGSTFT

V16(1) ----------------AAEDPGEEEDDVFGAE-----GGSMQPPHQRSSMLGSGLGSTIT

V17(2) ----------------VCKDTGDDDD-VFGDD-----GGSMLTPHQRSSMFRDDLGSSFT

V18(1) ----------------VTVDP-DDNDDVFGDD-----EGSMLPKHQRSSMLGSGLGSTFT

V19(1) ----------------LAVDSDDDDDDVFGAA-----GGSMLPPRQRSPMFSGGLGSSFT

V20(1) MNVLTTGIILYSFYQCICMDSGDDDDDVFGAA-----GGSMLPPRQRSPMFSGGLGSSFT

V21(1) MNVLTTGIILYSFYLSICMDTGDD-DDVFGAP-----GGSMLPPRQRSSVFSGGIGSTFT

V22(3) MNVLTTGIILYSFYQCICMDSGDDDDDVFGAA-----GGSMLPPRQRSPMFSGGLGSSFT

V23(1) ----------------FSVDPGEEEDDVFGAE-----GGSMLPPHQRSSMLGSGLGSTFT

V24(1) -------------------------------------------SHQRSSMLGSGLGSTFT

V25(1) MNVLTTGIILYSFYQCICMDSGDDDDDVFGAA-----GGSMLPPRQRSPMFSGGLGSSFT

V26(1) MNVLTTGIILYSFYLSICMD---PDDDVFGFDPDDDGDGSMLPPHQRSSIFSSDLGSTFT

V27(1) ---------VRLVVRCICLDSGEEEDDVFGAE-----GGSMLPKHQRSSMLGSGLGSTFT

*

TP02_0895 SGYTKQDLDAKFPGMKKSKGPKDKG----KPH--PTKPVKSTLMPG-----NDGQTGATG

V1(3) SGYTKQDLDAKFPGMKKSKGPKDKG----KPH--PTKPVKSTLMPG-----NDGQTGATG

V2(1) GGYGDEEFENKFKSMGIGKKPKDKG----KPH--PTKPVKSTLMP--------GNDGQTG

V3(1) GGYGDEEFENKFKSMGIGKKPKDKG----KPH--PTKPVKSTLMP--------GNDGQTG

V4(3) GGYGDEEFENKFKSMGIGKKPKDKG----KPH--PTKPVKSTLMP--------GNDGQTG

V5(2) SGYTRQELDAKFPGMKKGKGSKDKG----QPK--LPKPVKSTLMP------EGGQSGAVG

V6(2) SGYSDQEFDEKFQRMGIGKKPKDKG----KPH--PTKPVKSTLMP------EAGQSGAVG

V7(1) SGYSDQEFDEKFKHMRIGKKPKDK--------------LGRTNQPQQGAPGAYPPPAAAG

V8(1) SGYSDDEFEEKFKRMGMKKKPKDK--------------LGRTNQPQ---------PGTVG

V9(1) GGYGDEEFENKFKHMRIGKKPKDK--------------LGRTNQP---------QPGAPG

V10(1) GGYDDDEFQRKFQRMGIGKKPKDK--------------LGRTNQPQ---------PGAAG

V11(1) SGYSDDEFEEKFKRMGMKKKPKDK--------------LGRTNQPQ---------PGTVG

V12(1) SGYSDDEFEEKFKRMGMKKKPKDK--------------LGRTNQPQ---------PGASG

V13(1) SGYSDQEFDEKFKHMGIGKKPKDK--------------LKRTNQPP---------PGPTG

V14(1) SGYSDQEFDEKFKHMGIGKKPKDK--------------LKRTNQPP---------PGPTG

V15(2) SGYSDQEFDEKFQRMGIGKKPKDKG----KPH--PTKPVKSTLMP------EAGQSGAVG

V16(1) SGYSDQEFDEKFQRMGIGKKPKDKG----KPH--PTKPVKSTLMP------EAGQSGAVG

V17(2) SGYTRQELDAKFPNLKARRGPIGK--------------P--RGQPQ--------THQAAG

V18(1) SGYSDQEFDEKFQRMGIGKKPKDKG----KPH--PTKPVKSTLMP------EAGQSGAVG

V19(1) SGYTRQELDAKFPGMKKGKGSKDKAPWNNPPQ--PPKPVKSTLIP--------GDDVPQG

V20(1) SGYTRQELDAKFPGMKKGKGSKDKAPWNNPPQ--PPKPVKSTLIP--------GDDVPQG

V21(1) SGYSDKEFEEKFQRMGIGKKPKDKG----KPH--PTKPVKSTLMP------EAGQSGAVG

V22(3) SGYTRQELDAKFPGMKKGKGSKDKG----QPK--LPKPVKSTLMP------EGGQSGAVG

V23(1) SGYSDQEFDEKFQRMGIGKKPKDKG----KPH--PTKPVKSTLMP------EAGQSGAVG

V24(1) SGYSDQEFDEKFQRMGIGKKPKDKG----KPH--PTKPVKSTLMP------EAGQSGAVG

V25(1) SGYTRQELDAKFPGMKKGKGSKDKAPWNNPPQ--PPKPVKSTLIP--------GDDVPQG

V26(1) TGYTCWDLENKFPGMKKGKGHKDKG----QPQPQPPKPVKSTLMP------ESGRPQ---

V27(2) SGYSDQEFDEKFKHMGIGKKPKDKG----KPH--PTKPVKSTLMP------EAGQSGAVG

** ::: ** : : .* *

TP02_0895 GYPGGYPTQGPYGQPGATGPYGQAGYVGQ-----------PGAVGPYGQPGAV-PYGQTG

V1(3) GYPGGYPTQGPYGQPGATGPYGQAGYVGQ-----------PGAVGPYGQPGAV-PYGQTG

V2(1) ---PTGP-YGQTGYPTQPGPYGQPGATGG------YQGGYPTQPGPYPPPGATGPYGQTN

V3(1) ---ATGP-YGQTGYPTQPGPYGQPGATGG------YQGGYPTQPGPYPPPGATGPYGQTS

V4(3) ---ATGP-YGQTGYPTQPGPYGQPGPV---------------------------------

V5(2) GYGATGP-YGQAGYVGQPGAAGN------------YPGGYAGQPGPYGQPGATGPYGQAG

V6(2) GYGAAGP-YGQTGYVGQPGPYGQTGYVGQPGAVGPYGGGYPTQ-GPYGQPGAG-PYGQTG

V7(1) PYGGGYP--SQQGAPGAYPPPAAAGPYGGGYPSQQGAPGAYPPPAAAGPYGGGYPTQQGA

V8(1) GYG-----------------------------------------------QPGYPSQPQA

V9(1) AYPPPAAAGGFGGYPSQSGQG---------------------------------VYPPQG

V10(1) GYPGGYP--TQPGAAGGYPGGYPT------------------QPGAAGGYPGGYPSQQGA

V11(1) GYG-----------------------------------------------QPGYPSQPQA

V12(1) GYG---------------------------------------------------------

V13(1) GYGQPGY--PS-------------------------------QPQAAGPYGGGYPSQPQG

V14(1) GYGQPGY--PS-------------------------------QPQAAGPYGGGYPSQPQG

V15(2) GYGAAGP-YGQTGYVGQPGPYGQTGYVGQPGAVGPYGGGYPTQ-GPYGQPGAG-PYGQTG

V16(1) GYGAAGP-YGQTGYVGQPGPYGQTGYVGQPGAVGPYGGGYPTQ-GPYGQPGAG-PYGQTG

V17(2) GYGQPQG--GPGVYP---------------------------PPAAAGPYGGGYPSQPGA

V18(1) GYGAAGP-YGQTGYVGQPGPYGQTGYVGQPGAVGPYGGGYPTQ-GPYGQPGAG-PYGQTG

V19(1) ---AVGP-YGG-GYPSQ-GPYGQPG------------------AGPYGQTGYVGPYGG--

V20(1) ---AVGP-YGG-GYPSQ-GPYGQSG------------------AGPYGQTGYVGQPGPYP

V21(1) GYGAAGP-YGQTGYVGQPGPYGQTGYVGQPGAVGPYGGGYPTQ-GPYGQPGAG-PYGQTG

V22(3) GYGATGP-YGQAGYVGQPGAAGN------------YPGGYAGQPGPYGQPGATGPYGQAG

V23(1) GYGAAGP-YGQTGYVGQPGPYGQTGYVGQPGAVGPYGGGYPTQ-GPYGQPGAG-PYGQTG

V24(1) GYGAAGP-YGQTGYVGQPGPYGQTGYVGQPGAVGPYGGGYPTQ-GPYGQPGAG-PYGQTG

V25(1) ---AVGP-YGG-GYPTQ-GPYGQPG------------------AGPYGQTGYVGPYGGGY

V26(1) --GAAAG-Y-PAGYPTQ-GPYGQPGASGG------YPAGYPTQPGPYVQPGATGPYRQTG

V27(2) GYGAAGP-YGQTGYVGQPGPYGQTGYV---------------------------------

TP02_0895 YPTQP----------------------GAAGGYPG---------------------GYAG

V1(3) YPTQP----------------------GAAGGYPG---------------------GYAG

V2(1) YPTQP----------------------GAAGGYPG---------------------GYPT

V3(1) YPTQP----------------------GAAGGYPG---------------------GYPT

V4(3) ------------------------------------------------------------

V5(2) YVGQP----------------------GAAGNYPGGYDGQPGPYGQPGATGPYGQAGYVG

V6(2) YPTQP----------------------GAAGGYPG---------------------GYTG

V7(1) PGAYP--------------------PPAAAGPYGG---------------------GYPS

V8(1) -----------------------------AGAYGG---------------------GYPT

V9(1) YVGQP----------------------GAPGAYGG---------------------GYPS

V10(1) -----------------------------AGGYPG---------------------GYPS

V11(1) -----------------------------AGAYGG---------------------GYPT

V12(1) ----------------------------------G---------------------GYPS

V13(1) PGVYP--------------------PPGAAGPYGG---------------------GYPS

V14(1) PGVYP--------------------PPGAAGPYGG---------------------GYPS

V15(2) YPTQP----------------------GAAGGYPG---------------------GYTG

V16(1) YPTQP----------------------GAAGGYPG---------------------GYTG

V17(2) SGGYGGG------------------ASGGAGPYGG---------------------GYPS

V18(1) YPTQP----------------------GAAGGYPG---------------------GYTG

V19(1) ---------------------------GYPG-------------------------GYAG

V20(1) PP-------------------------GATGPYGQ--------------------TGYVG

V21(1) YPTQP----------------------GAAGNYPG---------------------GYAG

V22(3) YVGQP----------------------GAAGNYPGGYDGQPGPYGQPGATGPYGQAGYVG

V23(1) YPTQP----------------------GAAGGYPG---------------------GYTG

V24(1) YPTQP----------------------GAAGGYPG---------------------GYTG

V25(1) PTQGPYGQPGAGPYGQTGYPTQPGAAGGYPG-------------------------GYAG

V26(1) YPTQP----------------------GAAGGYPA---------------------GYAG

V27(2) ------------------------------------------------------------

TP02_0895 QPGPYGQPG---AT-GPYGQAGYVGQP----GAAGGYPGGYPPPG---------------

V1(3) QPGPYGQPG---AT-GPYGQAGYVGQP----GAAGGYPGGYPPPG---------------

V2(1) QPGAVGGYG---R--GYSGQGGYPPQ-----GYGQPGKGPYPPPG--------PSGGYGS

V3(1) QPGAVGGYG---G--GYSGQGGYPPQ-----GYGQPGKGPYPPPG--------PSGGYGS

V4(3) ------------------------------------------------------------

V5(2) QPGASGGYG---A--GYSGQGGYPPQ-----GYGQPGQGPYPPPG---------------

V6(2) QPGATGGYG---A--GYSGQGGYPPQ-----GYGQPGQGPYPPSG--------PSGGHGA

V7(1) QQGAPGAYP--PPAAAGAQGPAYPPG------YGQPVASGGYGGG------------ASG

V8(1) QQGGPGYPP--PPAAAGAQGHAYPPG------YGQPVAAGGYGGG------------ASG

V9(1) QQGATGGFG---GYPSQSGQGVYPPQ------------GPYAPPG--------AAGGYGG

V10(1) QQGGPGAYP--PPAAAGGQGPAYPPG------YGQPGAAGGYGGG------------ASG

V11(1) QQGGPGYPP--PPAAAGAQGHAYPPG------YGQPVAAGGYGGG------------ASG

V12(1) QPG----------------------------------ASGGYGGG------------ASG

V13(1) QPQGPGVYP--PPGAAGPYGGGYPSQPQGPGVYPPPGAAGPYGGGYPSQPQGPGVYPPPG

V14(1) QPQGPGVYP--PPGAAGPYGGGYPSQPQGPGVYPPPGAAGPYGGGYPSQPQGPGVYPPPG

V15(2) QPGATGGYG---A--GYSGQGGYPPQ-----GYGQPGQGPYPPPG--------PSGGYGA

V16(1) QPGATGGYG---A--GYSGQGGYPPQ-----GYGQPGQGPYPPPG--------PSGGYGA

V17(2) QPGASGGYGGGASGGAGPYGGGYPSQ---------PGASGGYGGG------------ASG

V18(1) QPGATGGYG---A--GYSGQGGYPPQ-----GYGQPGQGPYPPSG--------PSGGHGA

V19(1) QPGASGGYG---A--GYSGQGGYPPQ-----GYVQPGQGPYPPSG--------PSGGHGA

V20(1) QPGASGGYG---A--GYSGQGGYPPQ-----GYGQPGQGPYPPPG---------------

V21(1) QPGASGGYG---A--GYSGQGGYPPQ-----GYGQPGQGPYPPPG---------------

V22(3) QPGASGGYG---A--GYSGQGGYPPQ-----GYGQPGQGPYPPPG---------------

V23(1) QPGATGGYG---A--GYSGQGGYPPQ-----GYGQPGQGPYPPPG--------PSGGYGA

V24(1) QPGATGGYG---A--GYSGQGGYPPQ-----GYGQPGQGPYPPPG--------PSGGYGA

V25(1) QPGASGGYG---A--GYSGQGGYPPQ-----GYVQPGQGPYPPSG--------PSGGHGA

V26(1) QPGASGGYG---A--GYSGQGGYPPQ-----GYGQPGQGPYPPSG--------PSGGPGA

V27(2) ------------------------------------------------------------

TP02_0895 AAGGYGGGPPG-PGQPSGPPGQAPGNMQLLKIDVKNPNNGPNIIFEEYIAGKPERRHRQF

V1(3) AAGGYGGGPPG-PGQPSGPPGQAPGNMQLLKIDVKNPNNGPNIIFEEYIAGKPERRHRQF

V2(1) RAGGYGGGKPG-SGHPIDPTGHASG-GNLLKIDVKNPNNGPNIIFEEYIP----------

V3(1) GAGGYGGGEPG-SGHPSGPTGHASG-GNLLKIDVKNPNNGPNIIFEEYIAGKPERRHRQF

V4(3) ------------------------------------------------------------

V5(2) AAGGYGGGPPG-PGQPSGPPGQAPGNMQLLKIDV--------------------------

V6(2) GAGGYGGGAPG-PGQPSGPTGQTHG-GNMLTIDAKTKHNGPNIIVDEYRAGPQDRRHRQF

V7(1) GAGGYGGGYPSQPGGPQGAAG-HPGQPIMMKIDAKNRRN-PNTKLDRFTLSN-GGTQHVF

V8(1) GAGGYGGGYPSQPGGPQGAAG-PPGQPIMMKIDVKNRRN-PNTKLDRFTLTD-GRTQHAF

V9(1) GAGGYGGGASG-PGQPTGPPGHTPG-VNLLTIDAKTKHNGANIIVDEYRAGRPERIHRQF

V10(1) GAGGYGGGYPGQQGGPQGAAG-PPGEPIMVKMDAKSRVN-RDINLTKFTLTN-GKTQHVF

V11(1) GAGGYGGGYPSQPGGPQGAAG-PPGQPIMMKIDVKNRRN-PNTKLDRFTLTD-GRTQHAF

V12(1) GAGPYGGGYPSQPGGPQGAAG-PPGEPIMLKMDAKNRTN-RNINLTKFTLPN-GKTQHVF

V13(1) AAGPYGGGYPSQPGGPQGATG-PPGQPIMMKIDAKNRRN-PNTKLDRFTLSN-GGTQHVF

V14(1) AAGPYGGGYPSQPGGPQGATG-PPGQPIMMKIDAKNRRD-PNTKLDRFTLSN-GGTQHVF

V15(2) GAGGYGGSAPG-PGHPSGPTGHASG-GNLLKIDVKNPNNGP-------------------

V16(1) GAGGYGGSAPG-PGHPSGPTGHASG-GNLLKIDVKNPNNGP-------------------

V17(2) GAGGYGGGYPSQPGGPQGAAG-PPGQPIMMRIDGKNRRN-PNTRLDRFTLPD-GRTQHVF

V18(1) GAGGYGGGAPG-PGQPSGPTGQTHG-GNMLTIDAKTKHNGP-------------------

V19(1) GAGGYGGGAPG-PGQPSGPPGQAPG-GNMLKIDAKSPNNGKNIIVEEFRAGRPERTHRQF

V20(1) AAGGYGGGPPG-PGQPSGPPGQAPGNMQLLTIDAKTKHNGANIIVDEYRAGRPERIHRQF

V21(1) AAGGYGGGPPG-PGQPSGPPGQAPGNMQLLKIDVKNPNNGPNIIFEEYIAGKPERRHRQF

V22(3) AAGGYGGGPPG-PGQPSGPPGQAPGNMQLLKIDVKNPNNGPNIIFEEYIAGKPERRHRQF

V23(1) GAGGYGGGEPG-PGHPSGPTGHASG-GNLLKNRCKKSKQWSK------------------

V24(1) GAGGYGGSAPG-PGHPSGPTGHASG-GNLLKIDVKNPNNGPNIIFEEYIA----------

V25(1) GAGGYGGGAPG-PGQPSGPPGQAPG-GNMLKIDAKSPNNGKNIIVEEFRAGRPERTHRQF

V26(1) GAGGYGGGAPG-PGQPSGPPSQSPDGKNLGPVDGKTGKNDPFVIFERFRAGPQNRVHHQY

V27(2) ------------------------------------------------------------

TP02_0895 TPVPGCGINQVNYDGEKVWSLEVGG-DYAVKVLVFPIGFKEKTIEITFIGGEKEIYKKKG

V1(3) TPVPGCGINQVNYDGEKVWSLEVGG-DYAVKVLVFPIGFKEKTIEITFIGGEKEIYKKKG

V2(1) ------------------------------------------------------------

V3(1) TPVPGCGINQVNYDGEKVW-----------------------------------------

V4(3) ------------------------------------------------------------

V5(2) ------------------------------------------------------------

V6(2) DPVPGCGIIQVNYDGRKVWTA--SGPEHAVKVVVFPLGFKEKTIEITFIGGEKAIYKKPG

V7(1) RPDPGHGIKQVNYDGDRVWSMDRD--NFGTEVLVDPIGSGAKTMTIKMVNGDEVTFTKSG

V8(1) RANPGYAIKQVNYDGDRVWSMDGG--NFGTEVLVDPIGSGAKTMTIKLAN----------

V9(1) DPVPGCGINQVNYNGKPVWKMIG--EKYAVKVVLFPIGFEEKTIVITMIGGENEIYKKKG

V10(1) RANPGHGIKQVNYDGDPVWSMDGN--NFGTEVLLDPIGSPAKNMTIKLANGDKVTFIKKG

V11(1) RANPGYAIKQVNYDGDRVWSMDGG--NFGTEVLVDPIGSGAKTMTIKLANGDNVIYTKPG

V12(1) RANPGHGIKQVNYDGDFVWSMDGG--NFGTEVLVDPIGSGAKTMTIKLANGDKVIHTKPG

V13(1) RPDPGHGIKQVNYDGDPVWSMDGN--NFGTEVLLDPIGSPAKNMTIKMVNGDKEIYKKKG

V14(1) RPDPGHGIKQVNYDGDPVWSMDGN--NFGTEVLLDPIGLQQK-YDIKFIGGEKEIYKKKG

V15(2) ------------------------------------------------------------

V16(1) ------------------------------------------------------------

V17(2) RPNLGYAIKQVNYDGDRVWSMDDD--NFATEVLV--------------------------

V18(1) ------------------------------------------------------------

V19(1) TPVPGCGINQVN------------------------------------------------

V20(1) DPVPGCGINQVNYNGQPVWK-MIG-EKYAVTVVLFPIGFEEKTIVITMIGGEKEIYKKKG

V21(1) TPVPGCGINQVNYDGGNVWTM--NDDIHALKVVVFPIGFKEKTIEITFIGGEKEIYKKKG

V22(3) TPVPGCGINQVN------------------------------------------------

V23(1) ------------------------------------------------------------

V24(1) ------------------------------------------------------------

V25(1) TPVPGCGINQVNYDGRKVWS-LEVGGDHAVKVLVFPIGFKEKTIEITFIGGEKEIYKKKG

V26(1) TPRSGCGFNMVKYSGGIVWILGGDGSNYATKVIVHPVGFKEKTIEITFIGGEKEIYKKKG

V27(2) ------------------------------------------------------------

TP02_0895 RNKPWTKQ

V1(3) RNKPWTKQ

V2(1) --------

V3(1) --------

V4(3) --------

V5(2) --------

V6(2) RNKPWTKQ

V7(1) RNKPWTKQ

V8(1) --------

V9(1) RNKPWTKQ

V10(1) --------

V11(1) RNKPWTKQ

V12(1) RNKPWTKQ

V13(1) RNKPWTKQ

V14(1) RNKPWTKQ

V15(2) --------

V16(1) --------

V17(2) --------

V18(2) --------

V19(1) --------

V20(1) RNKPWTKQ

V21(1) RNKPWTKQ

V22(3) --------

V23(1) --------

V24(1) --------

V25(1) RNKPWTKQ

V26(1) RNKPWTKQ

V27(2) --------

Eptiope region highlighted in grey.

**Table S12a: Tp10 nucleotide variant Clustal alignment**

Tp04_0772(genomic) ATGAAGGTCACAAATCTCAAGTGGAATCCAACTGTT***GACTACGCTCTTCTTACCAC***TTCT 60

XP_764408(mRNA) ATGAAGGTCACAAATCTCAAGTGGAATCCAACTGTTGACTACGCTCTTCTTACCACTTCT 60

V1(3) ------------------------------------------------------------ 0

V2(1) ------------------------------------------------------------ 0

V3(1) ------------------------------------------------------------ 0

V4(1) ------------------------------------------------------------ 0

V5(1)* ------------------------------------------------------------ 0

V6(2) ------------------------------------------------------------ 0

V7(1) ------------------------------------------------------------ 0

V8(1) ------------------------------------------------------------ 0

V9(1)* ------------------------------------------------------------ 0

V10(5) ------------------------------------------------------------ 0

V11(3) ------------------------------------------------------------ 0

V12(1) ------------------------------------------------------------ 0

V13(1)* ------------------------------------------------------------ 0

Tp04_0772(genomic) TTCGACTGTACAGCTAAAGTCTGGTAAGAAAAAAAATTTTCTAAAAAATTTAGGGACGCG 120

XP_764408(mRNA) TTCGACTGTACAGCTAAAGTCTG------------------------------GGACGCG 90

V1(3) ----------------------------------------------------GGGACGCG 8

V2(1) ----------------------------------------------------GGGACGCT 8

V3(1) ----------------------------------------------------GGGACGCG 8

V4(1) ------------------------------------------------------------ 0

V5(1)* ----------------------------------------------------GGGACGCG 8

V6(2) ----------------------------------------------------GGGACGCG 8

V7(1) ----------------------------------------------------GGGACGCG 8

V8(1) ----------------------------------------------------GGGACGCG 8

V9(1)* ----------------------------------------------------GGGACGCG 8

V10(5) ----------------------------------------------------GGGACGCG 8

V11(3) ----------------------------------------------------GGGACGCG 8

V12(1) ----------------------------------------------------GGGACGCG 8

V13(1)* ------------------------------------------------------------ 0

Tp04_0772(genomic) TCAAATGGCAAAGAACTATTTTCAACCTCAATTGGTGAGCATCCTTCTTCATGCTCCTGG 180

XP_764408(mRNA) TCAAATGGCAAAGAACTATTTTCAACCTCAATTGGTGAGCATCCTTCTTCATGCTCCTGG 150

V1(3) TCAAATGGCAAAGAACTATTTTCAACCTCAATTGGTGAGCATCCTTCTTCATGCTCCTGG 68

V2(1) TCAAATGGCAAAGAACTATTTTCAACCTCAATTGGTGAGCATCCTTCTTCATGCTCCTGG 68

V3(1) TCAAATGGCAAAGAACTATTTTCAACCTCAATTGGTGAGCACCCTTCTTCATGCTCCTGG 68

V4(1) ---------------------TCAACCTCAATTGGTGAGCACCCTTCTTCATGCTCCTGG 39

V5(1)* TCAAATGGCAAAGAACTATTTTCAACCTCAATTGGTGAGCACCCTTCTTCATGCTCCTGG 68

V6(2) TCAAATGGCAAAGAACTATTTTCAACCTCAATTGGTGAGCACCCTTCTTCATGCTCCTGG 68

V7(1) TCAAATGGCAAAGAACTATTTTCAACCTCAATTGGTGAGCATCCTTCTTCATGCTCCTGG 68

V8(1) TCAAATGGCAAAGAACTATTTTCAACCTCAATTGGTGAGCATCCTTCTTCATGCTCCTGG 68

V9(1)* TCAAATGGCAAAGAACTATTTTCAACCTCAATTGGTGAGCATCCTTCTTCATGCTCCTGG 68

V10(5) TCAAATGGCAAAGAACTATTTTCAACCTCAATTGGTGAGCATCCTTCTTCATGCTCCTGG 68

V11(3) TCAAATGGCAAAGAACTATTTTCAACCTCAATTGGTGAGCATCCTTCTTCATGCTCCTGG 68

V12(1) TCAAATGGCAAAGAACTATTTTCAACCTCAATTGGTGAGCATCCTTCTTCATGCTCCTGG 68

V13(1)* ------------------------------------------------------------ 0

Tp04_0772(genomic) ACACCTAACGGGTACTTTAAGTTTGGAAATTAATGTAATTTAGAGACAAGATTTTAGTTT 240

XP_764408(mRNA) ACACCTAACGG--------------------------------AGACAAGATTTTAGTTT 178

V1(3) ACACCTAACGGGTACTTTAAGTTTGGAAATTAATGTAATTTAGAGACAAGATTTTAGTTT 128

V2(1) ACACCTAACGGGTACTTTAAGTTTGGAAATTAATGTAATTTAGAGACAAGATTTTAGTTT 128

V3(1) ACACCTAACGGGTACTTTAAGTTTGGAAATTAATGTAATTTAGAGACAAGATTTTAGTTT 128

V4(1) ACACCTAACGGGTACTTTAAGTTTGGAAATTAATGTAATTTAGAGACAAGATTTTAGTTT 99

V5(1)* ACACCTAACGGGTACTTTAAGTTTGGAAATTAATGTAATTTAGAGACAAGATTTTAGTTT 128

V6(2) ACACCTAACGGGTACTTTAAGTTTGGAAATTAATGTAATTTAGAGACAAGATTTTAGTTT 128

V7(1) ACACCTAACGGGTACTTTAAGTTTGGAAATTAATGTAATTTAGAGACAAGATTTTAGTTT 128

V8(1) ACACCTAACGGGTACTTTAAGTTTGGAAATTAATGTAATTTAGAGACAAGATTTTAGTTT 128

V9(1)* ACACCTAACGGGTACTTTAAGTTTGGAAATTAATGTAATTTAAAGACAAAATTTTAGTTT 128

V10(5) ACACCTAACGGGTACTTTAAGTTTGGAAATTAATGTAATTTAGAGACAAGATTTTAGTTT 128

V11(3) ACACCTAACGGGTACTTTATGTTTGGAAATTAATGTAATTTAGAGACAAGATTTTAGTTT 128

V12(1) ACACCTAACGGGTACTTTATGTTTGGAAATTAATGTAATTTAGAGACAAGATTTTAGTTT 128

V13(1)* ---CCTAACGGGTACTTTAAGTTTGGAAATTAATGTAATTTAGAGACAAGATTTTAGTTT 57

******** *****************

Tp04_0772(genomic) CAACCAAAGAAGCGAATGTATCATTAATTGACCCGAGATCAGGAAATTGTTGTAGCAAGT 300

XP_764408(mRNA) CAACCAAAGAAGCGAATGTATCATTAATTGACCCGAGATCAGGAAATTGTTGTAGCAAGT 238

V1(3) CAACCAAAGAAGCGAATGTATCATTAATTGACCCGAGATCAGGAAATTGTTGTAGCAAGT 188

V2(1) CAACCAAAGAAGCAAATGTATCATTAATTGACCCGAGATCAGGAAATTGTTGTAGCAAGT 188

V3(1) CAACCAAAGAAGCTAATGTATCATTAATTGACCCGAGATCAGGAAATTGTTGTAGCAAGT 188

V4(1) CAACCAAAGAAGCGAATGTATCATTAATTGACCCGAGATCAGGAAATTGTTGTAGCAAGT 159

V5(1)* CAACCAAAGAAGCGAATGTATCATTAATTGACCCGAGATCAGGAAATTGTTGTAGCAAGT 188

V6(2) CAACCAAAGAAGCGAATGTATCATTAATTGACCCGAGATCAGGAAATTGTTGTAGCAAGT 188

V7(1) CAACCAAAGAAGCGAATGTATCATTAATCGACCCGAGATCAGGAAATTGTTGTATCAAGT 188

V8(1) CAACCAAAGAAGCGAATGTATCATTAATCGACCCGAGATCAGGAAATTGTTGTATCAAGT 188

V9(1)* CAACCAAAGAAGCGAATGTATCATTAATTGACCCGAGATCAGGAAATTGTTGTAGCAAGT 188

V10(5) CAACCAAAGAAGCGAATGTATCATTAATTGACCCGAGATCAGGAAATTGTTGTAGCAAGT 188

V11(3) CAACCAAAGAAGCGAATGTATCATTAATTGACCCGAGATCAGGAAATTGTTGTAGCAAGT 188

V12(1) CAACCAAAGAAGCGAATGTATCATTAATTGACCCGAGATCAGGAAATTGTTGTAGCAAGT 188

V13(1)* CAACCAAAGAAGCGAATGTATCATTAATTGACCCGAGATCAGGAAATTGTTGTAGCAAGT 117

************* ************** ************************* *****

Tp04_0772(genomic) ATAAGGCTCACGACTCTAATAAGTTGACAAGTGCTTTATGGCTTGGAGGACATTACGGAG 360

XP_764408(mRNA) ATAAGGCTCACGACTCTAATAAGTTGACAAGTGCTTTATGGCTTGGAGGACATTACGGAG 298

V1(3) ATAAGGCTCACGACTCTAATAAGTTGACAAGTGCTTTATGGCTTGGAGGACATTACGGAG 248

V2(1) ATAAGGCTCACGACTCTAATAAGTTGACAAGTGCTTTATGGCTTGGAGGACATTACGGAG 248

V3(1) ATAAGGCTCACGACTCTAATAAGTTGACAAGTGCTTTATGGCTTGGAGGACATTACGGAG 248

V4(1) ATAAGGCTCACGACTCTAATAAGTTGACAAGTGCTTTATGGCTTGGAGGGCATTACGGAG 219

V5(1)* ATAAGGCTCACGACTCTAATAAGTTGACAAGTGCTTTATGGCTTGGAGGACATTACGGAG 248

V6(2) ATAAGGCTCACGACTCTAATAAGTTGACAAGTGCTTTATGGCTTGGAGGACATTACGGAG 248

V7(1) ATAAGGCTCACGACTCTAATAAGTTGACAAGTGCTTTATGGCTTGGAGGACATTACGGAG 248

V8(1) ATAAGGCTCACGACTCTAATAAGTTGACAAGTGCTTTATGGCTTGGAGGACATTACGGAG 248

V9(1)* ATAAGGCTCACGACTCTAATAAGTTGACAAGTGCTTTATGGCTTGGAGGACATTACGGAG 248

V10(5) ATAAGGCTCACGACTCTAATAAGTTGACAAGTGCTTTATGGCTTGGAGGACATTACGGAG 248

V11(3) ATAAGGCTCACGACTCTAATAAGTTGACAAGTGCTTTATGGCTTGGAGGACATTACGGAG 248

V12(1) ATAAGGCTCACGACTCTAATAAGTTGACAAGTGCTTTATGGCTTGGAGGACATTACGGAG 248

V13(1)* ATAAGGCTCACGACTCTAATAAGTTGACAAGTGCTTTATGGCTTGGAGGACATTACGGAG 177

************************************************************

Tp04_0772(genomic) GTGACCACATATTTACAAGTGGTTTTGTTGATAACAAGACTAGACAAATAAGGGTTTGGG 420

XP_764408(mRNA) GTGACCACATATTTACAAGTGGTTTTGTTGATAACAAGACTAGACAAATAAGGGTTTGGG 358

V1(3) GTGACCACATATTTACAAGTGGTTTTGTTGATAACAAGACTAGACAAATAAGGGTTTGGG 308

V2(1) GTGACCACATATTTACAAGTGGTTTTGTTGATAACAAGACTAGACAAATAAGGGTTTGGG 308

V3(1) GTGACCACATATTTACAAGTGGTTTTGTTGATAACAAGACTAGACAAATAAGGGTTTGGG 308

V4(1) GTGACCACATATTTACAAGTGGTTTTGTTGATAACAAGACTAGACAAATAAGGGTTTGGG 279

V5(1)* GTGACCACATATTTACAAGTGGTTTTGTTGATAACAAGACTAGACAAATAAGGGTTTGGG 308

V6(2) GTGACCACATATTTACAAGTGGTTTTGTTGATAACAAGACTAGACAAATAAGGGTTTGGG 308

V7(1) GTGACCACATATTTACAAGTGGTTTTGTTGATAACAAGACTAGACAGATAAGGGTTTGGG 308

V8(1) GTGACCACATATTTACAAGTGGTTTTGTTGATAACAAGACTAGACAGATAAGGGTTTGGG 308

V9(1)* GTGACCACATATTTACAAGTGGTTTTGTTGATAACAAGACTAGACAAATAAGGGTTTGGG 308

V10(5) GTGACCACATATTTACAAGTGGTTTTGTTGATAACAAGACTAGACAAATAAGGGTTTGGG 308

V11(3) GTGACCACATATTTACAAGTGGTTTTGTTGATAACAAGACTAGACAAATAAGGGTTTGGG 308

V12(1) GTGACCACATATTTACAAGTGGTTTTGTTGATAACAAGACTAGACAAATAAGGGTTTGGG 308

V13(1)* GTGACCACATATTTACAAGTGGTTTTGTTGATAACAAGACTAGACAAATAAGGGTTTGGG 237

************************************************************

Tp04_0772(genomic) ACACGAGGAAACTCGAGAAACACTTGATTTCCAATGATATTGACAGTTCGCCTTCTCCCT 480

XP_764408(mRNA) ACACGAGGAAACTCGAGAAACACTTGATTTCCAATGATATTGACAGTTCGCCTTCTCCCT 418

V1(3) ACACGAGGAAACTCGAGAAACACTTGATTTCCAATGATATTGACAGTTCGCCTTCTCCCT 368

V2(1) ACACGAGGAAACTCGAGAAACACTTGATTTCCAATGATATTGACAGTTCGCCTTCTCCCT 368

V3(1) ACACGAGGAAACTCGAGAAACACTTGATTTCCAATGATATTGACAGTTCGCCTTCTCCCT 368

V4(1) ACACGAGGAAACTCGAGAAACACTTGATTTCCAATGATATTGACAGTTCGCCTTCTCCCT 339

V5(1)* ACACGAGGAAACTCGAGAAACACTTGATTTCCAATGATATTGACAGTTCGCCTTCTCCCC 368

V6(2) ACACGAGGAAACTCGAGAAACACTTGATTTCCAATGATATTGACAGCTCGCCTTCTCCCT 368

V7(1) ACACGAGGAAACTCGAGAAACACTTTATTTCCAATGATATTGACAGCTCGCCTTCTCCCT 368

V8(1) ACACGAGGAAACTCGAGAAACACTTGATTTCCAATGATATTGACAGCTCGCCTTCTCCCT 368

V9(1)* ACACGAGGAAACTCGAGAAACACTTGATTTCCAATGATATTGACAGTTCGCCTTCTCCCT 368

V10(5) ACACGAGGAAACTCGAGAAACACTTGATTTCCAATGATATTGACAGTTCGCCTTCTCCCT 368

V11(3) ACACGAGGAAACTCGAGAAACACTTGATTTCCAATGATATTGACAGCTCGCCTTCTCCCT 368

V12(1) ACACGAGGAAACTCGAGAAACACTTGATTTCCAATGATATTGACAGCTCGCCTTCTCCCT 368

V13(1)* ACACGAGGAAACTCGAGAAACACTTGATTTCCAATGATATTGACAGCTCGCCTTCTCCCT 297

********************************************** ************

Tp04_0772(genomic) TGATTCCACACTGGGATCCTCAAATTGGGTTGGTTGTCCTCGCTTCAAAGGTTAATTTCT 540

XP_764408(mRNA) TGATTCCACACTGGGATCCTCAAATTGGGTTGGTTGTCCTCGCTTCAAAGG--------- 469

V1(3) TGATTCCACACTGGGATCCTCAAATTGGGTTGGTTGTCCTCGCTTCAAAGGTTAATTTCT 428

V2(1) TGATTCCACACTGGGATCCCCAAATTGGGTTGGTTGTCCTCGCTTCAAAGGTTAATTTCT 428

V3(1) TGATTCCACACTGGGATCCTCAAATTGGGTTGGTTGTCCTCGCTTCGAAGGTTAATTTCT 428

V4(1) TGATTCCACACTGGGATCCTCAAATTGGGTTGGTTGTCCTCGCTTCAAAGGTTAATTTCT 399

V5(1)* TGATTCCACACTGGGATCCTCAAATTGGGTTGGTTGTCCTCGCTTCAAAGGTTAATTTCT 428

V6(2) TGATTCCACACTGGGATCCTCAAATTGGGTTGGTTGTCCTCGCTTCAAAGGTTAATTTCT 428

V7(1) TGATTCCACACTGGGATCCCCAAATTGGGTTGGTTGTCCTCGCTTCAAAGGTTAATTTCT 428

V8(1) TGATTCCACACTGGGATCCCCAAATTGGGTTGGTTGTCCTCGCTTCAAAGGTTAATTTCT 428

V9(1)* TGATTCCACACTGGGATCCTCAAATTGGGTTGGTTGTCCTCGCTTCAAAGGTTAATTTCT 428

V10(5) TGATTCCACACTGGGATCCTCAAATTGGGTTGGTTGTCCTCGCTTCAAAGGTTAATTTCT 428

V11(3) TGATTCCACACTGGGATCCTCAAATTGGGTTGGTTGTCCTCGCTTCAAAGGTTAATTTCT 428

V12(1) TGATTCCACACTGGGATCCTCAAATTGGGTTGGTTGTCCTCGCTTCAAAGGTTAATTTCT 428

V13(1)* TGATTCCACACTGGGATCCTCAAATTGGGTTGGTTGTCCTCGCTTCGAAGGTTAATTTCT 357

******************* ************************** ****

Tp04_0772(genomic) TCATTAACAAATT-TCTTTAGGGAGACTTAACAGTCAGAATCTTCCAATACATTGATAAG 599

XP_764408(mRNA) ----------------------GAGACTTAACAGTCAGAATCTTCCAATACATTGATAAG 507

V1(3) TCATTAACAAATT-TCTTTAGGGAGACTTAACAGTCAGAATCTTCCAATACATTGATAAG 487

V2(1) TCATTAACAAATT-TCTTTAGGGAGACTTAACAGTCAGAATCTTCCAATACATTGATAAG 487

V3(1) TTATTAACAAATT-TCTTTAGGGAGACTTAACAGTCAGAATCTTCCAATACATTGATAAG 487

V4(1) TCATTAAAA-ATT-TCTTTAGGGAGACTTAACAGTCAGAATCTTCCAATACATTGATAAG 457

V5(1)* TCATTAACAAATT-TTTTTAGGGAGACTTAACAGTCAGAATCTTCCAATACATTGATAAG 487

V6(2) TCATTAACAAATT-TCTTTAGGGAGACTTAACAGTCAGAATCTTCCAATACATTGATAAG 487

V7(1) TCATTAACAAATT-TCTTTAGGGAGACTTAACAGTCAGAATCTTCCAATACATTGATAAG 487

V8(1) TCATTAACAAATT-TCTTTAGGGAGACTTAACAGTCAGAATCTTCCAATACATTGATAAG 487

V9(1)* TCATTAACAAATT-TCTTTAGGGAGACTTAACAGTCAGAATCTTCCAATACATTGATAAG 487

V10(5) TCATTAACAAATT-TCTTTAGGGAGACTTAACAGTCAGAATCTTCCAATACATTGATAAG 487

V11(3) TCATTAACAAATT-TTTTTAGGGAGACTTAACAGTCAGAATCTTCCAATACATTGATAAG 487

V12(1) TCATTAACAAATT-TTTTTAGGGAGACTTAACAGTCAGAATCTTCCAATACATTGATAAG 487

V13(1)* TTATTAACAAATT-TCTTTAGGGAGACTTAACAGTCAGAATCTTCCAATACATTGATAAG 416

**************************************

Tp04_0772(genomic) GAGCTAAACAGGGCGGGAGAGTTCAAAGCCACAGGTTCCATGAAGTCCTTCTGTTTAGTA 659

XP_764408(mRNA) GAGCTAAACAGGGCGGGAGAGTTCAAAGCCACAGGTTCCATGAAGTCCTTCTGTTTAGTA 567

V1(3) GAGCTAAACAGGGCGGGAGAGTTCAAAGCCACAGGTTCCATGAAGTCCTTCTGTTTAGTA 547

V2(1) GAGCTAAACAGGGCGGGAGAATTCAAAGCCACAGGTTCCATGAAGTCCTTCTGTTTAGTA 547

V3(1) GAGCTAAACAGGGCGGGAGAGTTCAAAGCCACAGGTTCCATGAAGTCCTTCTGTTTAGTA 547

V4(1) GAGCTAAACAGGGCGGGAGAGTTCAAAGCCACGGGTTCCATGAAGTCCTTCTGTTTAGTA 517

V5(1)* GAGCTAAACAGGGCGGGA------------------------------------------ 505

V6(2) GAGCTAAACAGGGCGGGAGAGTTCAAAGCCACAGGTTCCATGAAGTCCTTCTGTTTAGTA 547

V7(1) GAGCTAAACAGGGCGGGAGAGTTCAAAGCCACGGGTTCCATGAAGTCCTTCTGTTTAGTA 547

V8(1) GAGCTAAACAGGGCGGGAGAGTTCAAAGCCACGGGTTCCATGAAGTCCTTCTGTTTAGTA 547

V9(1)* GAGCTAAACAGGGCGGGAGAGTTCAAAGCCACAGGTTCCATGAAGTCCTTCTGTTTAGTA 547

V10(5) GAGCTAAACAGGGCGGGAGAGTTCAAAGCCACAGGTTCCATGAAGTCCTTCTGTTTAGTA 547

V11(3) GAGCTAAACAGGGCGGGAGAGTTCAAAGCCACAGGTTCCATGAAGTCCTTCTGTTTAGTA 547

V12(1) GAGCTAAACAGGGCGGGAGAGTTCAAAGCCACAGGTTCCATGAAGTCCTTCTGTTTAGTA 547

V13(1)* GAGCTAAACAGGGCGGGAGAGTTCAAAGCCACAGGTTCCATGAAGTCCTTCTGTTTAGTA 476

******************

Tp04_0772(genomic) CCCACTGATATCTGTGATAAATCGAAGTGTGAATTAGGAAGGTTCTTGTTCAATACTGAT 719

XP_764408(mRNA) CCCACTGATATCTGTGATAAATCGAAGTGTGAATTAGGAAGGTTCTTGTTCAATACTGAT 627

V1(3) CCCACTGATATCTGTGATAAATCGAAGTGTGAATTAGGAAGGTTCTTGTTCAATACTGAT 607

V2(1) CCCACTGATATCTGTGATAAATCGAAGTGTGAATTAGGAAGGTTCTTGTTCAATACTGAT 607

V3(1) CCCACTGATATCTGTGATAAATCGAAGTGTGAATTAGGAAGGTTCTTGTTCAATACTGAT 607

V4(1) CCCACTGATATCTGTGATAAATCGAAGTGTGAATTAGGAAGGTTCTTGTTCAATACTGAT 577

V5(1)* ------------------------------------------------------------ 505

V6(2) CCCACTGATATCTGTGATAAATCGAAGTGTGAATTAGGAAGGTTCTTGTTCAATACTGAT 607

V7(1) CCCACTGATATCTGTGATAAATCGAAGTGTGAATTAGGAAGGTTCTTGTTCAATACTGAT 607

V8(1) CCCACTGATATCTGTGATAAATCGAAGTGTGAATTAGGAAGGTTCTTGTTCAATACTGAT 607

V9(1)* CCCACTGATATCTGTGATAAATCGAAGTGTGAATTAGGAAGGTTCTTGTTCAATACTGAT 607

V10(5) CCCACTGATATCTGTGATAAATCGAAGTGTGAATTAGGAAGGTTCTTGTTCAACACTGAT 607

V11(3) CCCACTGATATCTGTGATAAATCGAAGTGTGAATTAGGAAGGTTCTTGTTCAATACTGAT 607

V12(1) CCCACTGATATCTGTGATAAATCGAAGTGTGAATTAGGAAGGTTCTTGTTCAATACTGAT 607

V13(1)* CCCACTGATATCTGTGATAAATCGAAGTGTGAATTAGGAAGGTTCTTGTTCAATACTGAT 536

Tp04_0772(genomic) TGCAAGCAGATAAATCTTACATCCATGTTCATCATAAGGAGGAATTCTGCAACAACAATG 779

XP_764408(mRNA) TGCAAGCAGATAAATCTTACATCCATGTTCATCATAAGGAGGAATTCTGCAACAACAATG 687

V1(3) TGCAAGCAGATAAATCTTACATCCATGTTCATCATAAGGAGGAATTCTGCAACAACAATG 667

V2(1) TGCAAGCAGATAAATCTTACATCCATGTTCATCATAAGGAGGAATTCTGCAACAACAATG 667

V3(1) TGCAAGCAGATAAATCTTACATCCATGTTCATCATAAGGAGGAATTCTGCAACAACAATG 667

V4(1) TGCAAGCAGATAAATCTTACATCCATGTTCATCATAAGGAGGAATTCTGCAACAACAATG 637

V5(1)* ------------------------------------------------------------ 505

V6(2) TGCAAGCAGATAAATCTTACATCCATGTTCATCATAAGGAGGAATTCTGCAACAACAATG 667

V7(1) TGCAAGCAGATAAATCTTACATCCATGTTCATCATAAGGAGGAATTCTGCAACAACAATG 667

V8(1) TGCAAGCAGATAAATCTTACATCCATGTTCATCATAAGGAGGAATTCTGCAACAACAATG 667

V9(1)* TGCAAGCAGATAAATCTTACATCCATGTTCATCATAAGGAGGAATTCTGCAACAACAATG 667

V10(5) TGCAAGCAGATAAATCTTACATCCATGTTCATCATAAGGAGGAATTCTGCAACAACAATG 667

V11(3) TGCAAGCAGATAAATCTTACATCCATGTTCATCATAAGGAGGAATTCTGCAACAACAATG 667

V12(1) TGCAAGCAGATAAATCTTACATCCATGTTCATCATAAGGAGGAATTCTGCAACAACAATG 667

V13(1)* TGCAAGCAGATAAATCTTACATCCATGTTCATCATAAGGAGGAATTCTGCAACAACAATG 596

Tp04_0772(genomic) AACGAACTTTACGGAGAGGAGTACGATACTGCAAGGTATTCAGTCCGTGACTGGGAAGCA 839

XP_764408(mRNA) AACGAACTTTACGGAGAGGAGTACGATACTGCAAGGTATTCAGTCCGTGACTGGGAAGCA 747

V1(3) AACGAACTTTACGGAGAGGAGTACGATACTGCAAGGTATTCAGTCCGTGACTGGGAAGCA 727

V2(1) AACGAACTTTACGGAGAGGAGTATGATACTGCAAGGTATTCAGTCCGTGACTGGGAAGCA 727

V3(1) AACGAACTTTACGGAGAGGAGTACGATACTGCAAGGTATTCAGTCCGTGACTGGGAAGCA 727

V4(1) AACGAACTTTACGGAGAGGAGTACGATACTGCAAGGTATTCAGTCCGTGACTGGGAAGCA 697

V5(1)* ------------------------------------------------------------ 505

V6(2) AACGAACTTTACGGAGAGGAGTACGATACTGCAAGGTATTCAGTCCGTGACTGGGAAGCA 727

V7(1) AACGAACTTTACGGAGAGGAGTACGATACTGCAAGGTATTCAGTCCGTGACTGGGAAGCA 727

V8(1) AACGAACTTTACGGAGAGGAGTACGATACTGCAAGGTATTCAGTCCGTGACTGGGAAGCA 727

V9(1)* AACGAACTTTA------------------------------------------------- 678

V10(5) AACGAACTTTACGGAGAGGAGTACGATACTGCAAGGTATTCAGTCCGTGACTGGGAAGCA 727

V11(3) AACGAACTTTACGGAGAGGAGTACGATACTGCAAGGTATTCAGTCCGTGACTGGGAAGCA 727

V12(1) AACGAACTTTACGGAGAGGAGTACGATACTGCAAGGTATTCAGTCCGTGACTGGGAAGCA 727

V13(1)* AACGAACTTTACGGAGAGGAGTACGATACTGCAAGGTATTCAGTCCGTGACTGGGAAGCA 656

Tp04_0772(genomic) GGACTTGAGAAGAACTTGGGTCGTCTGACAATAACCAAAAACAAATGCGCCCAAATCATC 899

XP_764408(mRNA) GGACTTGAGAAGAACTTGGGTCGTCTGACAATAACCAAAAACAAATGCGCCCAAATCATC 807

V1(3) GGACTTGAGAAGAACTTGGGTCGTCTGACAATAACCAAAAACAAATGCGCCCAAATCATC 787

V2(1) GGACTTGAGAAGAACTTGGGTCGTCTGACAATAACCAAAAACAAATGCGCCCAAATCATC 787

V3(1) GGACTTGAGAAGAACTTGGGTCGTCTGACAATAACCAAAAACAAATGCGCCCAAATCATC 787

V4(1) GGACTTGAGAAGAACTTGGGTCGTCTGACAATAACCAAGAACAAATGCGCCCAAATCATC 757

V5(1)* ------------------------------------------------------------ 505

V6(2) GGACTTGAGAAGAACTTGGGTCGTCTGACAATAACCAAAAACAAATGCGCCCAAATCATC 787

V7(1) GGACTTGAGAAGAACTTGGGTCGTCTGACAATAACCAAAAACAAATGCGCGCAAATCATC 787

V8(1) GGACTTGAGAAGAACTTGGGTCGTCTGACAATAACCAAAAACAAATGCGCGCAAATCATC 787

V9(1)* ------------------------------------------------------------ 678

V10(5) GGACTTGAGAAGAACTTGGGTCGTCTGACAATAACCAAAAACAAATGCGCCCAAATCATC 787

V11(3) GGACTTGAGAAGAACTTGGGTCGTCTGACAATAACCAAAAACAAATGCGCCCAAATCATC 787

V12(1) GGACTTGAGAAGAACTTGGGTCGTCTGACAATAACCAAAAACAAATGCGCCCAAATCATC 787

V13(1)* GGACTTGAGAAGAACTTGGGTCGTCTGACAATAACCAAAAACAAATGCGCCCAAATCATC 716

Tp04_0772(genomic) AAAGATAAGGTTTGTGCAGAATCTTTATATTACTTTAGGATGCTGGATGCAAGTTCAGGG 959

XP_764408(mRNA) AAAGATAAGGTTTGTGCAGAATCTTTATATTACTTTAGGATGCTGGATGCAAGTTCAGGG 867

V1(3) AAAGATAAGGTTTGTGCAGAATC------------------------------------- 810

V2(1) AAAGATAAGGTTTGTGCAGA---------------------------------------- 807

V3(1) AAAGATAAGGTTTGTGCAGTATCGTTATATTACTT------------------------- 822

V4(1) AAAGATAAGGTTTGTG-------------------------------------------- 773

V5(1)* ------------------------------------------------------------ 505

V6(2) AAAGATAAGGTTTGTGCCGAATCGTTATATTACTTA------------------------ 823

V7(1) AAAGATAAGGTTTGTGCAGAATCGTTATATT----------------------------- 818

V8(1) AAAGATAAGGTTTGTGCAGAATCGTTATATTACTTTA----------------------- 824

V9(1)* ------------------------------------------------------------ 678

V10(5) AAAGATAAGGTTTGTGCAGAATCGTTATATTACTTTA----------------------- 824

V11(3) AAAGATAAGGTTTGTGCAGAATCCTTATATTACTTTA----------------------- 824

V12(1) AAAGATAAGGTTTGTGCAGAATCCTTATATTACTTAG----------------------- 824

V13(1)* AAAGATAAGGTTTGTGCAG----------------------------------------- 735

Tp04_0772(genomic) AGATTTCCAAAA***CCATCCAGGAGTGTAATGCC***AAATACGGAACCAACTTTAACAATCCAG 1019

XP_764408(mRNA) AGATTTCCAAAACCATCCAGGAGTGTAATGCCAAATACGGAACCAACTTTAACAATCCAG 927

V1(3) ------------------------------------------------------------ 810

V2(1) ------------------------------------------------------------ 807

V3(1) ------------------------------------------------------------ 822

V4(1) ------------------------------------------------------------ 773

V5(1)* ------------------------------------------------------------ 505

V6(2) ------------------------------------------------------------ 823

V7(1) ------------------------------------------------------------ 818

V8(1) ------------------------------------------------------------ 824

V9(1)* ------------------------------------------------------------ 678

V10(5) ------------------------------------------------------------ 824

V11(3) ------------------------------------------------------------ 824

V12(1) ------------------------------------------------------------ 824

V13(1)* ------------------------------------------------------------ 735

Primer regions are in bold, underlined and italicized text.

* Partial sequence as defined by only having either the forward or reverse sequence of the allele available for analysis. This sequence is its own variant due to unique nucleotides.

**Table S12b: Tp10 protein variant Clustal alignment**

XP_764408 MKVTNLKWNPTVDYALLTTSFDCTAKVWDASNGKELFSTSIGEHPSSCSWTPNGDKILVS 60

V1(16) ----------------------------DASNGKELFSTSIGEHPSSCSWTPNGDKILVS 32

V2(1) --------------------------FRDASNGKELFSTSIGEHPSSCSWTPNGDKILVS 34

V3(1) ----------------------------DASNGKELFSTSIGEHPSSCSWTPNGDKILVS 32

V4(1) --------------------------FRDASNGKELFSTSIGEHPSSCSWTPNGDKILVS 34

V5(2) ----------------------------DASNGKELFSTSIGEHPSSCSWTPNGDKILVS 32

V6(1) --------------------------FRDASNGKELFSTSIGEHPSSCSWTPNGDKILVS 34

********************************

XP_764408 TKEANVSLIDPRSGNCCSKYKAHDSNKLTSALWLGGHYGGDHIFTSGFVDNKTRQIRVWD 120

V1(16) TKEANVSLIDPRSGNCCSKYKAHDSNKLTSALWLGGHYGGDHIFTSGFVDNKTRQIRVWD 92

V2(1) TKEANVSLIDPRSGNCCIKYKAHDSNKLTSALWLGGHYGGDHIFTSGFVDNKTRQIRVWD 94

V3(1) TKEANVSLIDPRSGNCCIKYKAHDSNKLTSALWLGGHYGGDHIFTSGFVDNKTRQIRVWD 92

V4(1) TKEANVSLIDPRSGNCCSKYKAHDSNKLTSALWLGGHYGGDHIFTSGFVDNKTRQIRVWD 94

V5(2) TKEANVSLIDPRSGNCCSKYKAHDSNKLTSALWLGGHYGGDHIFTSGFVDNKTRQIRVWD 92

V6(1) TKEANVSLIDPRSGNCCSKYKAHDSNKLTSALWLGGHYGGDHIFTSGFVDNKTRQIRVWD 94

***************** ******************************************

XP_764408 TRKLEKHLISNDIDSSPSPLIPHWDPQIGLVVLASKGDLTVRIFQYIDKELNRAGEFKAT 180

V1(16) TRKLEKHLISNDIDSSPSPLIPHWDPQIGLVVLASKGDLTVRIFQYIDKELNRAGEFKAT 152

V2(1) TRKLEKHFISNDIDSSPSPLIPHWDPQIGLVVLASKGDLTVRIFQYIDKELNRAGEFKAT 154

V3(1) TRKLEKHLISNDIDSSPSPLIPHWDPQIGLVVLASKGDLTVRIFQYIDKELNRAGEFKAT 152

V4(1) TRKLEKHLISNDIDSSPSPLIPHWDPQIGLVVLASKGDLTVRIFQYIDKELNRAGEFKAT 154

V5(2) TRKLEKHLISNDIDSSPSPLIPHWDPQIGLVVLASKGDLTVRIFQYIDKELNRAGEFKAT 152

V6(1) TRKLEKHLISNDIDSSPSPLIPHWDPQIGLVVLASKGDLTVRIFQYIDKELNRAGEFKAT 154

*******:****************************************************

XP_764408 GSMKSFCLVPTDICDKSKCELGRFLFNTDCKQINLTSMFIIRRNSATTMNELYGEEYDTA 240

V1(16) GSMKSFCLVPTDICDKSKCELGRFLFNTDCKQINLTSMFIIRRNSATTMNELYGEEYDTA 212

V2(1) GSMKSFCLVPTDICDKSKCELGRFLFNTDCKQINLTSMFIIRRNSATTMNELYGEEYDTA 214

V3(1) GSMKSFCLVPTDICDKSKCELGRFLFNTDCKQINLTSMFIIRRNSATTMNELYGEEYDTA 212

V4(1) GSMKSFCLVPTDICDKSKCELGRFLFNTDCKQINLTSMFIIRRNSATTMNELYGEEYDTA 214

V5(2) GSMKSFCLVPTDICDKSKCELGRFLFNTDCKQINLTSMFIIRRNSATTMNELYGEEYDTA 212

V6(1) GSMKSFCLVPTDICDKSKCELGRFLFNTDCKQINLTSMFIIRRNSATTMNELYGEEYDTA 214

******************************************************** *

XP_764408 RYSVRDWEAGLEKNLGRLTITKNKCAQIIKDKVCAESLYYFRMLDASSGRFPKPSRSVMP 300

V1(16) RYSVRDWEAGLEKNLGRLTITKNKCAQIIKDKVCAESLYYFR------------------ 254

V2(1) RYSVRDWEAGLEKNLGRLTITKNKCAQIIKDKVCAESLY--------------------- 253

V3(1) RYSVRDWEAGLEKNLGRLTITKNKCAQIIKDKVCAESLYYF------------------- 253

V4(1) RYSVRDWEAGLEKNLGRLTITKNKCAQIIKDKVCAVSLYY-------------------- 254

V5(2) RYSVRDWEAGLEKNLGRLTITKNKCAQIIKDKVCAESLYYL------------------- 253

V6(1) RYSVRDWEAGLEKNLGRLTITKNKCAQIIKDKVCAEP----------------------- 251

***********************************

Eptiope region lies outside of sequenced region.
